# Supplementary material for: Integrated systems biology approach identifies gene targets for endothelial dysfunction
Source: Mol Syst Biol. 2023 Nov 30;19(12):e11462. doi: 10.15252/msb.202211462 (PMC10698507; doi:10.15252/msb.202211462)
Supplement: Supplementary file 1 — Appendix [file MSB-19-e11462-s015.pdf]

Integrated Systems Biology Approach Identifies Gene Targets for Endothelial Dysfunction

Iguaracy Pinheiro-de-Sousa[1,2+], Miriam Helena Fonseca-Alaniz[1+], Girolamo Giudice[2], Iuri Cordeiro Valadão[1], Silvestre Massimo Modestia[1], Sarah Viana Mattioli[1,3], Ricardo Rosa Junior[1], Lykourgos-Panagiotis Zalmas[4,5], Yun Fang[6], Evangelia Petsalaki[2*], José Eduardo Krieger[1*]

[1]Laboratory of Genetics and Molecular Cardiology, Heart Institute (InCor)/University of São Paulo Medical School, São Paulo, SP, Brazil.
[2]European Molecular Biology Laboratory, European Bioinformatics Institute, Hinxton, UK
[3]Department of Biophysics and Pharmacology, Institute of Biosciences of Botucatu, Universidade Estadual Paulista, Botucatu, São Paulo, Brazil
[4]Wellcome Trust Sanger Institute, Wellcome Trust Genome Campus, Cambridge, CB10 1SA, UK
[5]Open Targets, Wellcome Genome Campus, Hinxton, Cambridge, CB10 1SA, UK.
[6]Department of Medicine, University of Chicago, Chicago, IL, United States

+ these authors contributed equally to the study

* correspondence should be addressed to Evangelia Petsalaki: petsalaki@ebi.ac.uk and Jose E. Krieger: j.krieger@hc.fm.usp.br

Appendix Files

Table of Contents

Appendix Figure S1. Molidustat and EGLN1-3 siRNA effects. (Page 2)

Appendix Figure S2. Extended images of all immunoblotting membranes used in this study. (Page 4)

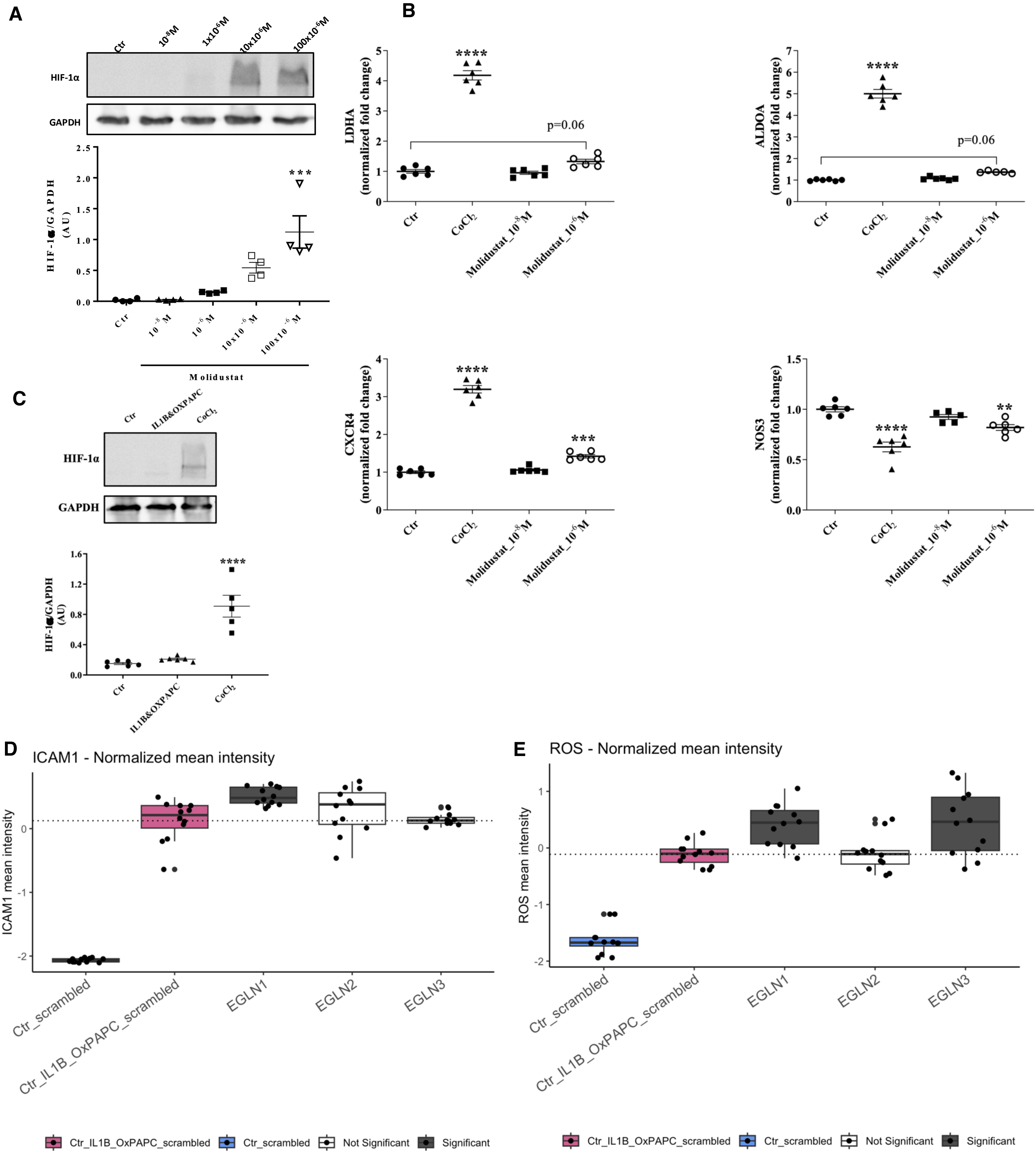
A
Ctr
10-8M
1x10-6M
10x10-6M
100x10-6M
HIF-1α
GAPDH
HIF-1α/GAPDH (AU)
***
Molidustat
B
LDHA (normalized fold change)
ALDOA (normalized fold change)
CXCR4 (normalized fold change)
NOS3 (normalized fold change)
****
***
**
p=0.06
Ctr
CoCl2
Molidustat_10-8M
Molidustat_10-6M
C
Ctr
IL1B&OXPAPC
CoCl2
HIF-1α
GAPDH
HIF-1α/GAPDH (AU)
****
D
ICAM1 - Normalized mean intensity
ICAM1 mean intensity
E
ROS - Normalized mean intensity
ROS mean intensity
Ctr_scrambled
Ctr_IL1B_OxPAPC_scrambled
EGLN1
EGLN2
EGLN3
Ctr_IL1B_OxPAPC_scrambled
Ctr_scrambled
Not Significant
Significant

***Appendix Figure S1. Molidustat and EGLN1-3 siRNA effects**.*

***(A)** Representative image of immunoblotting membrane and quantification of HIF-1α protein expression in HAEC treated with Molidustat with a range of doses from 10^{-8}M to $100x10^{-6}$M for 48h. Data are presented as mean ± mean standard error. ***$p<0.001$ vs. Ctr. GAPDH was used as a housekeeping protein. The experiment was repeated 2 times independently (biological replicates), with 2 technical replicates from each condition.*

***(B)** HAEC were treated with Molidustat at 10^{-8}M and 10^{-6}M, or Cobalt Chloride ($CoCl_2$, 300µM) for 48h and gene expression of Lactate Dehydrogenase A (LDHA), Aldolase Fructose-Bisphosphate A (ALDOA), C-X-C Motif Chemokine Receptor 4 (CXCR4) and Nitric Oxide Synthase 3 (NOS3) were evaluated by qPCR. Data are presented as mean ± mean standard error. ****$p<0.0001$, ***$p<0.001$, **$p<0.01$ vs. Ctr. The experiment was repeated 2 times independently (biological replicates), with 3 technical replicates from each condition.*

***(C)** Representative image of immunoblotting membrane and quantification of HIF-1α protein expression in HAEC treated with IL-1β and OxPAPC or Cobalt Chloride ($CoCl_2$, 300µM) for 48h. Data are presented as mean ± mean standard error. ****$p<0.0001$ vs. Ctr. GAPDH was used as a housekeeping protein. The experiment was repeated 2 times independently (biological replicates), with 3 technical replicates from each condition.*

***(D-E)** Quantification of (D) ICAM intensity and (E) CM-H2DCFDA (ROS indicator) intensity in HAEC transfected with Scrambled siRNA or siRNA to EGLN1, EGLN2 and EGLN3, and treated or not with IL-1β and OxPAPC. The data are represented as a Z-score (Material and Methods). The boxplot depicts the median within the 25th and 75th percentiles, which the whisker extends no further than 1.5 * IQR (Interquartile Range). p-values were computed by multiple pairwise comparisons with the Wilcoxon test followed by BH correction. The comparisons were made between HAECs transfected with EGLN1-3 siRNA versus HAEC transfected with Scrambled siRNA and treated with IL-1β and OxPAPC. The experiment was repeated 4 times independently (biological replicates), with 4 technical replicates from each condition.*

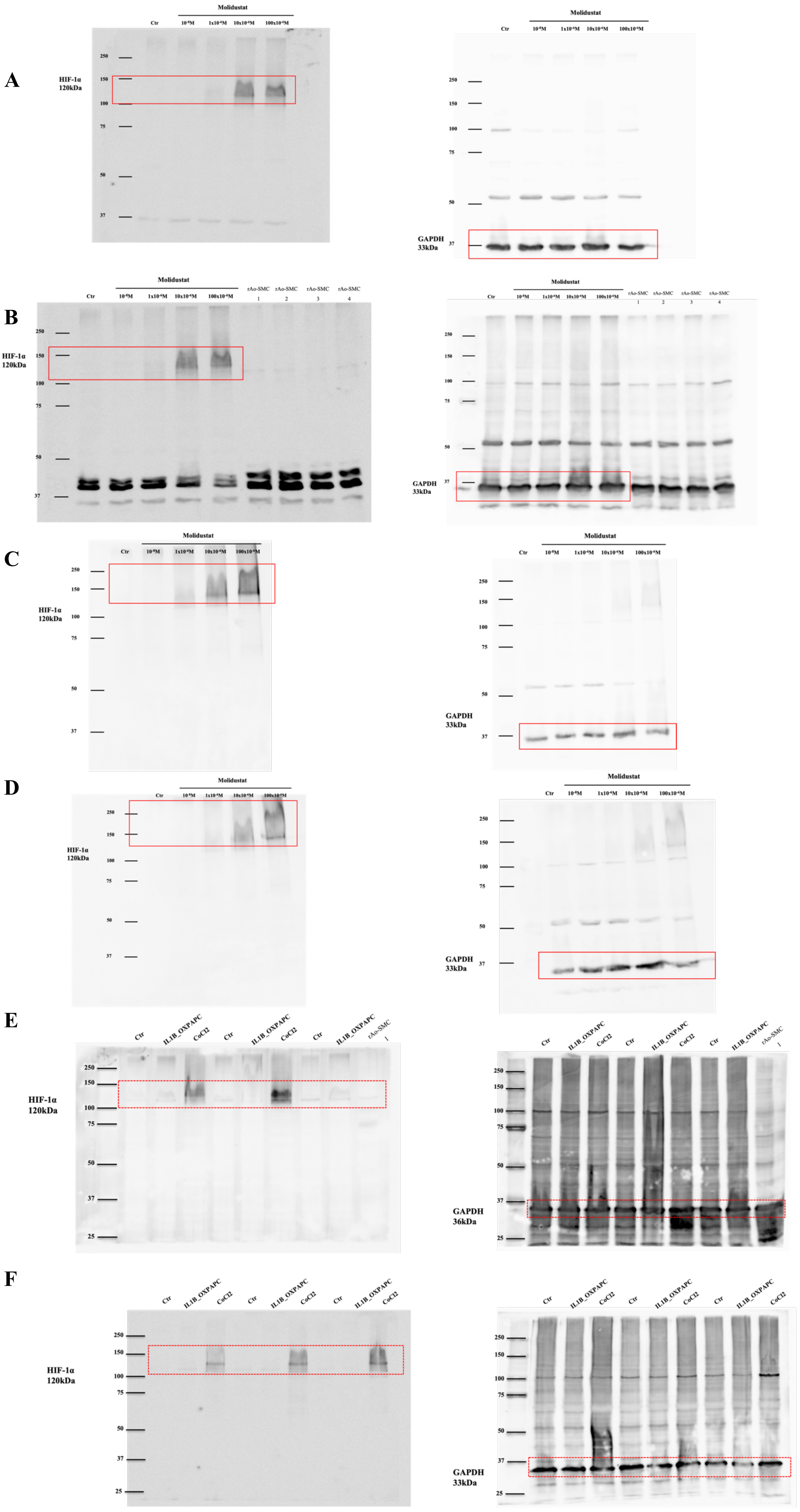
A
Molidustat
Ctr
10⁻⁹M
1x10⁻⁶M
10x10⁻⁶M
100x10⁻⁶M
HIF-1α
120kDa
GAPDH
33kDa
250
150
100
75
50
37
B
rAo-SMC
1
2
3
4
C
D
E
IL1B_OXPAPC
CoCl2
GAPDH
36kDa
25
F

Appendix Figure S2. Extended images of all immunoblotting membranes used in this study.

(A-D) *Images relative to Appendix Figure S1A.*

(E-F) *Images relative to Appendix Figure S1C. rAo-SMC= rat aorta smooth muscle cell*

.
