# Supplementary material for: Integrated systems biology approach identifies gene targets for endothelial dysfunction
Source: Mol Syst Biol. 2023 Nov 30;19(12):e11462. doi: 10.15252/msb.202211462 (PMC10698507; doi:10.15252/msb.202211462)
Supplement: Supplementary file 2 — Expanded View Figures PDF [file MSB-19-e11462-s005.pdf]

Expanded View Figures

Figure EV1. Mimics of CV risk factors OSS, IL-1β, TNF-α and OxPAPC modulate common regulatory regions on the genome.

A Venn diagram of ATAC-seq peaks of all conditions.
B Venn diagram of ChIP-seq peaks of all conditions.
C Venn diagram of the overlapped peaks of ATAC-seq and ChIP-seq.
D Hierarchical clustering profile reveals detailed relationships between all samples under IL-1β, TNF-α, OSS, OxPAPC, basal control and LSS conditions. The heat map of all samples using Euclidean distance as a measured parameter.
E Venn diagram of 356 DEGs identified under the annotated 6,630 peaks.
F Enriched motifs in the centre of enhancer-like regions are shown. The transcription factor (TF) family, motif sequence and enrichment log *P*-values. Enrichment was calculated from a 200 bp sequence, centred on chromatin accessibility.

Source data are available online for this figure.

 © 2023 The Authors

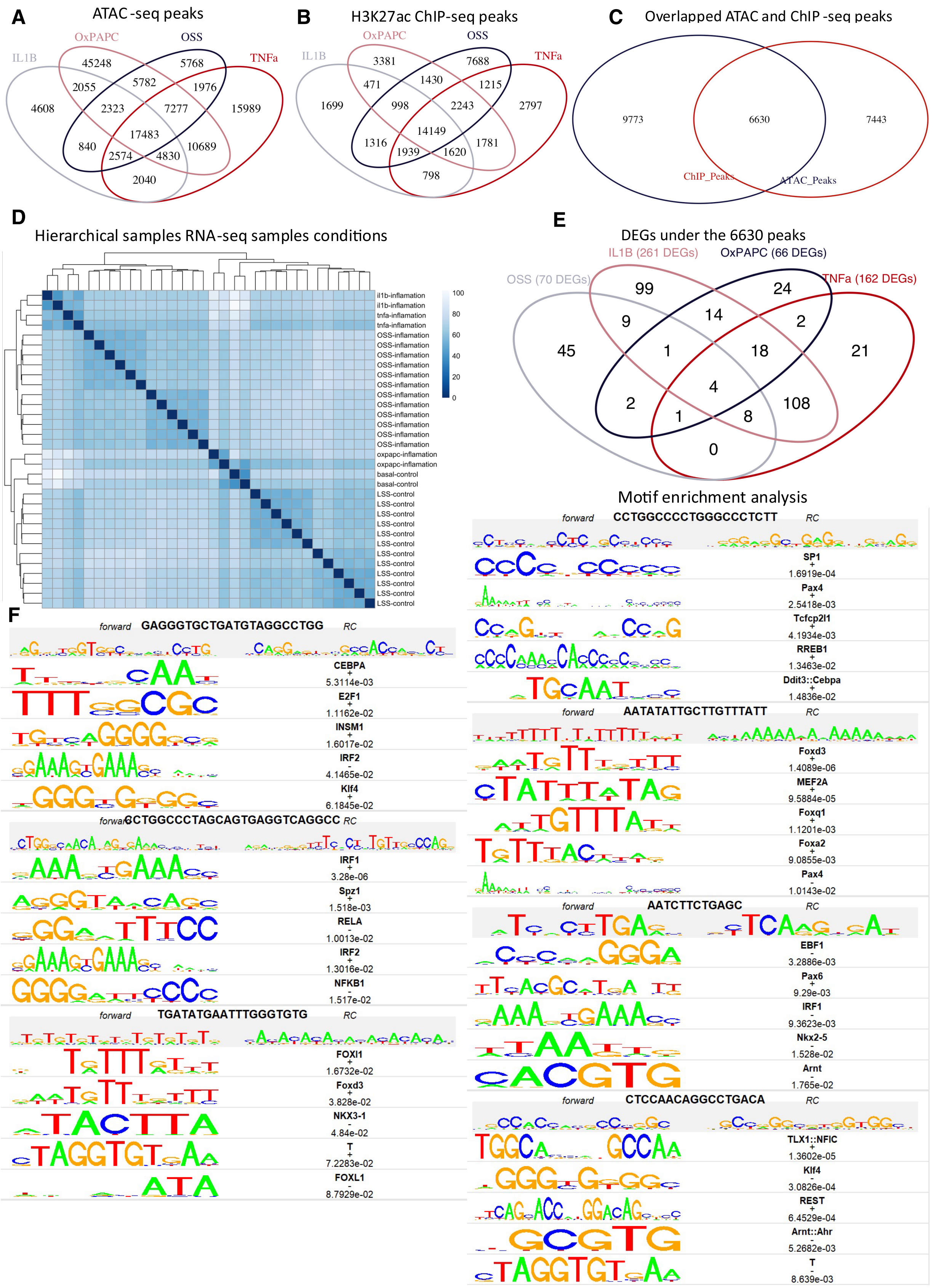
A
ATAC -seq peaks
IL1B
OxPAPC
OSS
TNFa
45248
5768
2055
5782
1976
4608
2323
7277
15989
17483
840
2574
4830
10689
2040
B
H3K27ac ChIP-seq peaks
IL1B
OxPAPC
OSS
TNFa
3381
7688
1430
471
1215
1699
998
2243
2797
14149
1316
1939
1620
1781
798
C
Overlapped ATAC and ChIP -seq peaks
9773
6630
7443
ChIP_Peaks
ATAC_Peaks
D
Hierarchical samples RNA-seq samples conditions
il1b-inflamation
il1b-inflamation
tnfa-inflamation
tnfa-inflamation
OSS-inflamation
oxpapc-inflamation
oxpapc-inflamation
basal-control
basal-control
LSS-control
100
80
60
40
20
0
E
DEGs under the 6630 peaks
IL1B (261 DEGs)
OxPAPC (66 DEGs)
OSS (70 DEGs)
TNFa (162 DEGs)
99
24
9
14
2
45
1
18
21
4
2
1
8
108
0
Motif enrichment analysis
F
forward GAGGGTGCTGATGTAGGCCTGG RC
CEBPA + 5.3114e-03
E2F1 + 1.1162e-02
INSM1 + 1.6017e-02
IRF2 - 4.1465e-02
Klf4 + 6.1845e-02
forward GCTGGCCCTAGCAGTGAGGTCAGGCC RC
IRF1 + 3.28e-06
Spz1 + 1.518e-03
RELA - 1.0013e-02
IRF2 - 1.3016e-02
NFKB1 - 1.517e-02
forward TGATATGAATTTGGGTGTG RC
FOXI1 + 1.6732e-02
Foxd3 + 3.828e-02
NKX3-1 - 4.84e-02
T + 7.2283e-02
FOXL1 - 8.7929e-02
forward CCTGGCCCCTGGGCCCTCTT RC
SP1 + 1.6919e-04
Pax4 + 2.5418e-03
Tcfcp2l1 + 4.1934e-03
RREB1 + 1.3463e-02
Ddit3::Cebpa + 1.4836e-02
forward AATATATTGCTTGTTTATT RC
Foxd3 + 1.4089e-06
MEF2A + 9.5884e-05
Foxq1 + 1.1201e-03
Foxa2 + 9.0855e-03
Pax4 - 1.0143e-02
forward AATCTTCTGAGC RC
EBF1 - 3.2886e-03
Pax6 - 9.29e-03
IRF1 - 9.3623e-03
Nkx2-5 - 1.528e-02
Arnt - 1.765e-02
forward CTCCAACAGGCCTGACA RC
TLX1::NFIC + 1.3602e-05
Klf4 - 3.0826e-04
REST + 6.4529e-04
Arnt::Ahr - 5.2682e-03
T - 8.639e-03

Figure EV1.

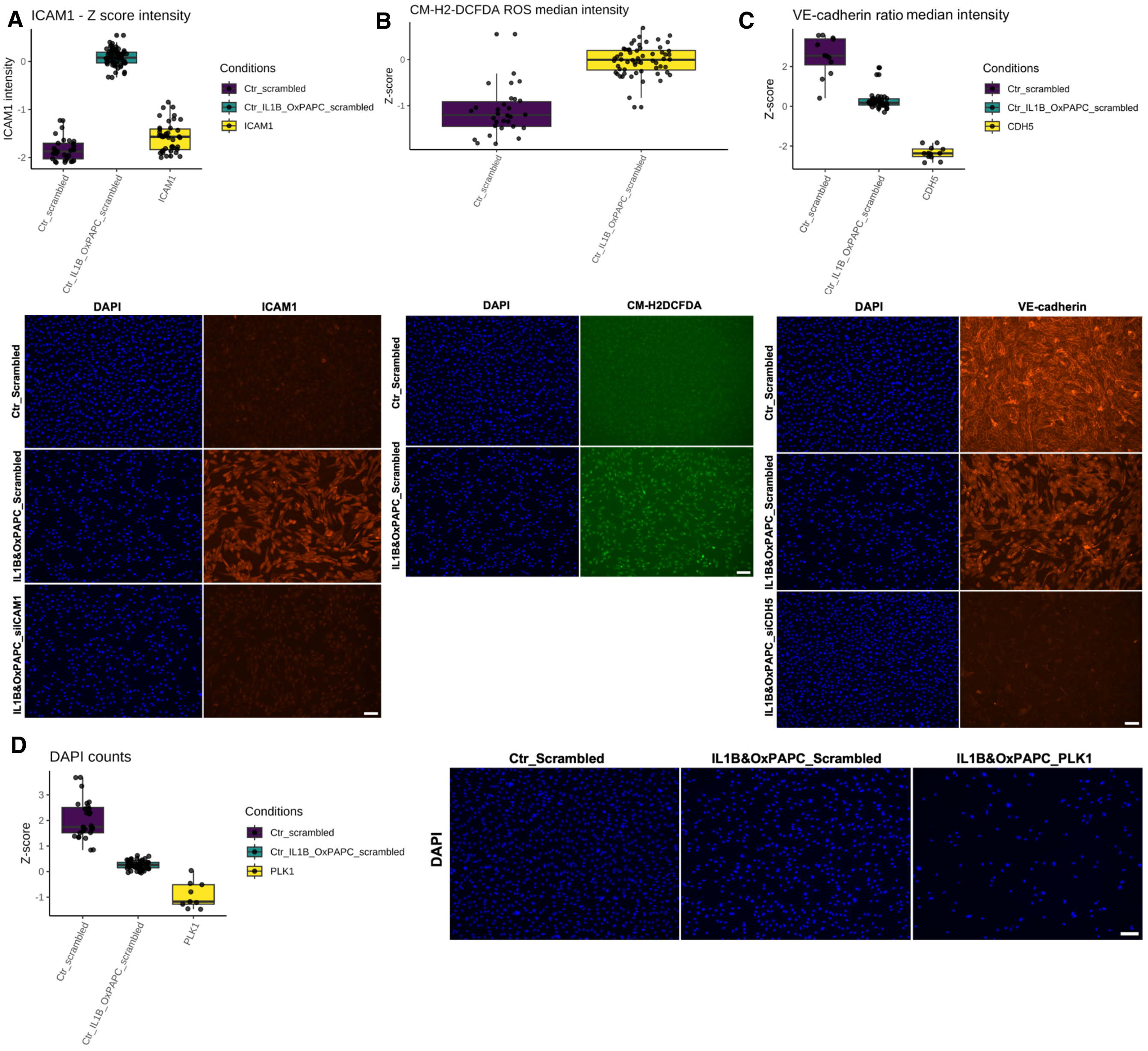

Figure EV2. IL-1β&OxPAPC and siRNA standardisation by immunofluorescence.

A–D Representative fluorescent micrographs (Scale bar = 100 μm) and quantification of (A) ICAM1 intensity, (B) CM-H2DCFDA (ROS indicator) intensity, (C) VE-cadherin intensity and (D) nuclei (DAPI) number in HAEC transfected with Scrambled siRNA or siRNA to ICAM1, CDH5 and PLK1, and treated or not with IL-1β and OxPAPC. The data are represented as a *Z*-score (Materials and Methods). The experiment was repeated 5 times independently (biological replicates), with 4–5 technical replicates from each condition. The representative images of DAPI staining of Ctrl Scrambled in (C) are the same as those used in (A).

Source data are available online for this figure.

 © 2023 The Authors

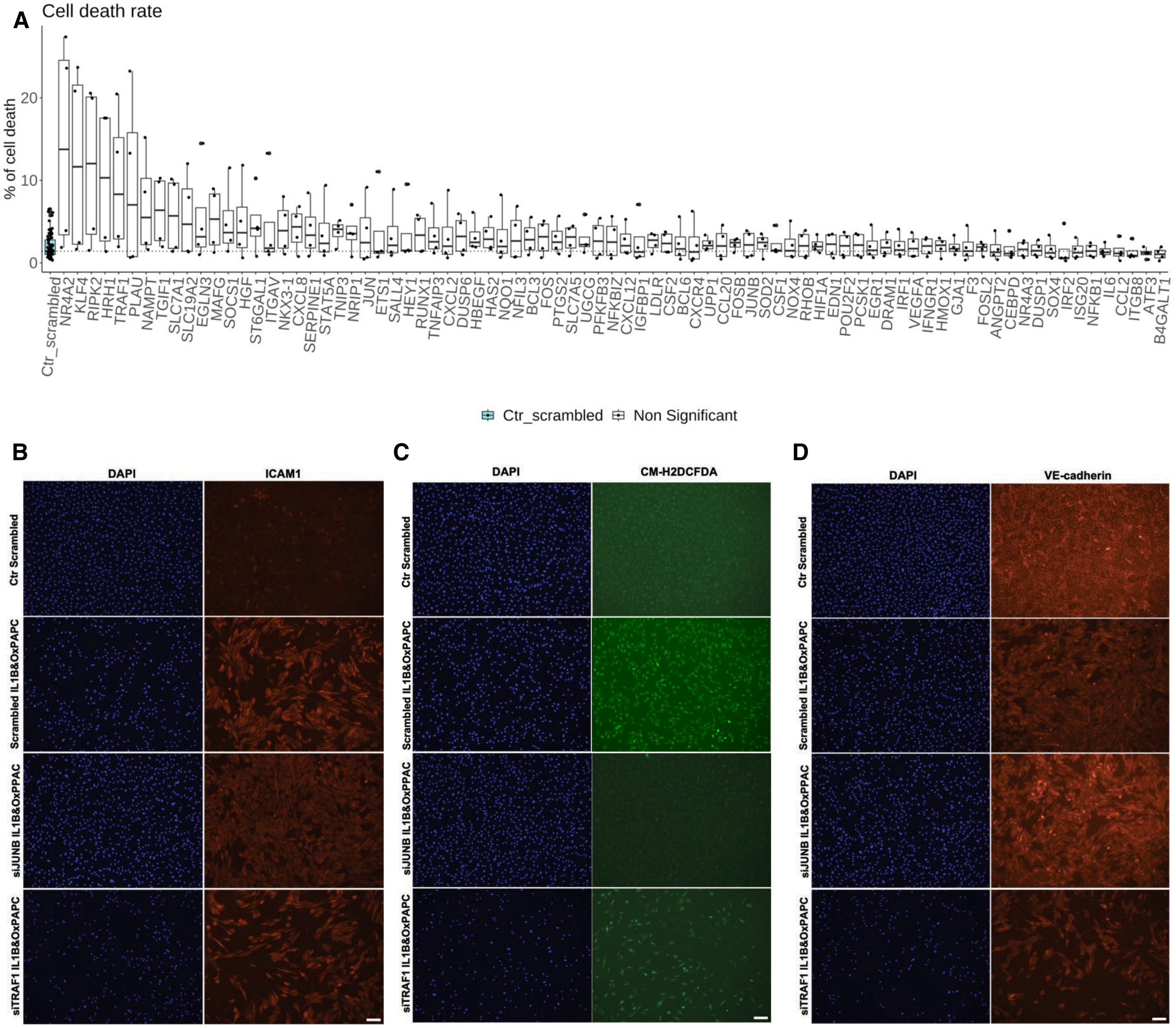

Figure EV3. siRNA transfection and immunofluorescence.

A Percentage of cell death calculated from a cell viability assay (calcein and ethidium staining) in HAEC transfected with Scrambled siRNA or siRNA to 81 genes, without treatment with IL-1β and OxPAPC. *P*-values were computed by multiple pairwise comparisons with the Wilcoxon test followed by BH correction. Data are presented as mean ± standard deviation. The boxplot depicts the median within the 25th and 75th percentiles, which the whisker extends no further than 1.5 × IQR (Interquartile Range). The experiment was repeated 4 times independently (biological replicates), with 8 technical replicates for all controls in the plate each time. All the comparisons were made between HAECs transfected with each of the 81 siRNA versus HAEC transfected with Scrambled siRNA.

B–D Representative fluorescent micrographs (Scale bar = 100 μm) of (A) ICAM1, (B) CM-H2DCFDA (ROS indicator) and (C) VE-cadherin in HAEC transfected with Scrambled siRNA or siRNA to JUNB and TRAF1, and treated or not with IL-1β and OxPAPC. The representative images of DAPI and CM-H2DCFDA staining of Scrambled IL1B&OxPAPC in (C) are the same as those used in Fig EV2B.

Source data are available online for this figure.

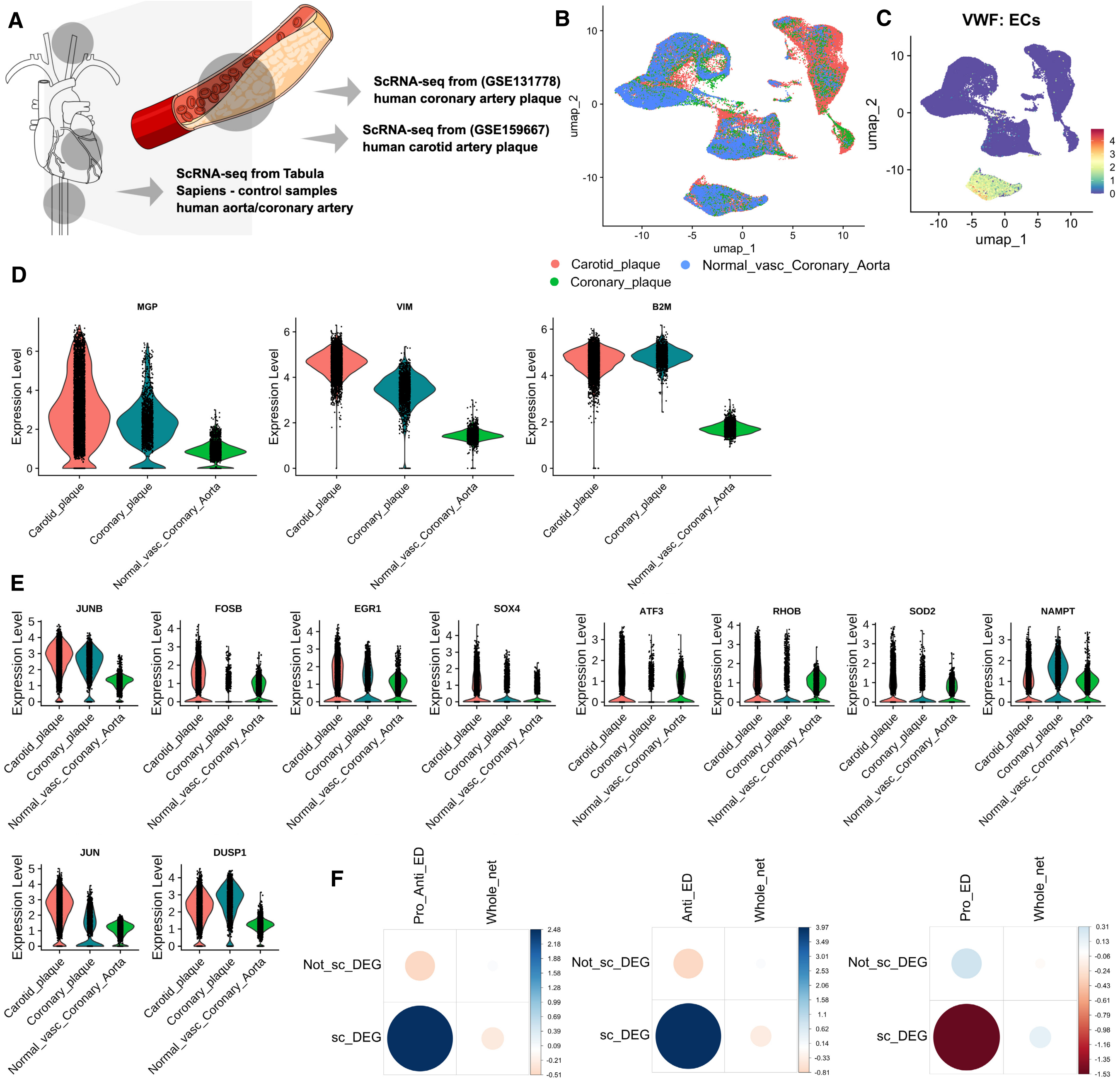

Figure EV4. Validation of the endothelial dysfunction (ED) network with single-cell RNA sequencing (scRNA-seq) datasets obtained from human atherosclerotic plaques.

- A Schematic figure showing the scRNA-seq public data used (Materials and Methods) to validate the ED disease network. The data were composed of two datasets containing atherosclerosis lesions and one control dataset from the Tabula sapiens. The cartoons were created using the Mind the Graph platform (www.mindthegraph.com).
- B Uniform manifold approximation and projection (UMAP) visualisation of the integrated scRNAseq datasets used to validate the ED disease network (n = 69,385 cells).
- C UMAP visualisation of a critical endothelial cell (EC) marker VWF.
- D Violin plot of the three top-upregulated genes in the ECs from atherosclerotic sites compared to the normal vasculature.
- E Violin plot of the TFs differentially expressed *in vitro* (HAECs under the stimuli) and *in vivo* (upregulated in ECs at the atherosclerotic plaque).
- F Hypergeometric test to test enrichment of DEGs from the single cell data in our pro-anti ED network P = 0.002; anti-ED network P = 0.00041; pro-ED network P = 0.171. The plots depict contingency tables for each hypergeometric test. In these tables, values with two decimal places indicate residuals. Positive residuals, suggesting that the observed values were more frequent than expected, are in blue, and negative residuals, suggesting the opposite, are in red.

Source data are available online for this figure.

 © 2023 The Authors

Figure EV5. Network propagation from knockdown genes.

A ED protein–protein interaction network (PIN) disease network generated by combining the 26 pro-ED and 31 anti-ED genes and the random-walk-with-restart (RWR) algorithm. The resulting network comprises 216 nodes and 317 edges. Colour reflects the average log_2 fold change in the gene expression of these genes across the datasets integrated in the study. The size of the node represents the degree, i.e., number of interacting partners. The colour of the outline of the nodes reflects whether it was in the anti- or pro-ED gene list where relevant.

B Distribution of semantic similarity values for all nodes in the pro-ED network and in the anti-ED network.

C Distribution of number of BP terms for the nodes in the anti- and pro-ED networks.

D GO enrichment analysis for Biological Processes for the pro-ED network and its 26 pro-ED seed nodes genes (adjusted *P*-value $\leq$ 0.05)

E GO enrichment analysis for Biological Processes for the anti-ED network and its 31 anti-ED seed nodes genes (adjusted *P*-value $\leq$ 0.05).

Source data are available online for this figure.

© 2023 The Authors

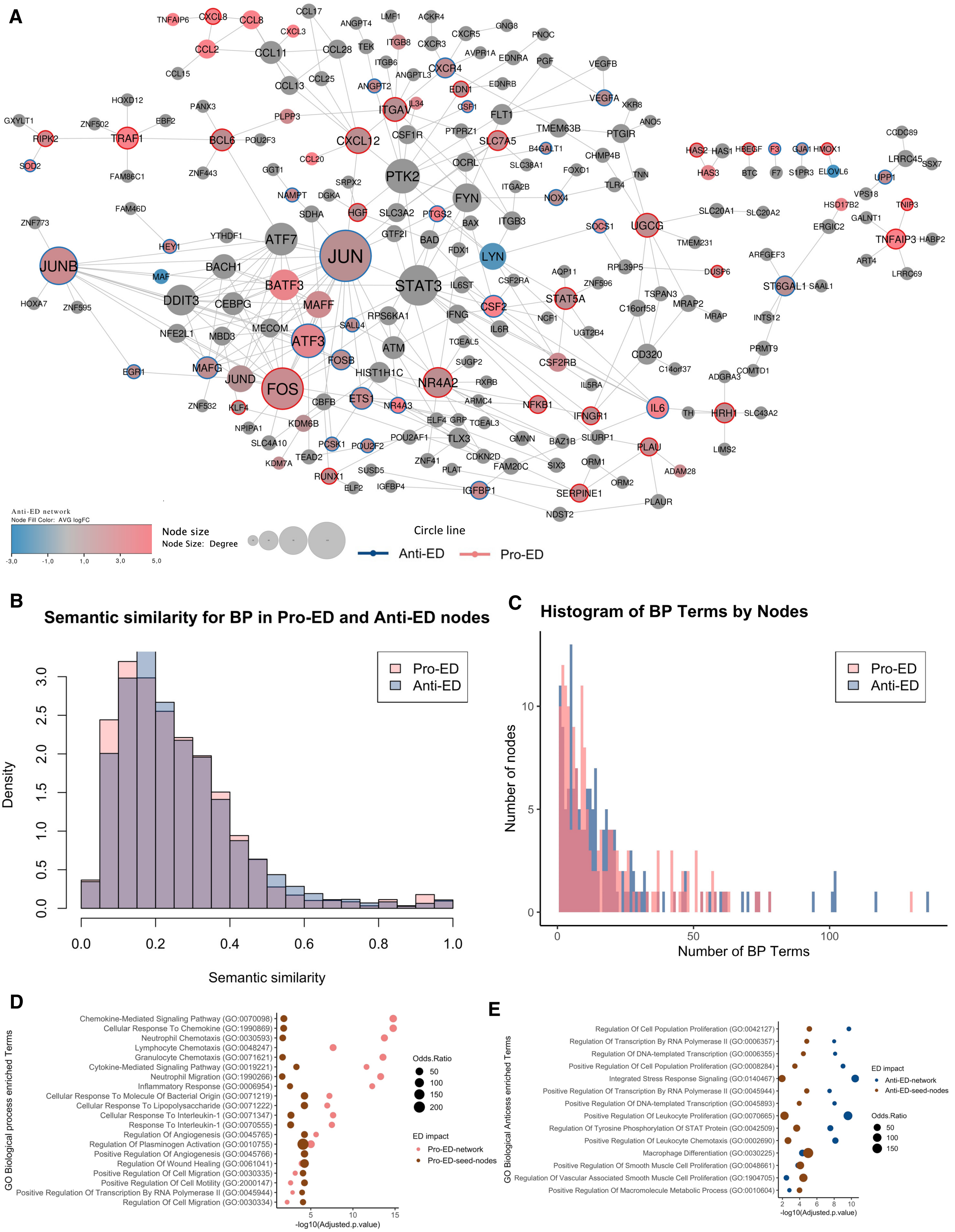

Figure EV5.

 © 2023 The Authors

Figure EV6. Drug treatment high content screen data.

A, B (A) Representative fluorescent micrographs (Scale bar = 50 μm) and (B) quantification of percentage of cell death calculated from a cell viability assay (calcein and ethidium staining) in HAEC treated with the 17 drugs at 10^{-8} and 10^{-6} M, without IL-1β and OxPAPC. Starving treatment for 24 h was used as a cell death positive control. Data are presented as mean ± standard deviation. The experiment was repeated 3 times independently (biological replicates), with 1 technical replicate from each condition. *P*-values were computed by multiple pairwise comparisons with the Wilcoxon test followed by BH correction. The conditions were compared to starving.

C–F Quantification of (C) ICAM1 intensity, (D) CM-H2DCFDA (ROS indicator) intensity, (E) VE-cadherin intensity and, (F) nuclei (DAPI) number in HAEC treated with IL-1β and OxPAPC alone (DMSO solvent), or in combination with inhibitor drugs at 10^{-6} M or 10^{-8} M. The data are represented as a *Z*-score (Materials and Methods). All experiments were repeated 2 times independently (biological replicates), with 4–5 technical replicates from each condition. Only significant results (P-value < 0.05) are shown. The boxplot depicts the median within the 25th and 75th percentiles, which the whisker extends no further than 1.5 × IQR (Interquartile Range).

G Aggregated rank analysis combining the siRNA and drugs screening exacerbating (red) and reducing (blue) ED phenotypes upon treatment. The first ranking, from left to right, rates genes by pro-ED ranking scores (Figs 2F and 4B). These are pro-ED genes. The second ranking is for anti-ED genes, with anti-ED ranking score (Materials and Methods). Both are displayed on the same plot, represented by the 2 dots per target.

Source data are available online for this figure.

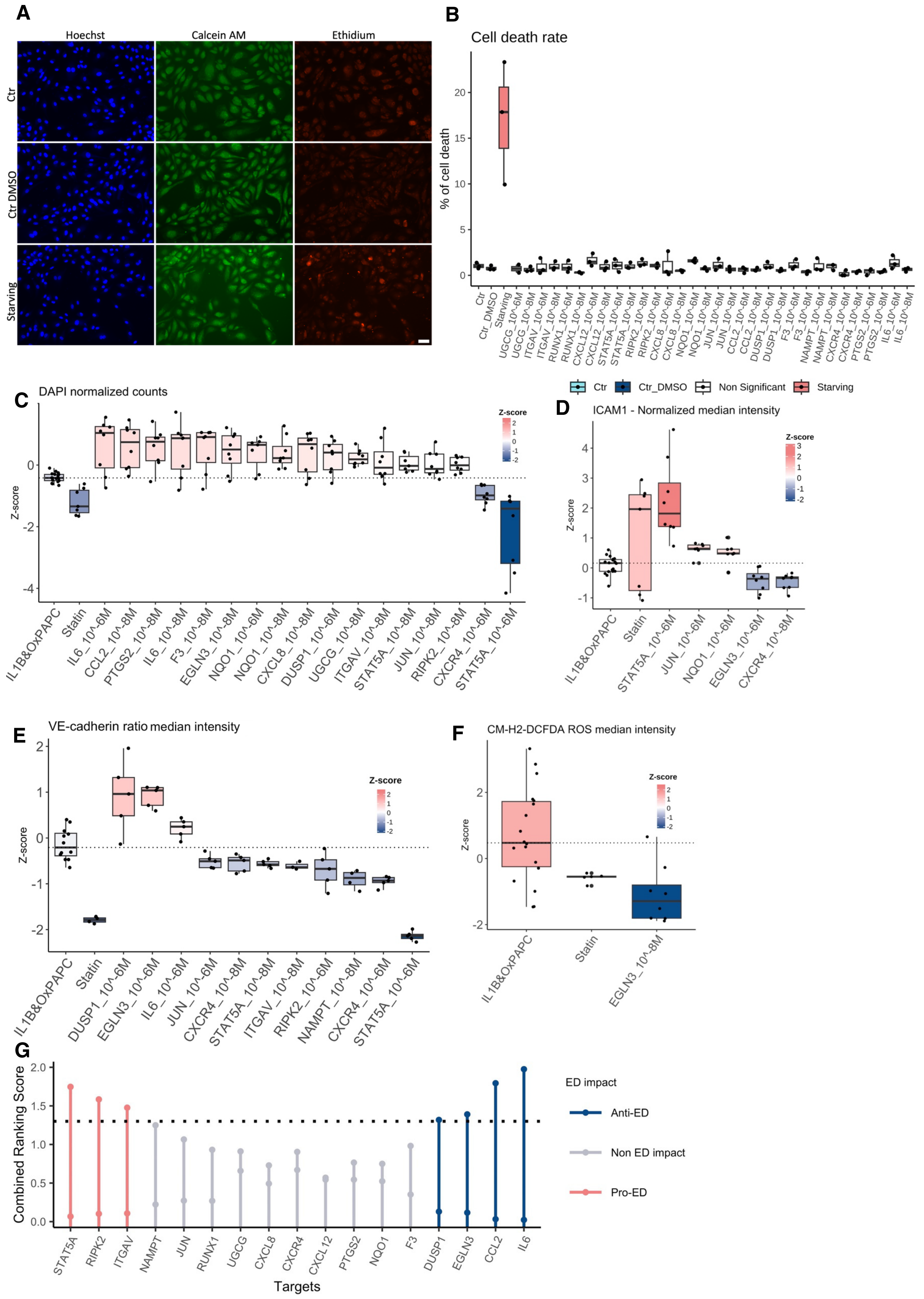

Figure EV6.

© 2023 The Authors
