# Supplementary material for: Integrated systems biology approach identifies gene targets for endothelial dysfunction
Source: Mol Syst Biol. 2023 Nov 30;19(12):e11462. doi: 10.15252/msb.202211462 (PMC10698507; doi:10.15252/msb.202211462)
Supplement: Supplementary file 5 — Dataset EV2 [file MSB-19-e11462-s016.zip › Dataset_EV1/README.rtf]

Quantification of Raw Images for ICAM1, ROS, and Ve-Cadherin Markers This repository contains tables that present the quantification results obtained from the raw image analysis for the markers ICAM1, ROS, VE-cadherin, nuclei counts, and live/dead assay (See methods). Every two plates should be considered as one biological replicate for all the 81 siRNA genes and EGLN family. There is also a table with maps for all genes and controls within each plate. 
