# Supplementary material for: Integrated systems biology approach identifies gene targets for endothelial dysfunction
Source: Mol Syst Biol. 2023 Nov 30;19(12):e11462. doi: 10.15252/msb.202211462 (PMC10698507; doi:10.15252/msb.202211462)
Supplement: Supplementary file 6 — Dataset EV3 [file MSB-19-e11462-s001.zip › Dataset_EV3/README.rtf]

Network tables originated from RWR (Methods)This repository contains tables that present the pro-ED, anti-ED and they combined.
