# Supplementary material for: Integrated systems biology approach identifies gene targets for endothelial dysfunction
Source: Mol Syst Biol. 2023 Nov 30;19(12):e11462. doi: 10.15252/msb.202211462 (PMC10698507; doi:10.15252/msb.202211462)
Supplement: Supplementary file 8 — Dataset EV5 [file MSB-19-e11462-s007.zip › Dataset_EV5/README.rtf]

Table with all drugs/diseases available for the nodes of pro-ED and Anti-ED networks (Methods)This repository contains tables that present the pro-ED, anti-ED nodes that present drugs and the disease they are targeting.This repository contains tables that present the pro-ED, anti-ED nodes that present drugs for heart related disease they are targeting.
