# Supplementary material for: Integrated systems biology approach identifies gene targets for endothelial dysfunction
Source: Mol Syst Biol. 2023 Nov 30;19(12):e11462. doi: 10.15252/msb.202211462 (PMC10698507; doi:10.15252/msb.202211462)
Supplement: Supplementary file 11 — PDF+ [file MSB-19-e11462-s012.pdf]

Article

SOURCE DATA | TRANSPARENT PROCESS | OPEN ACCESS

molecular systems biology

Integrated systems biology approach identifies gene targets for endothelial dysfunction

Iguaracy Pinheiro-de-Sousa[1,2,†], Miriam Helena Fonseca-Alaniz[1,†], Girolamo Giudice[2], Iuri Cordeiro Valadão[1], Silvestre Massimo Modestia[1], Sarah Viana Mattioli[1,3], Ricardo Rosa Junior[1], Lykourgos-Panagiotis Zalmas[4,5], Yun Fang[6], Evangelia Petsalaki[2,*] & José Eduardo Krieger[1,**]

Abstract

Endothelial dysfunction (ED) is critical in the development and progression of cardiovascular (CV) disorders, yet effective therapeutic targets for ED remain elusive due to limited understanding of its underlying molecular mechanisms. To address this gap, we employed a systems biology approach to identify potential targets for ED. Our study combined multi omics data integration, with siRNA screening, high content imaging and network analysis to prioritise key ED genes and identify a pro- and anti-ED network. We found 26 genes that, upon silencing, exacerbated the ED phenotypes tested, and network propagation identified a pro-ED network enriched in functions associated with inflammatory responses. Conversely, 31 genes ameliorated ED phenotypes, pointing to potential ED targets, and the respective anti-ED network was enriched in hypoxia, angiogenesis and cancer-related processes. An independent screen with 17 drugs found general agreement with the trends from our siRNA screen and further highlighted *DUSP1*, *IL6* and *CCL2* as potential candidates for targeting ED. Overall, our results demonstrate the potential of integrated system biology approaches in discovering disease-specific candidate drug targets for endothelial dysfunction.

Keywords data integration; drug targets; endothelial dysfunction; network analysis; systems biology

Subject Categories Cardiovascular System; Molecular Biology of Disease; Pharmacology & Drug Discovery

DOI 10.15252/msb.202211462 | Received 18 November 2022 | Revised 20 October 2023 | Accepted 23 October 2023

Mol Syst Biol. (2023) 19: e11462

Introduction

The endothelium is a single-layer lining of blood and lymphatic vessels (Lüscher & Barton, 1997; Boulanger, 2016) that plays a crucial role in maintaining homeostasis. It regulates vascular tone and angiogenesis while also promoting an antioxidant, anti-inflammatory and anti-thrombogenic response (Godo & Shimokawa, 2017; Fang *et al*, 2019). Distributed throughout the body, the endothelium is exposed to various pressure and flow patterns. Regions of arterial branching and curvature, which are prone to atherosclerotic plaque development (Peiffer *et al*, 2013; Souilhol *et al*, 2020; Liu *et al*, 2021), experience disturbed shear forces and show early signs of endothelial dysfunction (ED) (Mudau *et al*, 2012; Peiffer *et al*, 2013; Liu *et al*, 2021). These disturbed shear forces, combined with cardiovascular risk factors like high cholesterol, trigger chronic inflammation and accelerate ED and atherosclerosis (Steinberg & Witztum, 2010; de Vries & Quax, 2016; Gencer *et al*, 2021).

Traditionally, ED was defined as a reduced capacity to regulate vascular tone (reduced nitric oxide (NO) production). However, its definition has recently expanded to include increased oxidative stress, chronic inflammation, compromised barrier integrity, and impaired injury repair, among other functions (Xu *et al*, 2021). ED is associated with most cardiovascular diseases (CVDs) and serves as a prognostic biomarker for future cardiovascular events like myocardial infarction or stroke (Deanfield *et al*, 2007; Mudau *et al*, 2012; Cyr *et al*, 2020). Given the multifunctional role of endothelial cells (ECs) and their clinical relevance to various CVDs, ED has emerged as a potential therapeutic target for several vascular disorders (Daiber *et al*, 2017; Huynh & Heo, 2019; Premer *et al*, 2019). However, despite its importance to human health, the underlying causal mechanisms of ED remain unknown, hindering the development of therapies directly targeting ED.

1 Laboratory of Genetics and Molecular Cardiology, Heart Institute (InCor)/University of São Paulo Medical School, São Paulo, Brazil
2 European Molecular Biology Laboratory, European Bioinformatics Institute, Hinxton, UK
3 Department of Biophysics and Pharmacology, Institute of Biosciences of Botucatu, Universidade Estadual Paulista, Botucatu, Brazil
4 Wellcome Trust Sanger Institute, Wellcome Trust Genome Campus, Cambridge, UK
5 Open Targets, Wellcome Genome Campus, Cambridge, UK
6 Department of Medicine, University of Chicago, Chicago, IL, USA
*Corresponding author. Tel: +44 1223 492665; E-mail: petsalaki@ebi.ac.uk
**Corresponding author. Tel: +55 11 2661 5022; E-mail: j.krieger@hc.fm.usp.br
†These authors contributed equally to this work

© 2023 The Authors. Published under the terms of the CC BY 4.0 license

Endothelial dysfunction is typically associated with multiple cardiovascular risk factors and genetic susceptibility rather than single perturbations (Daiber & Chlopicki, 2020). Our recent research (Pinheiro-de-Sousa *et al*, 2022) has revealed that these cardiovascular risk factors can influence endothelial gene expression in a hierarchical manner. Interestingly, when these factors are clustered together, the combined effect of individual risk factors only explains approximately 56% of the response, indicating the existence of emergent properties that arise from their interactions (Pinheiro-de-Sousa *et al*, 2022). While our previous study explored the interactions of different stimuli, it lacked a comprehensive understanding that other omics layers can provide. The epigenome, in particular, integrates signalling and genomic information to regulate gene expression and cellular behaviour. Therefore, to gain a more comprehensive understanding of ED, we reasoned that a systems biology approach that considers multiple perturbations and incorporates the epigenomic and gene regulatory layers could provide key insights. To this end, here we combined epigenomic with transcriptomic data integration, siRNA and drug screening with network inference to identify network signatures promoting and protecting against ED when perturbed, as well as potential gene targets and drugs for repurposing to treat ED.

Results

Mimics of cardiovascular risk factors OSS, IL-1β, TNF-α and OxPAPC have common effects on the epigenetic landscape and transcriptional programme

We first set out to identify gene regulatory regions specific to ED to prioritise ED genes of high putative relevance. ED is the common result of multiple diverse pressures (Daiber & Chlopicki, 2020; Pinheiro-de-Sousa *et al*, 2022), which can be experimentally modelled through exposure of endothelial cells to multiple stimuli that serve as mimics for cardiovascular disease factors. We hypothesised that integrating epigenetic and gene expression datasets from these studies would identify common ED-specific regions. To achieve this, we re-analysed publicly available ChIP-seq, ATAC-seq and RNA-seq data from human aortic endothelial cells (HAECs) exposed to various stimuli, including mechanical stimuli such as laminar shear stress (LSS) and oscillatory shear stress (OSS), as well as chemical stimuli such as the inflammatory cytokines IL-1β, TNF-α, and oxidised phospholipids (OxPAPC) (Materials and Methods) (Hogan *et al*, 2017; Krause *et al*, 2018). We focused on transcription factor (TF) activities that were commonly identified by all conditions in the epigenetic data and then filtered the differentially expressed genes (DEGs) to include only those that were targets of the identified TFs.

Our analysis revealed 17,483 open chromatin regions that were common among HAECs subjected to OSS, IL-1β, TNF-α and OxPAPC, as measured by ATAC-seq analysis (Figs 1A and EV1A). Additionally, we identified 14,149 enhancer-like elements marked by significant levels of acetylation of lysine 27 (H3K27ac) that were common among the different stimuli (Figs 1A and EV1B), as identified by ChIP-seq analysis. By using ATAC-seq peaks to define the centre of the enhancer-like regions (ChIP-seq peaks), we identified 6,630 high-confidence regulatory regions that were commonly modulated by all stimuli (Figs 1A and EV1C). Notably, most of these peaks (~60%) were found in promoter regions (Fig 1B).

We then performed differential expression analysis to identify differentially expressed genes (DEGs) among the different stimuli. Principal component analysis and hierarchical clustering revealed two major groups of stimuli: one group including IL-1β, TNF-α and OSS, and the other group including LSS, static basal control, and OxPAPC (Figs 1C and EV1D). For our analysis, we compared OSS to LSS instead of static controls due to data availability, whereas IL-1β, TNF-α and OxPAPC treatments were compared to their respective static controls. We identified 578 DEGs (OSS), 928 DEGs (IL-1β), 594 DEGs (TNF-α), and 186 DEGs (OxPAPC) in the respective cardiovascular risk factor models. Among these, we selected only those genes that were annotated to the 6,630 identified peaks, resulting in a set of 356 DEGs (Fig EV1E). These genes were found to be enriched in known processes associated with ED, including inflammatory pathways (e.g., cytokine production, cell-adhesion molecules and leukocyte chemotaxis), oxidative stress and regulation of angiogenesis and vasculature development (Fig 1D).

To further improve our confidence in our identified ED genes, we filtered the 356 DEGs identified above, keeping only those regulated by TFs predicted to bind the identified peaks from the epigenetic data. Specifically, we identified key TFs that were relevant to the stimuli and their downstream DEGs by performing *de novo* motif

Figure 1. Common regulatory regions on the genome are modulated by mimics of cardiovascular risk factors OSS, IL-1β, TNF-α and OxPAPC. ▶

A Heatmap representing the common regulatory regions to all stimuli: 17,267 common accessible chromatin regions identified through ATAC-seq analysis. 14,149 common enhancer-like regions marked by H3K27ac (ChIP-seq), and additionally, the overlapped 6,630 ATAC and ChIP peaks within a distance of 3 kb from promoters are shown.

B Distribution of the genomic features of the 6,630 common enhancer-like regions. The genomic features include whether a peak is in the TSS, Exon, 5′ UTR, 3′ UTR, intronic, or intergenic.

C Principal component analysis of gene expression profiles from each sample under IL-1β, TNF-a and OSS demonstrates the major components PC1 of the variance to separate samples from basal control, OxPAPC, and LSS. PC1 and PC2 accounted for 45% and 37% of the total variance, respectively.

D Gene Ontology (GO) enrichment analysis was performed for biological processes (BP) using a significance threshold of adjusted $P \leq 0.05$. 356 differentially expressed genes (DEGs) meeting the criteria of adjusted $P \leq 0.05$ and $|\log_2 \text{Fold change}| \geq 1.5$ were considered for the GO enrichment analysis. The analysis was conducted on the DEGs identified under the 6,630 peaks.

E The top four enriched motifs, located at the centre of enhancer-like regions, are presented along with the corresponding transcription factor family, motif sequence and enrichment log *P*-values. Enrichment was calculated based on a 200 bp sequence centred on chromatin accessibility.

F A Sankey diagram is provided to illustrate the interactions between transcription factors (TFs) and DEGs. The diagram visualises the regulatory relationships between TFs and downstream DEGs demonstrating the network of interactions.

Source data are available online for this figure.

 © 2023 The Authors

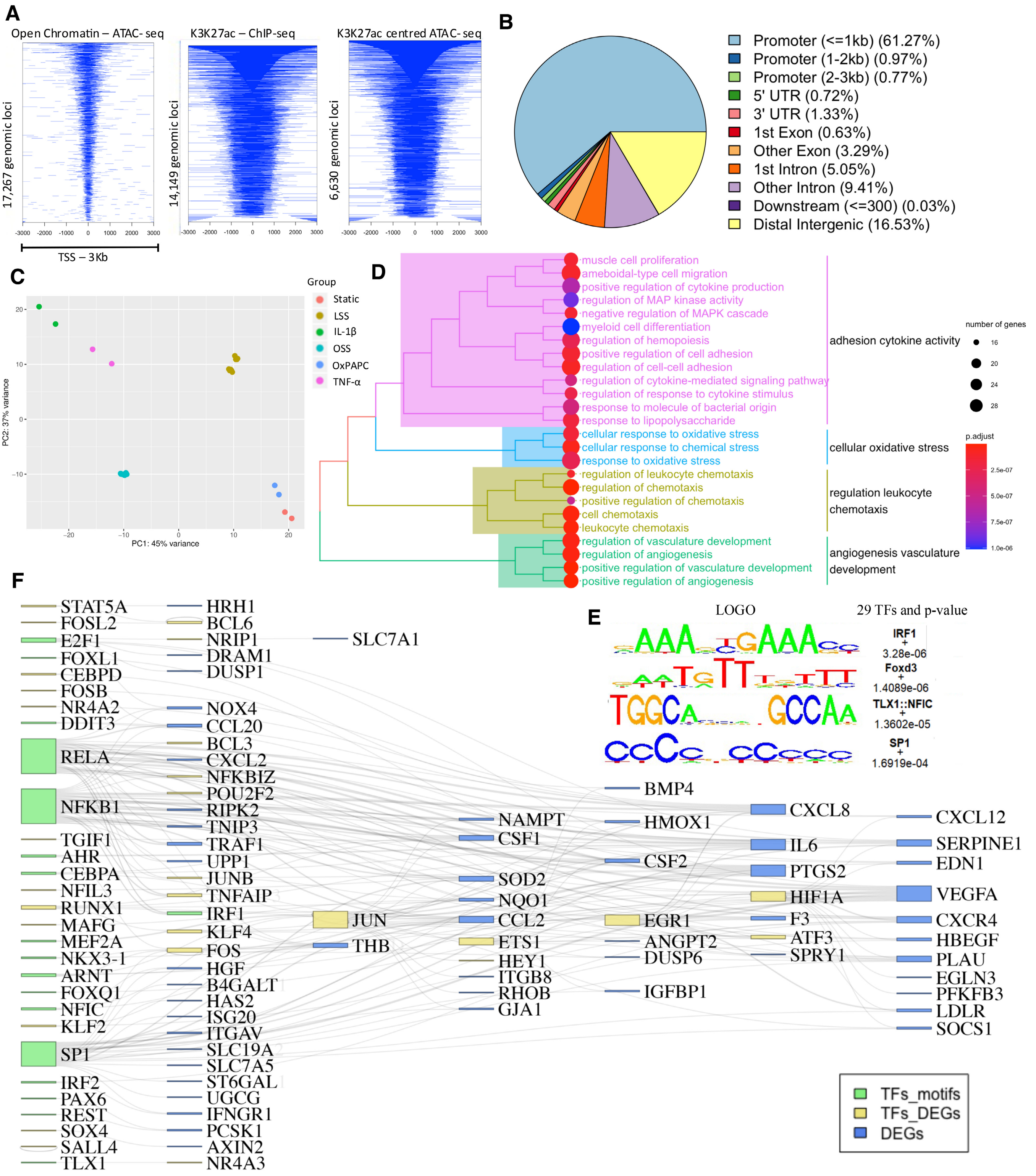

Figure 1.

enrichment analysis at the 6,630 identified peaks. We discovered seven significantly enriched motifs and predicted 29 TFs that were likely to bind to these motifs ($P < 0.05$, Fig EV1F; Materials and Methods). The most significant TFs identified were Foxd3 ($P = 3.3e\text{-}06$), IRF1 ($P = 1.4e\text{-}06$), TLX1: NFIC ($P = 1.4e\text{-}05$), and SP1 ($P = 1.6919e\text{-}06$) (Fig 1E). We then considered only the TF-DEG interactions supported by the TRRUST database and present at the 6,630 enhancer-like regions (Han *et al*, 2018). This resulted in

the identification of 100 genes, including 46 TFs and 54 non-TFs (Fig 1F and Dataset EV1). Notably, NFKB1 and RELA, which are associated with the inflammatory response (Mussbacher *et al*, 2019), were found to regulate the largest number of downstream DEGs. Furthermore, NFKB1 was upregulated in the IL-1β and TNF-α conditions compared to their basal controls.

Overall, our analysis provides a set of ED genes that serve as the foundation for constructing ED disease networks and identifying key node genes that have the potential to be therapeutic targets for cardiovascular diseases. Out of the 100 identified ED genes, 81 were upregulated, 5 were downregulated, and 14, all of which were TFs enriched in the motifs found in the overlapping peaks, were not found to be significantly differentially expressed. Subsequent analyses in this study focused on the 81 upregulated genes, to identify potential targets that can be inhibited to gain insights on the gene rewiring adaptation associated with ED and to treat ED.

In vitro siRNA screen finds 83% of ED genes to have an effect on an ED phenotype

We next induced ED by co-stimulating endothelial cells (ECs) with IL-1β and OxPAPC (Materials and Methods) and investigated the impact of 81 upregulated genes on three well-known surrogate markers associated with ED: increased inflammation, ROS production, cell permeability and cell viability (Fig EV2). Inflammation and ROS production play crucial roles in the initiation and progression of ED (Xu *et al*, 2021). Inflamed ECs express various key molecules, including chemokines, interferons and inflammatory markers like intercellular adhesion molecule-1 (ICAM1), which contribute to monocyte adhesion and plaque growth (Xu *et al*, 2021). Additionally, the loss of endothelial barrier integrity and increased permeability have been associated with reduced VE-cadherin membrane-localised expression (Chan *et al*, 2020).

We performed a siRNA screen using high-content imaging in HAECs stimulated with IL-1β (10 ng/ml) and OxPAPC (50 μg/ml) to induce the ED phenotype (Birukov *et al*, 2004; Du *et al*, 2015), and quantified the changes in nuclei counts by DAPI staining, as well as essential readouts for ED, namely general ROS levels, ICAM1 and VE-cadherin protein expression (Figs 2A–E and EV3; Dataset EV2; Materials and Methods).

Nuclei counts demonstrate that several genes are essential for the ECs survival under the IL-1β & OxPAPC pathological stimuli (Fig 2A and B). Knockdown of 52 genes resulted in significant changes in nuclei counts, with 47 increasing cell death (top genes were *NRA42*, *FOS*, *TRAF1*, *HGF* and *SLC7A5*), and 5 decreasing it (*JUNB*, *SALL4*, *NOX4*, *VEGFA* and *FOSB*). To investigate whether the increased cell death associated with some genes was dependent on the pathological chemical stimuli, we performed a cell viability assay (calcein and ethidium staining) for HAEC transfected with each of the 81 siRNA without the IL-1β and OxPAPC stimuli. None of the genes appeared to be essential in the absence of the pathological stimuli, suggesting their specific relevance to cell survival and function in the context of endothelial dysfunction (Fig EV3A).

Knockdown of 38 genes significantly modulated ICAM1 protein expression, with 25 genes increasing ICAM1 staining (top hits *HGF*, *FOS*, *SLC7A5*, *NR4A2* and *TRAF1*), while 13 genes decreased ICAM1 expression (top hits *CSF2*, *NR4A3*, *ST6GAL1*, *UPP1* and *VEGFA*) compared to our control cells transfected with scrambled siRNA in the same conditions (Figs 2A and C, and EV3B). Knockdown of 23 genes modulated ROS production, among which 13 increased ROS production (top hits were *TRAF1*, *SLC7A5*, *FOS*, *HGF* and *NR4A2*), and 10 had an antioxidant effect (top genes were *JUNB*, *HEY1*, *SOCS1*, *NR4A3* and *MAFG*) (Figs 2A and D, and EV3C). Knockdown of 21 genes significantly modulated VE-cadherin membrane staining, with 20 of them decreasing its expression (top hits were *TRAF1*, *SLC7A5*, *FOS*, *HGF* and *NR4A2*), and one gene increasing VE-cadherin staining (*LDLR*) (Figs 2A and E, and EV3D). In summary, 68 of the 81 genes had a significant impact on at least one of the ED readouts when their gene expression was silenced.

Next, to integrate the results of our experiments, we performed an aggregated rank analysis (Kolde *et al*, 2012) using the *Z*-score values of their readouts for each gene (Fig 2F; Materials and Methods). By combining the four readouts, ICAM1, ROS, VE-cadherin and nuclei counts (cell death), we generated two ranked lists: the "pro-endothelial dysfunction (pro-ED)" list includes 26 genes that when knocked down significantly exacerbate the ED phenotypes tested (Fig 2F), with *TRAF1*, *FOS*, *HGF*, *SLC75A* and *NR4A2* being the top hits. The "anti-endothelial dysfunction (anti-ED)" list consists of 31 genes that significantly ameliorate the ED phenotypes (Fig 2F), with *JUNB*, *NR4A3*, *SALL4*, *CSF2* and *HEY1* being the top hits.

Network propagation reveals an ED network that is enriched in DEGs from human ECs located at human atherosclerotic plaques

The pro- and anti-ED genes that we identified provide putative targets and genes that can be important for endothelial cells adaptation

Figure 2. siRNA library high content screen data.

A Heatmap summarising the outcome of the immunofluorescence assays for ICAM1 expression (antibody anti-ICAM1), ROS intensity (probe CM-H2DCFDA), VE-cadherin expression (antibody anti-VE-cadherin) and nuclei counts (DAPI). Colour reflects the median *Z*-score from all replicates.

B–E Quantification of (B) nuclei (DAPI) counts, (C) ICAM1 intensity, (D) ROS (probe CM-H2DCFDA) intensity and (E) VE-cadherin intensity in HAEC transfected with Scrambled siRNA or siRNA to 81 target genes, and treated with IL-1β and OxPAPC. The data are represented as a *Z*-score (Materials and Methods). All experiments were repeated 5 times independently (biological replicates), with 8 technical replicates for all controls in the plate each time. *P*-values were computed by multiple pairwise comparisons with the Wilcoxon test followed by BH correction. The comparisons were made between HAECs transfected with each of the 81 siRNA versus HAEC transfected with Scrambled siRNA and treated with IL-1β and OxPAPC. Bootstrapping analysis was used to mitigate the unbalanced Scrambled_IL1B&OxPAPC control versus the 81 siRNA-targeted genes (Materials and Methods). The boxplot depicts the median within the 25th and 75th percentiles, which the whisker extends no further than 1.5 × IQR (Interquartile Range).

F Aggregated rank analysis for genes exacerbating (red) and ameliorating (blue) endothelial dysfunction (ED) phenotypes upon knockdown. The first ranking, from left to right, rates genes by *Z*-scores, with increased ICAM1 and ROS and decreased DAPI and VE-cadherin. These are pro-ED genes. The second ranking is for anti-ED genes, with opposite criteria (Materials and Methods). Both are displayed on the same plot, represented by the 2 dots per gene.

Source data are available online for this figure.

 © 2023 The Authors

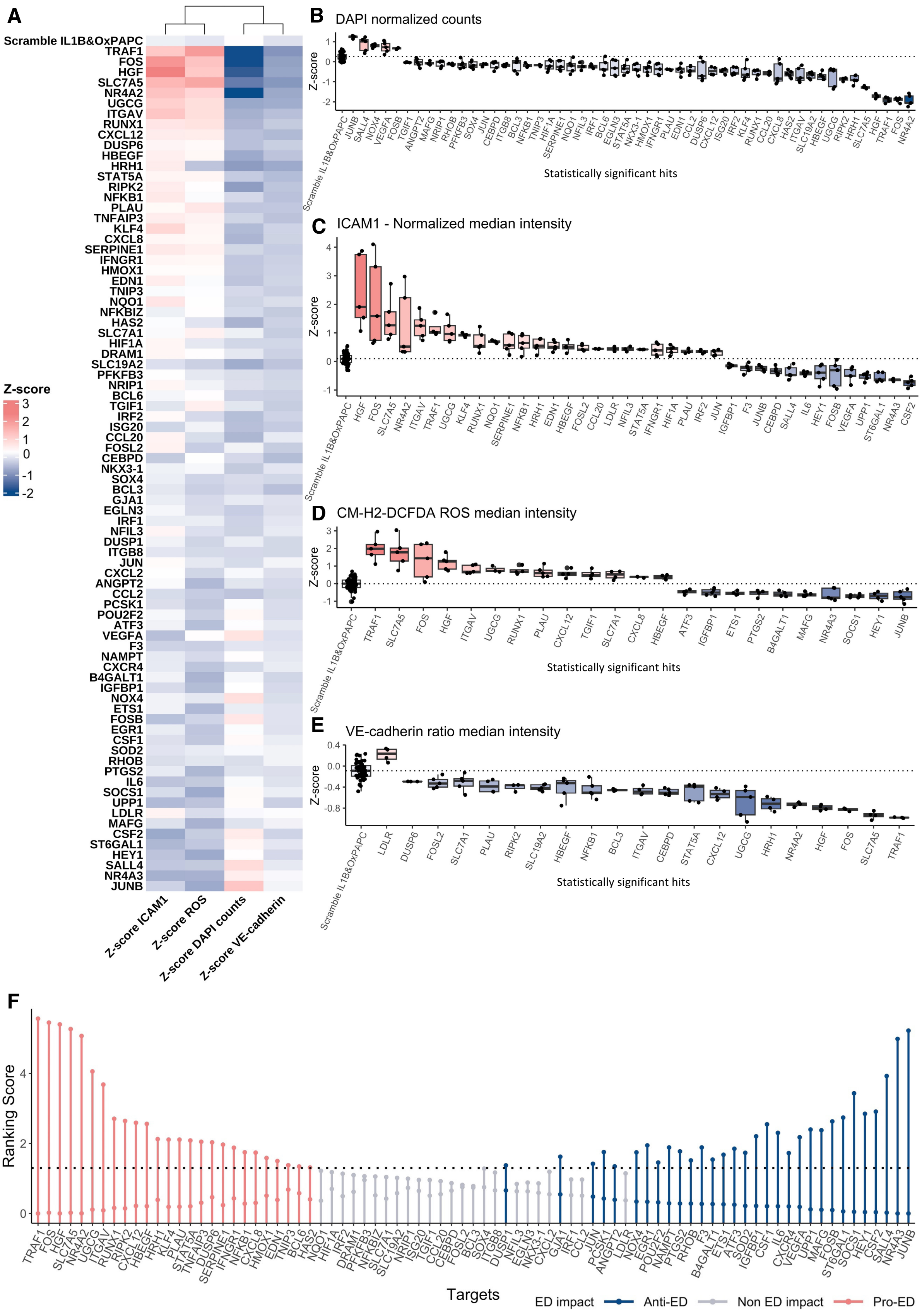
A
B
DAPI normalized counts
Z-score
Statistically significant hits
C
ICAM1 - Normalized median intensity
D
CM-H2-DCFDA ROS median intensity
E
VE-cadherin ratio median intensity
Z-score
Z-score ICAM1
Z-score ROS
Z-score DAPI counts
Z-score VE-cadherin
Scramble IL1B&OxPAPC
F
Ranking Score
Targets
ED impact
Anti-ED
Non ED impact
Pro-ED

Figure 2.

to the pathological stimuli. These lists of genes, however, do not give us information regarding the relationships between them or their functional context. To gain better insights into the processes that are associated with cell survival during ED and the disease phenotype, we employed a network analysis strategy. Specifically, we used the respective 26 and 31 pro- and anti-ED knockdown genes and the random-walk-with-restart (RWR) algorithm (Tong *et al*, 2008) on a human protein interaction network (Kerrien *et al*, 2012; Hornbeck *et al*, 2015; Türei *et al*, 2016; Surdo *et al*, 2017) to generate network signatures associated with these two EC phenotypes (Materials and Methods). The resulting pro- and anti- ED, and combined ED network signatures comprise 176, 167, and 216 nodes and 224, 268 and 317 edges, respectively (Figs 3A and B, and EV5A; Dataset EV3).

To validate the relevance of our combined ED network in an *in vivo* context, we re-analysed three single-cell RNA sequencing (scRNA-seq) datasets obtained from human atherosclerotic vasculature (Wirka *et al*, 2019a; Alsaigh *et al*, 2022a; Jones *et al*, 2022a) (Fig EV4A). Specifically, we compared two datasets obtained from atherosclerotic plaques to those derived from vasculature without plaques (Fig EV4B). ECs were further categorised (Fig EV4C), and differential expression analysis between ECs from plaques and normal vasculature identified 705 DEGs (526 upregulated and 179 downregulated, Dataset EV4). Among the 526 upregulated DEGs, were several well-established genes associated with atherosclerosis plaques, such as matrix Gla protein (Herrmann *et al*, 2000), VIM (Shi *et al*, 2021), and B2M (Shi *et al*, 2021) (Fig EV4D). We found that 14 out of our 81 ED genes were also upregulated in ECs derived from atherosclerotic plaque *in vivo* samples, with 10 of them (Fig EV4E) from the anti-ED group, including *RHOB*, *SOD2*, *EGR1* and *JUNB* (Fig 3B and Dataset EV4).

We found that the DEGs within ECs from plaques were significantly enriched within our combined and anti-ED gene networks ($P < 0.02$ and $P < 0.0005$ respectively) but not our pro-ED gene network ($P = 0.171$) when compared to the background human protein interaction network, as determined by the hypergeometric test (Fig EV4F). This enrichment supports the relevance of our ED disease gene networks in the *in vivo* condition, particularly within the context of atherosclerotic vasculature, and our hypothesis that the pro-ED network represents network adaptation rather than being causal for the disease phenotype.

Looking at the combined ED network (Fig EV5A) we find that the genes form a relatively interconnected network indicating that there is crosstalk between the processes related to cell adaptation and survival in ED and those associated causally with the disease phenotypes.

Next, we compared the pro- and anti-ED networks to better understand their respective properties. In terms of network topology, the pro-ED network has a lower clustering coefficient of 0.058, suggesting that the network rewiring associated with survival and adaptation in ED conditions is less interconnected and includes more distinct sub-clusters (Fig 3A). For example, the pro-ED network includes a large cluster of genes associated with the inflammatory response, such as *PF4*, *CXCL12*, *CCL11* among several other chemokines, and another cluster comprising genes involved in extracellular matrix components and endothelial cell adhesion mediated by integrins like *ITGAV*, *VTN*, *ITGB3* and *ITGB8* during inflammation (Gao *et al*, 2019; Aman & Margadant, 2023). In contrast, the anti-ED network is characterised by a highly interconnected module (clustering coefficient = 0.151), with most of its central hubs belonging to the Activator Protein-1 (AP-1) family of transcription factors, such as *JUN*, *ATF*, *BATF* and *MAF* proteins (Fig 3B). *JUNB*, *FOSB*, *MAFG* and *ATF3* all presented a protective effect in ECs under pathological stimuli when silenced (Fig 2F).

Comparing the semantic similarity between the genes in the pro- and anti-ED network indeed shows that the pro-ED network comprises more functionally diverse genes (Wilcoxon rank test *P*-value - < 0.0005; Fig EV5B). However, classic functional enrichment analysis shows that the pro-ED network is mostly enriched in inflammatory pathways, whereas the anti-ED network is more enriched in gene regulation, angiogenesis, hypoxia and some low level of inflammation pathways (Fig 3C). The difference likely arises because the genes in the pro-ED network seem to have more specific functions compared to those in the anti-ED one, which seems to be associated with more processes and pathways, leading to enrichment of more diverse processes overall (Fig EV5C). The network boosted the signal for the mentioned pathways when comparing to initial list of pro- and anti-ED genes (Fig EV5D and E).

Both networks demonstrate similar enrichment terms for cardiovascular phenotypes, such as cardiovascular diseases and atherosclerosis, in the context of disease-enriched terms (Fig 3D). However, the nodes within the anti-ED network seem to exhibit a higher enrichment in non-direct cardiovascular phenotypes, including cancer diseases, asthma or degenerative polyarthritis, with the latter being the top enriched term for the anti-ED nodes (Fig 3D).

Finally, we mapped the targets of known drugs for cardiovascular diseases (CVDs) on both pro- and anti-ED networks (Dataset EV5), to see if there are any differences in the way these networks are targeted in the clinic. We identified 18 and 17 target genes for pro-ED and anti-ED, respectively, including *ITGAV*, *SERPINE1*, *PLAU* and *NFKB1*, which we found to be essential for ECs during dysfunction (Dataset EV5). This is consistent with our disease enrichment analysis, which found similar enrichment for CVDs for both networks (Fig 3D). However, anti-ED nodes are also substantially more enriched for drugs and diseases not related to CVD, likely due to their pleiotropic functions (Fig EV5C).

Independent drug screen corroborates findings and provides additional support for ED targets

As an independent validation of the findings from the siRNA screen, we conducted our phenotypic experiment using drug perturbations instead of siRNA. To identify potential drugs targeting genes among our 81 ED genes, we utilised the ChEMBL and OpenTargets databases (Willighagen *et al*, 2013; Ochoa *et al*, 2021) and selected 17 drugs based on their low Kd/Ki/IC50 values, physicochemical properties (Zhu *et al*, 2013), the assay and cell type used in determining their Kd/Ki/IC50, and their commercial availability (Materials and Methods). These drugs were chosen irrespective of the results from the siRNA screen (Table EV1 and Dataset EV6).

Before proceeding with the imaging experiment, we confirmed that neither the selected drugs nor the dimethyl sulfoxide (DMSO) vehicle control exhibited cytotoxicity compared to our death control, where cells were starved for 24 h (Fig EV6A and B). Subsequently, we replicated the imaging experiment described above, using two

© 2023 The Authors

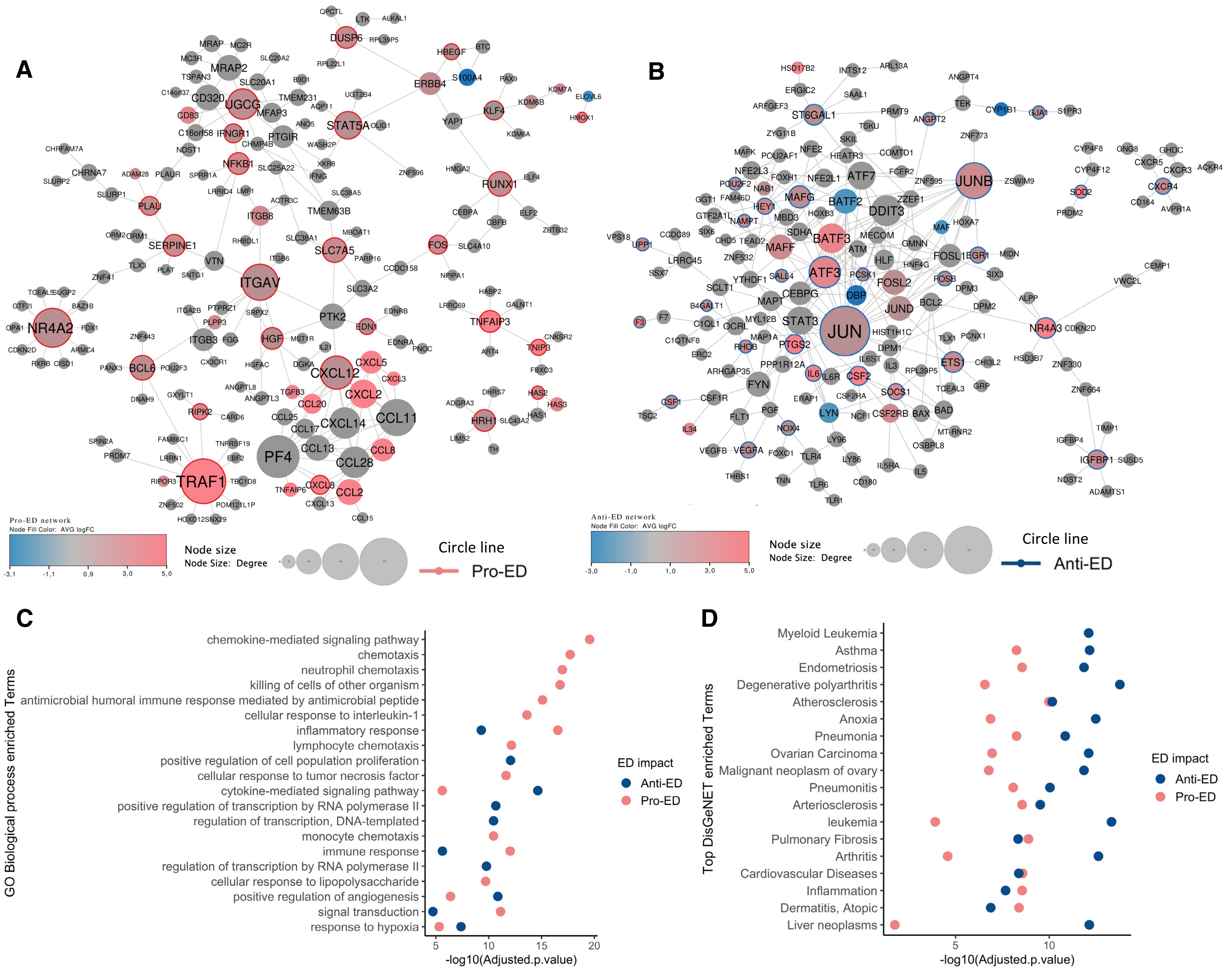

Figure 3. Network propagation from knockdown genes. ED protein–protein interaction (PIN) disease network generated with 26 pro-ED and 31 anti-ED genes as seeds and the random-walk-with-restart (RWR) algorithm.

A The pro-ED network comprises 176 nodes and 224 edges.

B The anti-ED network comprises 167 nodes and 268 edges. Colour reflects the average $\log_2$ fold change in the gene expression of these genes across the datasets integrated in the study. The size of the node represents the degree, i.e., number of interacting partners. The colour of the outline of the nodes reflects whether it was in the anti- or pro-ED gene list where relevant.

C GO enrichment analysis for Biological Processes for each one of the networks (adjusted P-value $\leq$ 0.05)

D DisGeNET (Piñero *et al*, 2019) enrichment analysis for disease-enriched terms for both networks.

Source data are available online for this figure.

concentrations of the drugs (10^{-6} and 10^{-8} M) under pathological stimuli conditions (Figs 4A and EV6C–F).

We treated HAECs with IL-1β and OxPAPC as above and used atorvastatin (5 μM) as a positive drug control. Atorvastatin is widely used in patients at high risk of CVD due to its beneficial effects on endothelial function (Fig EV6C–F) (Stancu & Sima, 2001; Oesterle *et al*, 2017). Atorvastatin demonstrated its expected antioxidant effect by suppressing ROS levels but did not modify ICAM1 expression, and significantly decreased VE-cadherin and nuclei counts compared to the pathological stimulus alone (Fig EV6C–F). Regarding nuclei counts, inhibitors of STAT5A (10^{-6} M) and CXCR4 reduced the number of cells, while several other inhibitors prevented the cell death compared to IL-1β and OxPAPC treated cells (Figs 4A and EV6C).

The drugs were mapped to its targets (Fig 4B and Table EV1). Drug treatment with AMD-070 hydrochloride, an inhibitor of CXCR4 suppressed IL-1β and OxPAPC-induced ICAM1 expression, while inhibitors of STAT5A (PD 180970), JUN (curcumin) and NQO1 (dicoumarol), increased ICAM1 staining (Figs 4A and EV6D). The VE-cadherin marker was significantly decreased when ITGAV (cilengitide), STAT5A (PD 180970), RIPK2 (capresla), JUN (curcumin), NAMPT (daporinad) and CXCR4 (AMD-070) were targeted, while targeting DUSP1 (CHEMBL6026) and IL6 (prednisolone) increased VE-cadherin staining (Figs 4A and EV6E).

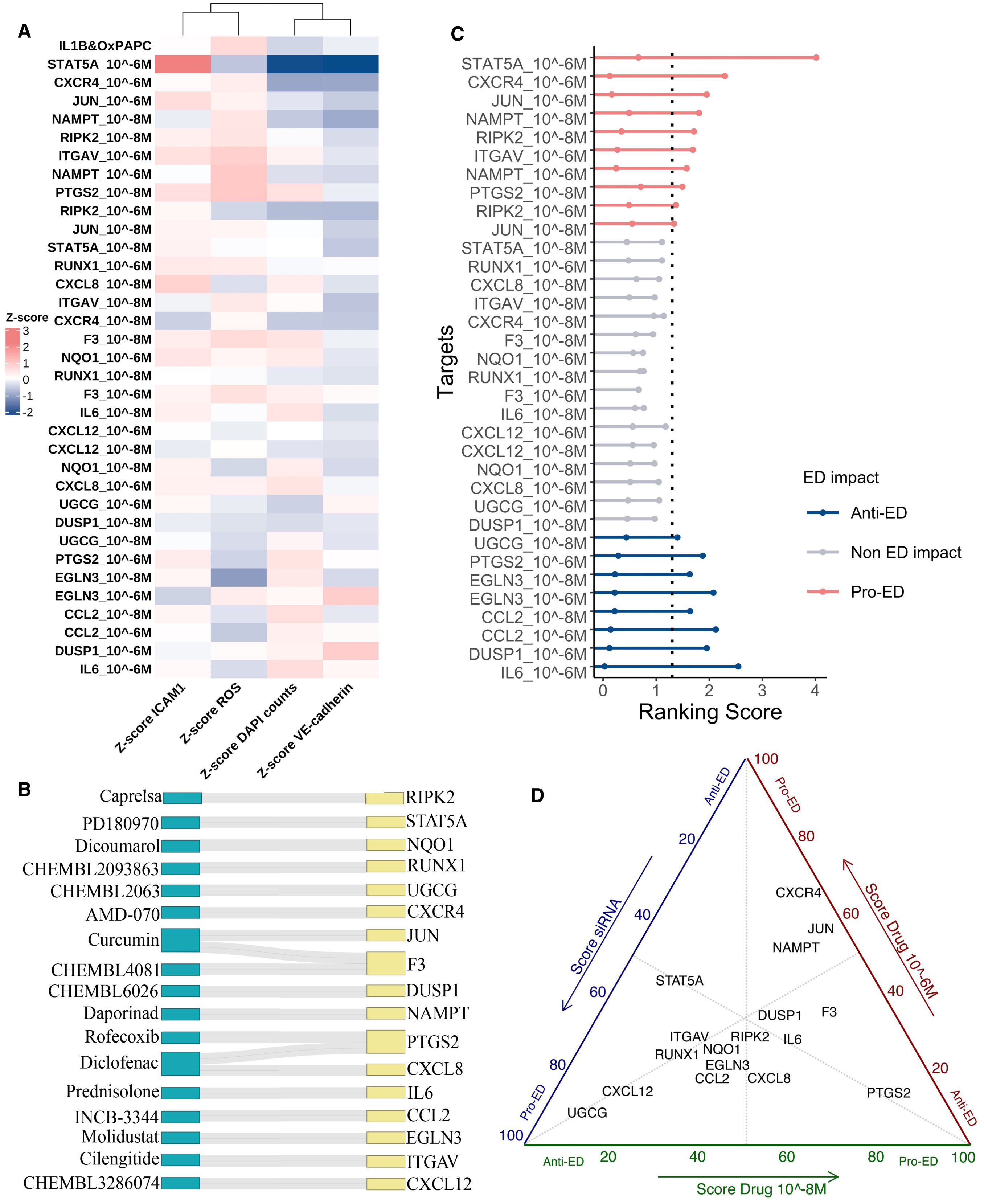

Figure 4.

© 2023 The Authors

Figure 4. Drug treatment high content screen data.

A Heatmap summarising the outcome of the immunofluorescence assays for ICAM1 expression (antibody anti-ICAM1), ROS intensity (probe CM-H2DCFDA), VE-Cadherin expression (antibody anti-VE-cadherin) and nuclei counts (DAPI). Colour reflects the median *Z*-score from all replicates. All experiments were repeated 2 times independently (biological replicates), with 4–5 technical replicates from each condition. All the comparisons were made between HAECs treated with each of the 17 drugs in both concentrations (10^{-6} and 10^{-8} M) versus HAEC with DMSO solvent control and treated with IL-1β and OxPAPC.

B Sankey diagram displaying the drug name and its target genes.

C Aggregated rank analysis for drugs exacerbating (red) and reducing (blue) ED phenotypes upon treatment. The first ranking, from left to right, rates drugs by *Z*-scores, with increased ICAM1 and ROS and decreased DAPI and VE-cadherin. These are pro-ED drugs. The second ranking is for anti-ED drugs, with opposite criteria (Materials and Methods). Both are displayed on the same plot, represented by the 2 dots per drug dose.

D The ternary plot summarises the results distribution from the siRNA screening and drug screening. Each vertex represents the results of one screening (siRNA, 10^{-6} M dose and 10^{-8} M). The scores were scaled from 0 to 100, where 0 represents an anti-ED outcome and 100 a pro-ED result.

Source data are available online for this figure.

Molidustat, a drug targeting EGLN3, displayed significant effects on three out of four ED phenotypes, suppressing IL-1β and OxPAPC-induced ICAM1 expression (Figs 4A and EV6D) and ROS production (Figs 4A and EV6F) and increasing VE-cadherin staining (Figs 4A and EV6E). These results did not align with the effects obtained with siRNA knockdown of EGLN3 during our screen, where no significant effect was observed in any phenotype. Due to the well-described multi-target effect of molidustat for all PHD family members (Xie *et al*, 2012), we hypothesised that molidustat might be exerting its protective effect through targeting one of the other PHD family members, *EGLN1* or *EGLN2*. After confirming that the drug was functioning as expected in terms of stabilisation of HIF-1α and that IL-1β and OxPAPC did not change the HIF-1α protein content (Materials and Methods; Appendix Fig S1A–C; Appelhoffl *et al*, 2004; Luo *et al*, 2011; Xie *et al*, 2012), we performed a knockdown with siRNA for all the members of the PHD family, *EGLN1*, *EGLN2* and *EGLN3* increasing the number of biological replicates to 12, to increase our statistical power to detect effects (Appendix Fig S1D and E). Interestingly, the effect of the drug was not recapitulated, and, on the contrary, both *EGLN1* and *EGLN3* knockdown increased ROS, with EGLN1 knockdown also increasing ICAM1, in HAEC cells treated with IL-1β and OxPAPC (Appendix Fig S1D and E). We therefore hypothesise that either combinatorial inhibition of the PHD family proteins would be needed to reveal the full molidustat phenotype, or molidustat is functioning through alternative and unknown targets (Ivan & Kaelin, 2017). In the future, more detailed experiments are needed to understand the mechanisms underlying molidustat's protective effect in ECs.

To summarise the results of the drug screen, we once more performed an aggregated rank analysis (Fig 4C). Overall, among the drugs tested, those targeting STAT5A, ITGAV, RIPK2, IL6 and DUSP1 corroborate the results from the siRNA screen for all tested phenotypes, with STAT5A, ITGAV, and RIPK2 presenting a pro-ED phenotype, and IL6 and DUSP1 presenting an anti-ED phenotype when inhibited with a drug (Figs 4D and EV6G). Targeting CCL2 also showed a significant anti-ED effect in the drug screen, whereas in the siRNA screen it had narrowly missed the significance cut off. Our independent drug screen, therefore, provides additional support for IL6, DUSP1 and CCL2 as potential targets for ED (Fig EV6G).

Discussion

Endothelial dysfunction (ED) is a critical feature of cardiovascular disease (CVD), and research has established that individuals with impaired endothelium have a higher risk of experiencing cardiovascular events, such as heart attacks or strokes (Schächinger *et al*, 2000; Gokce *et al*, 2002; Matsuzawa *et al*, 2013). Despite this fact, most drugs developed for treating CVD, including atherosclerosis or hypertension, have not been specifically designed to target ED (Daiber *et al*, 2017; Hennigs *et al*, 2021; Libby, 2021).

In our study, we aimed to illuminate the molecular basis for ED and identify potential targets for therapeutic intervention. We identified 26 (pro-ED) genes that exacerbate ED, and 31 (anti-ED) genes that ameliorate ED when silenced. These findings allowed us to extract pro-ED and anti-ED network signatures, supported by *in vivo* analysis of scRNA-seq data in the context of atherosclerotic vasculature. The pro-ED network was enriched in inflammatory pathways and the anti-ED network was enriched in gene regulation, angiogenesis, and other cardiovascular and non-cardiovascular processes.

Endothelial gene expression is influenced hierarchically by various cardiovascular risk factors (Pinheiro-de-Sousa *et al*, 2022). Given the complex nature of ED's underlying pathophysiology, we hypothesised that identifying commonly deregulated genes associated with multiple surrogate risk factors would enhance the chances of successfully targeting ED. Since ED is a phenotypic outcome that is common to diverse environmental pressures (e.g. OSS, inflammation or oxidised lipids), we postulated that the changes they induce in the epigenome, reflecting the cell state, and that are associated with ED, should at least partially overlap. We, therefore, decided to focus on the epigenetic omics layer, concentrating on the common chromatin regions affected by all the diverse conditions and subsequently overlapping the associated differentially expressed genes, to improve our chances of identifying relevant ED genes. This approach indeed improved the signal-to-noise ratio, allowing us to prioritise a large number of genes (83% of the 81) with an effect on at least one of the ED phenotypes tested. However, we acknowledge that this analysis may have missed more understudied genes and transcription factors that are not well annotated. Our network propagation strategy to expand the pro- and anti-ED genes and identify the respective processes and network signatures partially addresses this issue by integrating genes not directly related to the identified transcription factors in the multi-omics integration step. However, it may still miss understudied genes from entirely different processes. Similarly, as most drugs tend to inhibit protein function and we aimed to identify targets for ED, we only focused on upregulated ED genes. Future studies should consider expanding the approach to include all differentially expressed genes, allowing the identification of additional potential targets, and gaining condition-specific insights.

Our approach allowed us to identify contrasting responses to the ED readouts of genes from the same family. Taking the AP-1 family for example, *FOS* presented a pro-ED phenotype when silenced,

© 2023 The Authors

while all the other members of its family, such as *JUN*, *JUNB*, *FOS*, *FOSB* and *ATF3* presented an anti-ED phenotype. AP-1 family members are known to exert broad effects on ECs, including inflammatory response modulation, regulation of angiogenesis, vascular remodelling, and oxidative stress (Yoshitomi *et al*, 2021). For example, our top anti-ED hit, *JUNB*, is implicated in angiogenesis and is induced by VEGF (Jia *et al*, 2016; Yoshitomi *et al*, 2021). VEGF stimulates NF-kB-dependent inflammatory gene expression to mediate its inflammatory effects (Kim *et al*, 2001; Jia *et al*, 2016), which explains our findings that *JUNB* knockdown suppressed ICAM1, presented an antioxidant effect, and increased cell viability. Another example of paralogs with opposite effects is *NR4A2* and *3* (Fig 2F). *NR4A3* is well-annotated with roles in metabolism and inflammation, and the proliferation, maintenance and differentiation of many cell types, including e.g. vascular smooth muscle (Martínez-González *et al*, 2021). *NR4A2*, on the other hand, is not as well-annotated, with its predicted function in the Uniprot database (Català-Solsona *et al*, 2021) being related to the differentiation and maintenance of meso-diencephalic dopaminergic (mdDA) neurons (Messmer *et al*, 2007). We found *NR4A2* to be essential in EC cells under the pathological stimuli and to increase ICAM1 expression upon knockdown, suggesting a potential compensatory mechanism for *NR4A3* deregulation in ECs.

Another interesting observation was that the pro-ED network, representing molecular process rewiring as an adaptation to the environmental conditions and ED, comprises more specialised genes involved in more varied cell processes than the anti-ED network. In contrast, the anti-ED network, representing deregulated processes more likely to be causal and therefore better targets, comprises more functionally similar genes, albeit with pleiotropic functions (Fig EV5C). This suggests that the cell adaptation for survival and maintaining functionality is more systemic and precise than causal deregulation. While most studies identifying causal disease genes are based on genetics (Liu & Montgomery, 2020; Ochoa *et al*, 2022), our study combining genetic perturbations with functional readouts and network analysis can distinguish deregulated from adapted networks and processes as opposed to individual genes and can provide functional information and insight into the molecular basis of the disease, further improving target prioritisation.

Moreover, we demonstrated that the commonly used practice of identifying deregulated genes in disease omics datasets and then considering them as putative targets may have a major drawback that it might result in erroneous targeting of non-causal processes, potentially exacerbating the disease phenotype. For example, we found 18 drugs that are used to treat cardiovascular diseases that overlap with the pro-ED network, raising concerns about their suitability as therapeutic options.

Our independent drug screen for 17 among the 81 genes generally corroborated the results of the siRNA screen. We found that the results from drugs targeting IL6, DUSP1, and CCL2 also agreed with the results from the siRNA screen (albeit CCL2 marginally missing the significance cut off in the siRNA screen results) (Fig EV6G). This orthogonal support makes them particularly attractive as targets for ED. In terms of drug repurposing opportunities, at present, the glucocorticoid prednisolone (targeting IL6) is used for many inflammatory diseases (Coutinho & Chapman, 2011). However, due to its association with elevated CVD risk upon chronic or excessive use (MacLeod *et al*, 2021), it may not be suitable for repurposing as a therapeutic agent for heart disease. Instead, ziltivekimab, an IL-6 ligand inhibitor (Ridker & Rane, 2021), could be a more promising alternative for repurposing, considering the protective effect of silencing IL6 on ECs. DUSP1, a crucial regulator of MAPK signalling pathways (Wang *et al*, 2016), has shown promise as a combination target in the context of cancer progression (Liu *et al*, 2014). The compound we used for DUSP1 is still in too early tests to be considered for repurposing at present, but it would be worth considering it in the future if it proves to be safe. Finally, CCL2 has a vast literature supporting its role in inflammation and is also considered a target for atherosclerosis (Soehnlein & Libby, 2021). Therefore, the drug used here INCB3344/CCL2 (Shin *et al*, 2009) might be a suitable candidate for repurposing. Molidustat, an inhibitor of the EGLN protein family, demonstrated both antioxidant and anti-inflammatory effects, making it a promising candidate for drug repurposing. EGLN3/PHD3 functions by hydroxylating proline residues to stabilise proteins like HIF-1α and prevent their degradation (Appelhoffl *et al*, 2004). Several studies have associated HIF-1α with lower levels of ROS in myocardial ischemia–reperfusion injuries (Vogler *et al*, 2015; Neckář *et al*, 2018) and ED (Rahtu-Korpela *et al*, 2016; Abe *et al*, 2017). However, the molidustat-based results were not reproduced by silencing the EGLN protein family members. This suggests that combinatorial silencing of these proteins is required, or an alternative mechanism of action of molidustat might be involved. More in-depth investigation is outside the scope of this manuscript but could be worthwhile in the future.

Overall, our findings highlight the potential of integrating omics datasets from diverse conditions and employing network analysis and *in vitro* genetic and pharmacologic validation, to identify networks relevant to common phenotypes like ED, distinguish causal from adaptive processes, and prioritise targets for therapeutic modulation of these phenotypes. While these networks may not comprehensively represent the molecular processes underlying such a complex disease, they are highly enriched in relevant genes, providing a strong foundation for target identification.

Materials and Methods

Reagents and Tools table

Reagent/Resource	Reference or source	Identifier or catalogue number
**Experimental models**		
HAEC (*H. sapiens*)	Gibco	C0065C
**Antibodies**		
Anti-ICAM-1 monoclonal antibody, mouse (1:200 IF)	Cell Signalling	62133

 © 2023 The Authors

Reagents and Tools table (continued)

Reagent/Resource	Reference or source	Identifier or catalogue number
Anti-VE-cadherin monoclonal antibody, rabbit (1:200 IF)	Cell Signalling	2500
Goat anti-Rabbit IgG (H+L) Cross-Adsorbed secondary antibody, HRP	ThermoFisher Scientific	G-21234
Anti-mouse 555 goat secondary antibody (1:500 IF)	ThermoFisher Scientific	A21422
Anti-rabbit 647 goat secondary antibody (1:500 IF)	ThermoFisher Scientific	A21244
Anti-HIF1α monoclonal antibody, rabbit (1:500 WB)	Cell Signalling	36169
Anti-GAPDH polyclonal antibody, rabbit (1:2,000 WB)	Abcam	ab22555
**Oligonucleotides and other sequence-based reagents**		
siRNA	Dharmacon (Horizon Discovery)	
ALDO A primers	ThermoFisher Scientific<br>This study	
LDHA primers	ThermoFisher Scientific<br>This study	
CXCR4 primers	ThermoFisher Scientific<br>This study	
NOS3 primers	ThermoFisher Scientific<br>This study	
Cyclophilin primers	ThermoFisher Scientific<br>This study	
**Chemicals, Enzymes and other reagents**		
EGM-2 Endothelial Cell Growth Medium-2 Bullet Kit	Lonza	CC-3162
Lipofectamine RNAiMAX Transfection Reagent	ThermoFisher Scientific	13778150
Opti-MEM Reduced Serum Medium	Gibco	31985070
Hoechst 33342	ThermoFisher Scientific	H3570
DMSO	Sigma-Aldrich	D8418
IL-1β	Peprotech	200-01B
OXPAC	Hycult Biotech	HC4036
CM-H2DCFDA	ThermoFisher Scientific	C6827
Forbol 12-miristato-13-acetato (PMA)	Sigma-Aldrich	P8139
H_2O_2	Sigma-Aldrich	H1009
Atorvastatin	Sigma-Aldrich	SML3030
Mitosox	ThermoFisher Scientific	M36008
Paraformaldehyde (PFA)	Sigma-Aldrich	158127
Triton X-100	Sigma-Aldrich	T8787
Bovine Serum Albumin (BSA)	Sigma-Aldrich	A7906
DAPI	ThermoFisher Scientific	D3571
RIPA lysis buffer	Millipore	20-188
Protease Inhibitor Cocktail	Sigma-Aldrich	P8340
Phosphatase Inhibitor Cocktail 2	Sigma-Aldrich	P5726
Phosphatase Inhibitor Cocktail 3	Sigma-Aldrich	P0044
PVDF transfer membranes	Millipore	IPVH00010
TRIzol reagent	ThermoFisher Scientific	15596026
SuperScript IV Reverse Transcriptase	ThermoFisher Scientific	18090010
**Software**		
Bowtie2	Langmead and Salzberg (2012)	
MACS2 tool	Kim and Dekker (2018)	
rtracklayer and GenomicRanges	Lawrence *et al* (2013) and Lawrence *et al* (2009)	
ChIPSeeker R package	Yu *et al* (2015)	

© 2023 The Authors

Reagents and Tools table (continued)

Reagent/Resource	Reference or source	Identifier or catalogue number
ChIPpeakAnno R package	Zhu *et al* (2010)	
rGADEM and MotIV	R packages	
HTSeq2	Putri *et al* (2022)	
DESeq2 R package	Love *et al* (2014)	
TRRUST database v.2	Han *et al* (2018)	
R package Seurat v3.2.292	Hao *et al* (2021)	
ClusterProfiler package	Yu *et al* (2012)	
PageRank	http://igraph.org	
OpenTargets	Ochoa *et al* (2021)	
ChEMBL	Willighagen *et al* (2013)	
CellProfiler v4.2.5	https://cellprofiler.org/releases<br>Carpenter *et al* (2006)	
CellProfiler plugins	https://arxiv.org/abs/2306.01915<br>preprint: Tromans-Coia *et al* (2023)	
ImageJ	https://imagej.nih.gov/ij/download.html<br>Schneider *et al* (2012)	
**Other**		
LIVE/DEAD Viability/Cytotoxicity Kit	ThermoFisher Scientific	L3224
Pierce BCA Protein Assay Kit	ThermoFisher Scientific	23225
QuantiTect SYBR green PCR kit	Qiagen	204143
EVOS M7000 image system	ThermoFisher Scientific	AMF7000
ImageQuant LAS 4000 mini	GE Healthcare	
QuantStudio 12 K Flex system	ThermoFisher Scientific	

Methods and Protocols

Datasets

To identify the ED genes, we integrated publicly available RNA-Seq, H3K27ac ChIP-Seq, and ATAC-Seq datasets (GSE89970, GSE112340) from cells HAECs. In the GSE112340 dataset (Data ref: Krause *et al*, 2018b), data from HAECs was acquired either in static conditions or after 24-h unidirectional or disturbed flows. In the GSE89970 (Data ref: Hogan *et al*, 2017b), HAECs were subjected to inflammatory cytokines TNF-α (10 ng/ml), IL-1B (10 ng/ml) or OxPAPC (40 μg/ml) for 4 h.

ATAC-seq and ChIP-seq analysis

For the ChIP-seq and ATAC-seq data, we used the pre-processed hg19-aligned data using Bowtie2 (Langmead & Salzberg, 2012). Peaks were identified using the MACS2 tool (Kim & Dekker, 2018). The BED files from multiple replicates were compiled through consensus voting (https://ro-che.info/articles/2018-07-11-chip-seq-consensus), where the peaks considered were the overlapping peaks between replicates (packages in R, *rtracklayer* and *GenomicRanges*) (Lawrence *et al*, 2009, 2013). ATAC-Seq and ChIP-Seq consensus peaks were identified and annotated using the *ChIPSeeker* R package (Yu *et al*, 2015).

We integrated the ChIP-seq and ATAC-seq data by identifying all the peaks that overlap in all conditions using the *ChIPpeakAnno* R package (Zhu *et al*, 2010). We used the findOverlappingPeaks() function, which uses as input the consensus peaks identified previously for each condition. We considered two peaks overlapping when one resided entirely within the second one. We identified transcriptionally active regions by applying the findOverlappingPeaks() function to centre the ChiP peaks onto the open chromatin ATAC peaks regions. Thus, this final merged BED peak file contained regions common to all stimuli and regions of open chromatin and active enhancers.

Motif enrichment analysis

For *de novo* motif enrichment, we used *rGADEM* and *MotIV* (R packages) to predict active TFs from the merged BED peak file containing a 200 bp sequence. The *rGADEM* package combines a genetic algorithm (GA) with an expectation-maximisation (EM) algorithm to identify the statistically significant motifs. We considered the motifs whose scores reached the significance threshold of $E < 0.05$. Then, we used MotIV, the JASPAR database (Castro-Mondragon *et al*, 2022), and the motifs identified by *rGADEM* to identify the TFs. The motifMatch() provided the TFs alignment to the input motifs and their *P*-value. We kept all TFs statistically significant ($P < 0.05$).

RNA-seq analysis and TF-DEGs associations

The RNA-seq samples were subjected to fastQC quality control, where the sequences were trimmed by 13 bp to create the SAM files, later converted to BAM and aligned to hg19 using HTSeq2 (Putri *et al*, 2022). Principal component analysis, batch effect correction, and differential expression analysis were performed using the

 © 2023 The Authors

DESeq2 R package (Love *et al*, 2014) accounting for the batches within datasets. We applied the rlog transformation to the normalised counts to improve the distances and clustering for the PCA and hierarchical clustering-based visualisation methods. For the PCA analysis, we utilised the DESeq2 package using as directly input the rlog object. By default, the function uses the top 500 most variable genes for the analysis, which is the number of genes used for the PCA and hierarchical clustering of the samples.

Differential expression analysis was performed for each condition (IL-1β, TNF-α and OxPAPC) compared to static baseline control, while OSS was compared to LSS. We consider as DEGs those with an adjusted $P < 0.05$ and $|\log_2\text{Foldchange}| > 1.5$. Using the merged BED peak from the ChIP and ATAC overlapping peaks and their annotated genes, we filtered by identifying which DEGs were present in those peaks. We used the TRRUST database (version 2) (Han *et al*, 2018) to map the TFs identified to their previously published transcription targets. We considered only the TFs and DEGs whose interactions were supported by published evidence (Fig 1F).

siRNA library design

Commercially available siRNAs (Dharmacon/Horizon Discovery) were used to knockdown each of the 81 prioritised genes. To reduce off target effects and guarantee higher efficiency in silencing each target, we used their SMARTpool technology that combines 4 individual siRNAs for each gene target (ON-TARGET plus siRNA).

ChEMBL and OpenTargets drug selection

We used the OpenTargets (Ochoa *et al*, 2021) and ChEMBL (Willighagen *et al*, 2013) databases to identify potent known inhibitors/antagonists for prioritised targets above. ZINC database (Irwin *et al*, 2020) was used to confirm the molecule structure and vendors. Each target had a list of active compounds with increasing values of Kd/Ki - or IC_{50} if Kd/Ki data was not available. The active compound with the lowest Kd/Ki/IC_{50} was then checked for assay data quality (assay type, cell type), physicochemical properties (log *P* and molecular weight) (Zhu *et al*, 2013) and commercial availability (Sterling & Irwin, 2015). The target of each drug was identified primarily using the ChEMBL 26 database, ZINC database (Irwin *et al*, 2020) and DrugBank.

Cell culture and ED induction

Human aortic endothelial cells were purchased from Gibco. They were cultured in medium EGM-2 (LONZA), incubated at 37°C and 5% CO_2. All experiments were performed between passages four and seven. Cells were tested for mycoplasma contamination (Young *et al*, 2010). To recapitulate at least partially the epigenetic overlapped regions identified in this study, we cultivate the HAEC under IL-1β (10 ng/ml, Peprotech) and OxPAPC (50 μg/ml, Hycult Biotech) stimuli.

siRNA transfection

siRNA was transfected into HAECs using a standard reverse transfection. Transfection mixes were prepared with lipofectamine RNAiMAX (ThermoFisher Scientific) and siRNA against the target genes (25 nM), or scrambled control siRNA (25 nM) diluted in Opti-MEM (Gibco), according to the manufacturer's instructions. Cells were seeded into 96-well plates (3×10^4 cells per well) pre-coated with gelatin 0.1%, and 20 min later we added the transfection complex to each well. Cells were incubated at 37°C for 6 h. Then we added an additional volume of fresh regular culture medium.

Viability assay

Human aortic endothelial cells were tested for cell viability 72 h after the siRNA transfection protocol and after 48 h treatment with the 17 inhibitory or antagonistic molecules at 10^{-6} and 10^{-8} M concentrations. We used the LIVE/DEAD Viability/Cytotoxicity kit (ThermoFisher Scientific) for this assay. At the end of both incubation time, the medium was carefully removed, and the fluorescent probes were added to react with live (calcein-AM, 2 μM, Ex/Em 494/517) and dead (ethidium homodimer-1, 4 μM Ex/Em 528/617) cells, with a cell nucleus marker (Hoechst 33342 8.1 μM – Ex/Em 361/486) (ThermoFisher Scientific) diluted in phosphate-buffered saline (PBS). The probes were incubated for 30 min at room temperature and then removed. Finally, cells were maintained in PBS for image acquisition with the EVOS M7000 Image system (ThermoFisher Scientific). Nine pictures were taken per well at 10× magnification. We used HAECs stimulated with DMSO (at the same proportion of the highest dose 10^{-6} M of the molecules, Sigma-Aldrich) as basal control for drugs. As a positive death control for the drug screening, cells were cultured in a starving medium for 24 h. For the siRNA, we used siPLK1 to induce EC cell death. The cell death index (ethidium and calcein index) was calculated by counting the number of positive-staining cells divided by the total number of Hoechst-positive cells multiplied by 100.

siRNA screening

After 48 h of the transfection protocol, the medium of HAECs in the 96-well plate was carefully removed and IL-1β (10 ng/ml, Peprotech) and OxPAPC (50 μg/ml, Hycult Biotech) were added to the medium. We used as an additional control HAECs transfected with scrambled control siRNA at complete medium without IL-1β and OxPAPC. After 24 h, the cells were incubated with the CM-H2DCFDA probe (5 μM, ThermoFisher Scientific) for 30 min at 37°C to evaluate the generation of ROS. Then, the cells were fixed with 4% PFA for 15 min at room temperature. CM-H2DCFDA fluorescence intensity was analysed at an excitation wavelength of 492–495 and an emission wavelength of 517–527 nm.

Drug screening

Human aortic endothelial cells were plated in 96-well plates (3×10^4 cells per well) for drug screening. We pre-treated the cells with 17 drugs diluted in a complete culture medium (EGM2) at 10^{-6} and 10^{-8} M for 2 h. Then, we added IL-1β (10 ng/ml, Peprotech) and OxPAPC (50 μg/ml, Hycult Biotech) to the medium. HAECs were cultured for 50 h (2 h pre-treatment with drugs + 48 h with drugs and stimulation with IL-1β and OxPAPC). We used as baseline controls HAECs at complete medium without DMSO, with DMSO (same proportion of drugs at 10^{-6} M, Sigma-Aldrich), and with IL-1β and OxPAPC (without drugs). As an additional control (not in the wells treated with drug), before following up with the immunofluorescence assay, we used Forbol 12-miristato-13-acetato (PMA, 10 μM, Sigma-Aldrich) and H_2O_2 (200 μM, Sigma-Aldrich) for 1 h before the end of the incubation period to stimulate ROS production. We used atorvastatin (5 μM, Sigma-Aldrich) to compare with the molecules tested. We followed the same approach, 2 h pre-

© 2023 The Authors

treatment with atorvastatin (5 μM), followed by 48-h stimulation with IL-1β and OxPAPC.

After the end of the experiment, the cells were incubated with CM-H2DCFDA (5 μM, ThermoFisher Scientific) and MitoSox (5 μM, ThermoFisher Scientific) for 30 min at 37°C to evaluate ROS generation. The cells were fixed with 4% Paraformaldehyde (PFA, Sigma-Aldrich) in PBS for 15 min at room temperature. Mitosox fluorescence intensity was analysed at an excitation wavelength of 5,105 and an emission wavelength of 580 nm.

Immunofluorescence and high-content screening analyses

We followed the immunofluorescence protocol with permeabilisation for 1 h with Triton X-100 (0.1% in PBS, Sigma-Aldrich), blocking with bovine serum albumin (BSA, 5% in PBS, Sigma-Aldrich) for 1 h at room temperature and incubation with the anti-ICAM1 antibody (Cell Signalling#62133/1:200), anti-VE-cadherin antibody (Cell Signalling#2500/1:200) and anti-CD62E/E-selectin antibody (Abcam#ab18991/1:100) at 4°C overnight. After that, we washed with PBS (2×) and followed the incubation with DAPI (1 mg/ml, ThermoFisher Scientific) and with the fluorescent secondary antibodies (goat-anti rabbit and goat anti-mouse Alexa Fluor, ThermoFisher Scientific, 1:500). Finally, we performed two more washes with PBS, and the cells were kept in PBS for image acquisition.

Image acquisition and analysis

We used an EVOS M7000 Image system (ThermoFisher Scientific). Images were obtained at 10× magnification, with 9–12 images per well. After the acquisition, the raw images were analysed using CellProfiler (Carpenter *et al*, 2006) software. The image analysis pipeline included both runStardist and runCellpose modules to more accurately segment nuclei and cells, respectively. Standard parameters were used for the runStardist module, while flow threshold was set to 0.4 and object diameter was set to 60 for the runCellpose module. Next, we quantified the signal intensity of multiple markers for the identified cells. We considered the median intensity for CM-H2DCFDA, and MitoSox, the median edge intensity (EDGE) for ICAM-1, and VE-cadherin, and cytoplasmic staining for VE-cadherin. We considered the ratio of median EDGE intensity/median cytoplasm intensity for VE-cadherin membrane location. We considered the median intensity of each well. We also used the EDGE for ICAM-1 to calculate the eccentricity as the parameter to measure cell morphology. Parameters measured in addition to the intensity signal included cell area, length and circularity using the ICAM-1 marker.

Image statistical analysis

We performed the normalisation of the median intensities of CM-H2DCFDA, ICAM1, VE-cadherin and nuclei counts quantified using CellProfiler (McQuin *et al*, 2018), with controls [normalised values = median intensity value/mean (median intensity control samples)] to normalise the median intensity of each well's values within each plate. We did this for both screenings, using siRNA scramble IL1B&OxPAPC or IL1B&OxPAPC plus vehicle (DMSO) as controls. Then, to consider the plate-to-plate variation, we computed the Z-score for all plates by subtracting the mean and dividing by the standard deviation of all samples. Bootstrapping, a resampling technique, was employed to assess the robustness and reliability of the Z-score values obtained from comparing controls (IL1B&OXPAPC) with siRNA-treated cells. This method is particularly useful for our unbalanced samples, which have 8 times more controls compared to our treated sample sizes. We performed 1,000 times the resampling of the IL1B&OXPAPC (sample size = 8) and performed 1,000 times the multiple pairwise comparisons with the Wilcoxon test, where we compared all conditions versus IL-1B-&OxPAPC controls. We considered the median P-value < 0.05 for significant hits.

Protein extraction, Western blot and analysis

Human aortic endothelial cells were plated in 12-well plates (2×10^5 cells per well) for protein extraction. We treated cells with IL-1β (10 ng/ml) and OxPAPC (50 μg/ml), Molidustat with a range of doses from 10^{-8} M to 100×10^{-6} M or Cobalt Chloride (CoCl2, 300 μM) for 48 h. Cells were lysed in RIPA lysis buffer (Millipore) containing protease and phosphatase inhibitors (Sigma-Aldrich) and proteins quantified using a BCA protein assay kit (Thermo Fisher Scientific). Equivalent amounts of proteins were solubilised in sample buffer (0.5% SDS, 10% glycerol, 0.05% bromophenol blue, 50 mM dithiothreitol, 50 mM Tris, pH 6) and subjected to sodium dodecyl sulfate-polyacrylamide (SDS/PAGE) gel electrophoresis. After electrophoresis, the proteins were transferred to polyvinylidene fluoride (PVDF) membranes (Millipore). The membranes were first incubated in blocking solution (5% BSA and 0.1% Tween 20 in tris-buffered saline, pH 7.4) for 1 h at room temperature, followed by incubation for 16–18 h in primary antibody against HIF-1α (Cell Signalling #36169/ 1:500) diluted in blocking solution at 4°C. Membranes were also incubated with primary antibodies specific for GAPDH (Abcam#ab22555/1:2,000) as an internal control (housekeeping). After incubation for 1 h at room temperature with a secondary antibody conjugated to horseradish peroxidase (ThermoFisher Scientific#G-21234/1:2,000), the bound primary antibody was detected using a chemiluminescent image analyser (ImageQuant LAS 4000 mini, GE Healthcare), and images were quantified by densitometry using ImageJ (Schneider *et al*, 2012). All the full-size Western blots are shown in Appendix Fig S2.

RNA extraction, qPCR and analysis

Human aortic endothelial cells were plated in 12-well plates (2×10^5 cells per well) for RNA extraction. We treated cells with Molidustat (10^{-8} and 10^{-6} M) or Cobalt Chloride (CoCl2, 300 μM) for 48 h. HAECs were lysed with 1 ml of TRIzol Reagent (ThermoFisher Scientific), and the total RNA was extracted according to the manufacturer's instructions. After extraction and purification, RNA was reverse transcribed using SuperScript IV Reverse Transcriptase (ThermoFisher Scientific). Real-time PCR was performed using QuantiTect SYBR green PCR kit (Qiagen) with the QuantStudio 12 K Flex system (ThermoFisher Scientific). We used Cyclophilin Ct values to normalise ΔCt. The variation in gene expression between samples was calculated using the ΔΔCt method.

The following primers were used (5′ to 3′): ALDO A Forward: ACACTCTACCAGAAGGCGGA, Reverse: CCAACCCTTGGGTGGTAG TC; LDHA Forward: GTGTGCCTGTATGGAGTGGAA, Reverse: CAA CCACCTGCTTGTGAACC; CXCR4 Forward: GGAGGGGATCAGTATA TACACTTCA, Reverse: TGATGGAGTAGATGGTGGGC; NOS3 Forward: GCACAGTTACCAGCTAGCCA, Reverse: GCCGGGGACAGG AAATAGTT; Cyclophilin Forward: CATTTGGTGCAAGGGTCACA; Reverse: TCTGCTGTCTTTGGGACCTTGTC.

 © 2023 The Authors

Statistical analysis

For qPCR and Western blot, we used Student's *t*-test or one-way analysis of variance, followed by the Benjamini–Hochberg (BH) *post hoc* test. We analysed variance using Kruskal-Wallis ranks for non-parametric values, followed by the Wilcoxon test for multiple comparisons. Differences were significant when $P < 0.05$. For all statistical analyses, we used R software (version 4.1.0). All the experiments were performed at least two times (one biological replicate for each time) with 1–8 technical replicates. Outliers were removed based on the Z-score methods with an absolute value of 3.

Aggregated rank analysis

We used the R packages RobustRankAggreg (Kolde *et al*, 2012) to integrate the rankings obtained from the *in vitro* experiments based on the Z-score (image data analysis) of the 4 phenotypes tested: ICAM1 expression, ROS intensity, VE-cadherin expression and nuclei counts, and generated an overall ranking of the 81 KO genes. To generate the pro-ED ranking, we reorder the Z-score values of ICAM1 and ROS, from highest to lowest value, and for VE-cadherin and nuclei counts, from lowest to highest values. For the anti-ED ranking, we reversed the orders. We repeated the same analysis for the drug screening results. These packages implement a robust rank aggregation method that combines multiple rankings into a consensus ranking. The top-ranked genes in the list were considered the most important in terms of their combined values across the different analyses. A null model was employed to determine the statistical significance of gene rankings. The simplest null model assumed that all genes were non-informative and generated randomly ordered gene lists. This null model corresponded to a permutation test, where the *in vitro* measurements were randomly assigned before the aggregation analysis step. This analysis allowed us to assess the robustness of the rankings and identify ED genes that were significantly different from random.

scRNAseq data analysis

To scRNAseq data for the orthogonal validation of the endothelial dysfunction (ED) disease network *in vivo* consisted of two atherosclerosis lesion datasets and one Tabula sapiens control dataset (Data ref: Wirka *et al*, 2019b; Data ref: Alsaigh *et al*, 2022b; Data ref: Jones *et al*, 2022b). Firstly, we integrated the three datasets using the R package Seurat version 3.2.292. Here, we applied a method for integrating the datasets called canonical correlation analysis (CCA) (Hardoon *et al*, 2004). CCA identifies shared sources of variation between the conditions/groups. The 3,000 most variant genes from each sample were used for this analysis. This step aligns the cells using the greatest shared sources of variation. The shared highly variable genes are used because they are most likely to represent the genes distinguishing different cell types present. Next, we identify anchors or mutual nearest neighbours (MNNs) across datasets: MNNs can be thought of as "best buddies." For each cell in one condition, the closest neighbour in the other condition is identified based on gene expression values. Anchors and their corresponding scores are used to transform the cell expression values, allowing for the integration of the conditions/datasets. The transformation of each cell uses a weighted average of the two cells of each anchor across anchors of the datasets. The weights are determined by the cell similarity score (distance between cell and k nearest anchors) and anchor scores, ensuring that cells in the same neighbourhood have similar correction values. If cell types are present in one dataset but not the other, they will still appear as separate sample-specific clusters. This approach allowed us to cluster all the ECs independently of the vascular region which we considered each location as a different batch.

For further analysis, we applied data pre-processing steps, including filtering out cells expressing fewer than 200 genes and genes expressed in fewer than three cells. We also excluded cells with unique gene counts over 2,500 or less than 200. Cells with over 5% mitochondrial genes, indicating poor quality, were removed. Principal component analysis (PCA) was then performed on the scaled data for dimensionality reduction. To refine the cell grouping, we used the FindNeighbors() and FindClusters() functions on the PCA space, calculating Euclidean distances between cells to iteratively group cells with similar expression patterns. The resulting clusters were visualised using UMAP. We identified cluster markers using the FindMarkers() function, which compared each cluster against all others through differential expression analysis. We selected gene markers with a P-value ≤ 0.05 and an average $|\log_2\text{Foldchange}| \geq 0.25$. The top cell markers were manually used to annotate each cell type. The differential expression analysis for the ECs considered as disease condition the two atherosclerotic datasets compared to the control Tabula sapiens vasculature without plaque lesion. We selected the DEGs with a P-adjusted value ≤ 0.05 and an average $|\log_2\text{Foldchange}| \geq 0.5$.

Network analysis

Empirical network and random network generation

The human protein–protein interaction network (PIN) on which the network propagation was performed was downloaded from IntAct (release 234, last update May 2021) (Kerrien *et al*, 2012; Orchard *et al*, 2014). Kinase-kinase and kinase-substrate relationships from PhosphoSitePlus (last updated May 2021) (Hornbeck *et al*, 2015), OmniPath (last release May 2021) (Türei *et al*, 2016) and SIGNOR 2.0 (Surdo *et al*, 2017) (last release May 2021) (Surdo *et al*, 2017) were included into the PIN. Only proteins annotated in Swiss-Prot (UniProt Consortium, 2021) and with at least one GO term (Thomas *et al*, 2022) (last release May 2021) were retained. The final PIN comprised 16,407 nodes and 238,035 edges. PIN edge weights were modelled according to the Topological Clustering Semantic Similarity (Jain & Bader, 2010) and calculated using the Semantic Measure Library (Harispe *et al*, 2014). To associate a P-value to each network node, we generated 1,000 random networks with the configuration model available in the igraph library (method = vl). Because the configuration model creates new random interactions, the edge weights were updated accordingly.

Each network was corrected for the hub bias according to the following equation:

$$w_{ij} = \frac{w_{ij}}{\sqrt{d_i d_j}}$$

where W_{ij} indicates the edge weight (i.e., semantic similarity) and d_i and d_j represent the weighted degree of node i and node j.

Empirical network propagation

We employed the RWR algorithm (Tong *et al*, 2008) through the personalised PageRank function available with python igraph (http://igraph.org). We ran RWR using as seed nodes anti-ED genes

© 2023 The Authors

(31 seed nodes in total), pro-ED genes (26 seed nodes in total) and both anti-ED and pro-ED all together (57 seed nodes in total) respectively to mimic the effect of each categories of ED on the PIN. To each seed node we associated the value extracted from the aggregated rank analysis to rank the importance of each seed during the propagation. To assess the significance of the nodes, we repeated the procedure above using the same partition but against the 1,000 random networks generated (see "empirical and random network generation"). We then estimated the *P*-value for each node of the PIN and each partition separately using the following formula:

$$P - value = 1 - \frac{\{I \mid RWR_{empirical} > RWR_{random}\}}{1,000}$$

where I is the indicator function, and $RWR_{empirical}$ and RWR_{random} refer to the RWR scores assigned to the empirical PIN and the random networks, respectively. Only nodes with a $P < 0.01$ were selected and isolated nodes were discarded.

Enrichment analysis

We performed an enrichment analysis of BP for the 356 DEGs, differentially expressed in at least one condition under the common 6,630 peaks and the 176, and 167 nodes representing the pro- and anti-ED disease network using the clusterProfiler package (Yu *et al*, 2012). We used over-representation analysis (Boyle *et al*, 2004) to identify biological processes from GO and Disease enriched terms from DisGeNET (Piñero *et al*, 2019) associated with these nodes. Next, for both networks we performed a Drug Target Set Enrichment Analysis (DTSEA), using the respective R package (Su *et al*, 2023), to identify the available drugs and diseases according to each network. We performed semantic similarity analysis among the nodes of each network and compared their distribution by Wilcoxon test. For the pro- and anti-ED, and combined network, we performed a hypergeometric test to confirm whether our networks were enriched in ECs from atherosclerotic plaques. We considered the significance cut-off of the enrichments to be $P < 0.05$.

Data availability

All data and analysis results are available as EV datasets, Source Data. Source Data for EV and Appendix Figures are available in Biostudies: S-BSST1216 (https://www.ebi.ac.uk/biostudies/studies/S-BSST1216). Raw images can be found in Biostudies: S-BIAD862 (https://www.ebi.ac.uk/biostudies/studies/S-BIAD862). The code used in this study for the network analysis is available in https://zenodo.org/record/8215467.

Expanded View for this article is available online.

Acknowledgements

IPS received a fellowship from the Coordenação de Aperfeiçoamento de Pessoal de Nível Superior (CAPES 88882.328126/2019-01) to visit EMBL-EBI. JEK is supported by grants from the Fundação de Amparo à Pesquisa do Estado de São Paulo (FAPESP-INCT—20214/50889-7 and 2013/17368-0); Unrestricted grant from FOXCONN Brazil; and the Conselho Nacional de Desenvolvimento Científico e Tecnológico (CNPq, INCT 465586/2014-7 and 309179/2013-0). EP, GG and IPS were supported by funding provided by EMBL-EBI.

Author contributions

Iguaracy Pinheiro-de-Sousa: Conceptualization; data curation; formal analysis; validation; methodology; writing – original draft; writing – review and editing. **Miriam Helena Fonseca-Alaniz:** Validation; methodology; writing – review and editing. **Girolamo Giudice:** Formal analysis; methodology; writing – original draft; writing – review and editing. **Iuri Cordeiro Valadão:** Validation; methodology. **Silvestre Massimo Modestia:** Validation. **Sarah Viana Mattioli:** Validation. **Ricardo Rosa Junior:** Formal analysis. **Lykourgos-Panagiotis Zalmas:** Validation. **Yun Fang:** Data curation. **Evangelia Petsalaki:** Conceptualization; resources; supervision; funding acquisition; methodology; project administration; writing – review and editing. **José Eduardo Krieger:** Conceptualization; resources; supervision; funding acquisition; project administration; writing – review and editing.

Disclosure and competing interests statement

The authors declare that they have no conflict of interest.

References

Abe H, Hiroaki S, Norihiko T (2017) The roles of hypoxia signaling in the pathogenesis of cardiovascular diseases. *J Atheroscler Thromb* 1: 884–894

Alsaigh T, Evans D, Frankel D, Torkamani A (2022a) Decoding the transcriptome of calcified atherosclerotic plaque at single-cell resolution. *Commun Biol* 5: 1084

Alsaigh T, Evans D, Frankel D, Torkamani A (2022b) Gene Expression Omnibus GSE159677 (https://www.ncbi.nlm.nih.gov/geo/query/acc.cgi?acc=GSE159677). [DATASET]

Aman J, Margadant C (2023) Integrin-dependent cell-matrix adhesion in endothelial health and *disease. Circ Res* 132: 355–378

Appelhoffl RJ, Tian YM, Raval RR, Turley H, Harris AL, Pugh CW, Ratcliffe PJ, Gleadle JM (2004) Differential function of the prolyl hydroxylases PHD1, PHD2, and PHD3 in the regulation of hypoxia-inducible factor. *J Biol Chem* 279: 38458–38465

Birukov KG, Leitinger N, Bochkov VN, Garcia JG (2004) Signal transduction pathways activated in human pulmonary endothelial cells by OxPAPC, a bioactive component of oxidized lipoproteins. *Microvasc Res* 67: 18–28

Boulanger CM (2016) Endothelium. *Arterioscler Thromb Vasc Biol* 36: e26–e31

Boyle EI, Shuai W, Jeremy G, Heng J, David BJ, Michael C, Gavin S (2004) GO::TermFinder–open source software for accessing gene ontology information and finding significantly enriched gene ontology terms associated with a list of genes. *Bioinformatics* 20: 3710–3715

Carpenter AE, Jones TR, Lamprecht MR, Clarke C, Kang IH, Friman O, Guertin DA, Chang JH, Lindquist RA, Moffat J *et al* (2006) CellProfiler: image analysis software for identifying and quantifying cell phenotypes. *Genome Biol* 7: R100

Castro-Mondragon JA, Riudavets-Puig R, Rauluseviciute I, Lemma RB, Turchi L, Blanc-Mathieu R, Lucas J, Boddie P, Khan A, Perez NM *et al* (2022) JASPAR 2022: the 9th release of the open-access database of transcription factor binding profiles. *Nucleic Acids Res* 50: D165–D173

Català-Solsona J, Miñano-Molina AJ, Rodríguez-Álvarez J (2021) Nr4a2 transcription factor in hippocampal synaptic plasticity, memory and cognitive dysfunction: a perspective review. *Front Mol Neurosci* 14: 786226

Chan YH, Harith HH, Israf DA, Than CL (2020) Differential regulation of LPS-mediated VE-cadherin disruption in human endothelial cells and the underlying signaling pathways: a mini review. *Front Cell Dev Biol* 7: 280

 © 2023 The Authors

Coutinho AE, Chapman KE (2011) The anti-inflammatory and immunosuppressive effects of glucocorticoids, recent developments and mechanistic insights. *Mol Cell Endocrinol* 335: 2–13

Cyr AR, Huckaby LV, Shiva SS, Zuckerbraun BS (2020) Nitric oxide and endothelial dysfunction. *Crit Care Clin* 36: 307–321

Daiber A, Chlopicki S (2020) Revisiting pharmacology of oxidative stress and endothelial dysfunction in cardiovascular disease: evidence for redox-based therapies. *Free Radic Biol Med* 157: 15–37

Daiber A, Steven S, Weber A, Shuvaev VV, Muzykantov VR, Laher I, Li H, Lamas S, Münzel T (2017) Targeting vascular (endothelial) dysfunction. *Br J Pharmacol* 174: 1591–1619

de Vries MR, Quax PHA (2016) Plaque angiogenesis and its relation to inflammation and atherosclerotic plaque destabilization. *Curr Opin Lipidol* 27: 499–506

Deanfield JE, Halcox JP, Rabelink TJ (2007) Endothelial function and dysfunction: testing and clinical relevance. *Circulation* 115: 1285–1295

Du L, Dong F, Guo L, Hou Y, Yi F, Liu J, Xu D (2015) Interleukin-1β increases permeability and upregulates the expression of vascular endothelial-cadherin in human renal glomerular endothelial cells. *Mol Med Rep* 11: 3708–3714

Fang Y, Wu D, Birukov KG (2019) Mechanosensing and mechanoregulation of endothelial cell functions. *Compr Physiol* 9: 873–904

Gao JH, Yu XH, Tang CK (2019) CXC chemokine ligand 12 (CXCL12) in atherosclerosis: an underlying therapeutic target. *Clin Chim Acta* 495: 538–544

Gencer S, Evans BR, Vorst EPCV, Döring Y, Weber C (2021) Inflammatory chemokines in atherosclerosis. *Cell* 1–26

Godo S, Shimokawa H (2017) Endothelial functions. *Arterioscler Thromb Vasc Biol* 37: e108–e114

Gokce N, Keaney JF, Hunter LM, Watkins MT, Menzoian JO, Vita JA (2002) Risk stratification for postoperative cardiovascular events via noninvasive assessment of endothelial function: a prospective study. *Circulation* 105: 1567–1572

Han H, Cho JW, Lee S, Yun A, Kim H, Bae D, Yang S, Kim CY, Lee M, Kim E *et al* (2018) TRRUST v2: an expanded reference database of human and mouse transcriptional regulatory interactions. *Nucleic Acids Res* D1: D380–D386

Hao Y, Hao S, Andersen-Nissen E, Mauck WM 3rd, Zheng S, Butler A, Lee MJ, Wilk AJ, Darby C, Zagar M *et al* (2021) Integrated analysis of multimodal single-cell data. *Cell* 184: 3573–3587

Hardoon DR, Szedmak S, Shawe-Taylor J (2004) Canonical correlation analysis: an overview with application to learning methods. *Neural Comput* 16: 2639–2664

Harispe S, Ranwez S, Janaqi S, Montmain J (2014) The semantic measures library and toolkit: fast computation of semantic similarity and relatedness using biomedical ontologies. *Bioinformatics* 30: 740–742

Hennigs JK, Matuszcak C, Trepel M, Körbelin J (2021) Vascular endothelial cells: heterogeneity and targeting approaches. *Cell* 10: 2712

Herrmann SM, Whatling C, Brand E, Nicaud V, Gariepy J, Simon A, Evans A, Ruidavets JB, Arveiler D, Luc G *et al* (2000) Polymorphisms of the human matrix gla protein (MGP) gene, vascular calcification, and myocardial infarction. *Arterioscler Thromb Vasc Biol* 20: 2386–2393

Hogan NT, Whalen MB, Stolze LK, Hadeli NK, Lam MT, Springstead JR, Glass CK, Romanoski CE (2017a) Transcriptional networks specifying homeostatic and inflammatory programs of gene expression in human aortic endothelial cells. *Elife* 6: e22536

Hogan NT, Whalen MB, Stolze LK, Hadeli NK, Lam MT, Springstead JR, Glass CK, Romanoski CE (2017b) Gene Expression Omnibus GSE89970 (https://www.ncbi.nlm.nih.gov/geo/query/acc.cgi?acc=GSE89970). [DATASET]

Hornbeck PV, Zhang B, Murray B, Kornhauser JM, Latham V, Skrzypek E (2015) PhosphoSitePlus, 2014: mutations, PTMs and recalibrations. *Nucleic Acids Res* 43: D512–D520

Huynh DTN, Heo KS (2019) Therapeutic targets for endothelial dysfunction in vascular diseases. *Arch Pharm Res* 42: 848–861

Irwin JJ, Tang KG, Young J, Dandarchuluun C, Wong BR, Khurelbaatar M, Moroz YS, Mayfield J, Sayle RA (2020) ZINC20-a free ultralarge-scale chemical database for ligand discovery. *J Chem Inf Model* 60: 6065–6073

Ivan M, Kaelin WG Jr (2017) The EGLN-HIF O2-sensing system: multiple inputs and feedbacks. *Mol Cell* 66: 772–779

Jain S, Bader GD (2010) An improved method for scoring protein-protein interactions using semantic similarity within the gene ontology. *BMC Bioinformatics* 11: 562

Jia J, Ye T, Cui P, Hua Q, Zeng H, Zhao D (2016) AP-1 transcription factor mediates VEGF-induced endothelial cell migration and proliferation. *Microvasc Res* 105: 103–108

Jones RC, The Tabula Sapiens Consortium (2022a) The Tabula Sapiens: a multiple-organ, single-cell transcriptomic atlas of humans. *Science* 376: eabl4896

Jones RC, The Tabula Sapiens Consortium (2022b) Gene Expression Omnibus GSE201333 (https://www.ncbi.nlm.nih.gov/geo/query/acc.cgi). [DATASET]

Kerrien S, Aranda B, Breuza L, Bridge A, Broackes-Carter F, Chen C, Duesbury M, Dumousseau M, Feuermann M, Hinz U *et al* (2012) The IntAct molecular interaction database in 2012. *Nucleic Acids Res* 40: D841–D846

Kim TH, Dekker J (2018) ChIP-Seq. *Cold Spring Harb Protoc* 2018: pdb.prot082644

Kim I, Moon SO, Kim SH, Kim HJ, Koh YS, Koh GY (2001) Vascular endothelial growth factor expression of intercellular adhesion molecule 1 (ICAM-1), vascular cell adhesion molecule 1 (VCAM-1), and E-selectin through nuclear factor-kappa B activation in endothelial cells. *J Biol Chem* 276: 7614–7762

Kolde R, Laur S, Adler P, Vilo J (2012) Robust rank aggregation for gene list integration and meta-analysis. *Bioinformatics* 28: 573–580

Krause MD, Huang R, Wu D, Shentu T, Harrison DL, Whalen MB (2018a) Genetic variant at coronary artery disease and ischemic stroke locus 1p32.2 regulates endothelial responses to hemodynamics. *Proc Natl Acad Sci USA* 115: E11349–E11358

Krause MD, Huang R, Wu D, Shentu T, Harrison DL, Whalen MB (2018b) Zenodo-260122 (https://zenodo.org/records/260122#.XjfnA2j7TIU). [DATASET]

Langmead B, Salzberg SL (2012) Fast gapped-read alignment with Bowtie 2. *Nat Methods* 9: 357–359

Lawrence M, Gentleman R, Carey V (2009) Rtracklayer: an R package for interfacing with genome browsers. *Bioinformatics* 25: 1841–1842

Lawrence M, Huber W, Pagès H, Aboyoun P, Carlson M, Gentleman R, Morgan MT, Carey VJ (2013) Software for computing and annotating genomic ranges. *PLoS Comput Biol* 9: e1003118

Libby P (2021) Targeting inflammatory pathways in cardiovascular disease: the inflammasome, interleukin-1, interleukin-6 and beyond. *Cell* 10: 951

Liu B, Montgomery SB (2020) Identifying causal variants and genes using functional genomics in specialized cell types and contexts. *Hum Genet* 139: 95–102

Liu F, Gore AJ, Wilson JL, Korc M (2014) DUSP1 is a novel target for enhancing pancreatic cancer cell sensitivity to gemcitabine. *PLoS One* 9: e84982

Liu Z, Ruter DL, Quigley K, Tanke NT, Jiang Y, Bautch VL (2021) Single-cell RNA sequencing reveals endothelial cell transcriptome heterogeneity under homeostatic laminar flow. *Arterioscler Thromb Vasc Biol* 41: 2575–2584

© 2023 The Authors

Love MI, Huber W, Anders S (2014) Moderated estimation of fold change and dispersion for RNA-seq data with DESeq2. *Genome Biol* 15: 550

Luo W, Hu H, Chang R, Zhong J, Knabel M, O'Meally R, Cole RN, Pandey A, Semenza GL (2011) Pyruvate kinase M2 is a PHD3-stimulated coactivator for hypoxia-inducible factor 1. *Cell* 145: 732–744

Lüscher TF, Barton M (1997) Biology of the endothelium. *Clin Cardiol* 20: II-3–II-10

MacLeod C, Hadoke PWF, Nixon M (2021) Glucocorticoids: fuelling the fire of atherosclerosis or therapeutic extinguishers? *Int J Mol Sci* 22: 7622

Martínez-González J, Cañes L, Alonso J, Ballester-Servera C, Rodríguez-Sinovas A, Corrales I, Rodríguez C (2021) NR4A3: a key nuclear receptor in vascular biology, cardiovascular remodeling, and beyond. *Int J Mol Sci* 22: 11371

Matsuzawa Y, Sugiyama S, Sumida H, Sugamura K, Nozaki T, Ohba K, Matsubara J, Kurokawa H, Fujisue K, Konishi M *et al* (2013) Peripheral endothelial function and cardiovascular events in high-risk patients. *J Am Heart Assoc* 2: e000426

McQuin C, Goodman A, Chernyshev V, Kamentsky L, Cimini BA, Karhohs KW, Doan M, Ding L, Rafelski SM, Thirstrup D *et al* (2018) CellProfiler 3.0: next-generation image processing for biology. *PLoS Biol* 16: e2005970

Messmer K, Remington MP, Skidmore F, Fishman PS (2007) Induction of tyrosine hydroxylase expression by the transcription factor Pitx3. *Int J Dev Neurosci* 25: 29–37

Mudau M, Genis A, Lochner A, Strijdom H (2012) Endothelial dysfunction: the early predictor of atherosclerosis. *Cardiovasc J Afr* 23: 222–231

Mussbacher M, Salzmann M, Brostjan C, Hoesel B, Schoergenhofer C, Datler H, Hohensinner P, Basílio J, Petzelbauer P, Assinger A *et al* (2019) Cell type specific roles of Nf-Kb linking inflamation and thrombosis. *Front Immunol* 10: 85

Neckář J, Hsu A, Khan MAH, Gross GJ, Nithipatikom K, Cyprová M, Benák D, Hlaváčková M, Sotáková-Kašparová D, Falck JR (2018) Infarct size-limiting effect of epoxyeicosatrienoic acid analog EET-B is mediated by hypoxia-inducible factor-1α via downregulation of prolyl hydroxylase 3. *Am J Physiol Heart Circ Physiol* 315: H1148–H1158

Ochoa D, Hercules A, Carmona M, Suveges D, Gonzalez-Uriarte A, Malangone C, Miranda A, Fumis L, Carvalho-Silva D, Spitzer M *et al* (2021) Open targets platform: supporting systematic drug-target identification and prioritisation. *Nucleic Acids Res* 49: D1302–D1310

Ochoa D, Karim M, Ghoussaini M, Hulcoop DG, McDonagh EM, Dunham I (2022) Human genetics evidence supports two-thirds of the 2021 FDA-approved drugs. *Nat Rev Drug Discov* 21: 551

Oesterle A, Laufs U, Liao JK (2017) Pleiotropic effects of statins on the cardiovascular system. *Circ Res* 120: 229–243

Orchard S, Ammari M, Aranda B, Breuza L, Briganti L, Broackes-Carter F, Campbell NH, Chavali G, Chen C, Del-Toro N *et al* (2014) The MIntAct project-IntAct as a common curation platform for 11 molecular interaction databases. *Nucleic Acids Res* 42: D358–D363

Peiffer V, Sherwin SJ, Weinberg PD (2013) Does low and oscillatory wall shear stress correlate spatially with early atherosclerosis? A systematic review. *Cardiovasc Res* 99: 242–250

Piñero J, Ramírez-Anguita JM, Saüch-Pitarch J, Ronzano F, Centeno E, Sanz F, Furlong LI (2019) The DisGeNET knowledge platform for disease genomics: 2019 update. *Nucleic Acids Res* 48: D845–D855

Pinheiro-de-Sousa I, Fonseca-Alaniz MH, Teixeira SK, Rodrigues MV, Krieger JE (2022) Uncovering emergent phenotypes in endothelial cells by clustering of surrogates of cardiovascular risk factors. *Sci Rep* 12: 1372

Premer C, Kanelidis AJ, Hare JM, Schulman IH (2019) Rethinking endothelial dysfunction as a crucial target in fighting heart failure. *Mayo Clin Proc* 3: 1–13

Putri GH, Anders S, Pyl PT, Pimanda JE, Zanini F (2022) Analysing high-throughput sequencing data in Python with HTSeq 2.0. *Bioinformatics* 38: 2943–2945

Rahtu-Korpela L, Määttä J, Dimova EY, Hörkkö S, Gylling H, Walkinshaw G, Hakkola J, Kivirikko KI, Myllyharju J, Serpi R *et al* (2016) Hypoxia-inducible factor prolyl 4-hydroxylase-2 inhibition protects against development of atherosclerosis. *Arterioscler Thromb Vasc Biol* 36: 608–617

Ridker PM, Rane M (2021) Interleukin-6 signaling and anti-interleukin-6 therapeutics in cardiovascular disease. *Circ Res* 128: 1728–1746

Schächinger V, Britten MB, Zeiher AM (2000) Prognostic impact of coronary vasodilator dysfunction on adverse long-term outcome of coronary heart disease. *Circulation* 101: 1899–1906

Schneider CA, Rasband WS, Eliceiri KW (2012) NIH image to ImageJ: 25 years of image analysis. *Nat Methods* 9: 671–675

Shi F, Sun L, Kaptoge S (2021) Association of beta-2-microglobulin and cardiovascular events and mortality: a systematic review and meta-analysis. *Atherosclerosis* 320: 70–78

Shin N, Baribaud F, Wang K, Yang G, Wynn R, Covington MB, Feldman P, Gallagher KB, Leffet LM, Lo YY *et al* (2009) Pharmacological characterization of INCB3344, a small molecule antagonist of human CCR2. *Biochem Biophys Res Commun* 387: 251–255

Soehnlein O, Libby P (2021) Targeting inflammation in atherosclerosis - from experimental insights to the clinic. *Nat Rev Drug Discov* 20: 589–610

Souilhol C, Serbanovic-Canic J, Fragiadaki M, Chico TJ, Ridger V, Roddie H, Evans PC (2020) Endothelial responses to shear stress in atherosclerosis: a novel role for developmental genes. *Nat Rev Cardiol* 17: 52–63

Stancu C, Sima A (2001) Statins: mechanism of action and effects. *J Cell Mol Med* 5: 378–387

Steinberg D, Witztum JL (2010) History of discovery: oxidized low-density lipoprotein and atherosclerosis. *Arterioscler Thromb Vasc Biol* 30: 2311–2316

Sterling T, Irwin JJ (2015) ZINC 15-ligand discovery for everyone. *J Chem Inf Model* 55: 2324–2337

Su Y, Wu J, Li X, Li J, Zhao X, Pan B, Huang J, Kong Q, Han J (2023) DTSEA: a network-based drug target set enrichment analysis method for drug repurposing against COVID-19. *Comput Biol Med* 159: 106969

Surdo PL, Calderone A, Cesareni G, Perfetto L (2017) SIGNOR: a database of causal relationships between biological entities-a short guide to searching and browsing. *Curr Protoc Bioinformatics* 58: 8.23.1–8.23.16

Thomas PD, Ebert D, Muruganujan A, Mushayahama T, Albou LP, Mi H (2022) PANTHER: making genome-scale phylogenetics accessible to all. *Protein Sci* 31: 8–22

Tong H, Faloutsos C, Pan JY (2008) Random walk with restart: fast solutions and applications. *Knowl Inf Syst* 14: 327–346

Tromans-Coia C, Diaz-Rohrer B, Weisbart E, Stirling DR, Garcia-Fossa F, Senft RA, Hiner MC, de Jesus MB, Eliceiri KW, Cimini BA (2023) CellProfiler plugins—an easy image analysis platform integration for containers and Python tools. *arXiv* https://doi.org/10.48550/arXiv.2306.01915 [PREPRINT]

Türei D, Korcsmáros T, Saez-Rodriguez J (2016) OmniPath: guidelines and gateway for literature-curated signaling pathway resources. *Nat Methods* 13: 966–967

UniProt Consortium (2021) UniProt: the universal protein knowledgebase in 2021. *Nucleic Acids Res* 49: D480–D489

Vogler M, Zieseniss A, Hesse AR, Levent E, Tiburcy M, Heinze E, Burzlaff N, Schley G, Eckardt KU, Willam C *et al* (2015) Pre- and post-conditional inhibition of prolyl-4-hydroxylase domain enzymes protects the heart from an ischemic insult. *Pflugers Arch* 467: 2141–2149

© 2023 The Authors

Wang J, Zhou JY, Kho D, Reiners JJ Jr, Wu GS (2016) Role for DUSP1 (dual-specificity protein phosphatase 1) in the regulation of autophagy. *Autophagy* 12: 1791–1803

Willighagen EL, Waagmeester A, Spjuth O, Ansell P, Williams AJ, Tkachenko V, Hastings J, Chen B, Wild DJ (2013) The ChEMBL database as linked open data. *J Chem* 5: 23

Wirka RC, Wagh D, Paik DT, Pjanic M, Nguyen T, Miller CL, Kundu R, Nagao M, Coller J, Koyano TK *et al* (2019a) Atheroprotective roles of smooth muscle cell phenotypic modulation and the TCF21 disease gene as revealed by single-cell analysis. *Nat Med* 25: 1280–1289

Wirka RC, Wagh D, Paik DT, Pjanic M, Nguyen T, Miller CL, Kundu R, Nagao M, Coller J, Koyano TK *et al* (2019b) Gene Expression Omnibus GSE131780 (https://www.ncbi.nlm.nih.gov/geo/query/acc.cgi?acc=GSE131780). [DATASET]

Xie L, Pi X, Mishra A, Fong G, Peng J, Patterson C (2012) PHD3-dependent hydroxylation of HCLK2 promotes the DNA damage response. *J Clin Investig* 122: 2927–2936

Xu S, Ilyas I, Little PJ, Li H, Kamato D, Zheng X, Luo S, Li Z, Liu P, Han J *et al* (2021) Endothelial dysfunction in atherosclerotic cardiovascular diseases and beyond: from mechanism to pharmacotherapies. *Pharmacol Rev* 73: 924–967

Yoshitomi Y, Ikeda T, Saito-Takatsuji H, Yonekura H (2021) Emerging role of AP-1 transcription factor JunB in angiogenesis and vascular development. *Int J Mol Sci* 22: 2804

Young L, Sung J, Stacey G, Masters JR (2010) Detection of mycoplasma in cell cultures. *Nat Protoc* 5: 929–934

Yu G, Wang LG, Han Y, He QY (2012) ClusterProfiler: an R package for comparing biological themes among gene clusters. *OMICS* 16: 284–287

Yu G, Wang LG, He QY (2015) ChIP Seeker: an R/bioconductor package for ChIP peak annotation, comparison and visualization. *Bioinformatics* 31: 2382–2383

Zhu LJ, Gazin C, Lawson ND, Pagès H, Lin SM, Lapointe DS, Green MR (2010) ChIPpeakAnno: a bioconductor package to annotate ChIP-Seq and ChIP-Chip data. *BMC Bioinformatics* 11: 237

Zhu T, Cao S, Su PC, Patel R, Shah D, Chokshi HB, Szukala R, Johnson ME, Hevener KE (2013) Hit identification and optimization in virtual screening: practical recommendations based on a critical literature analysis. *J Med Chem* 56: 6560–6572

License: This is an open access article under the terms of the Creative Commons Attribution License, which permits use, distribution and reproduction in any medium, provided the original work is properly cited.

Expanded View Figures

Figure EV1. Mimics of CV risk factors OSS, IL-1β, TNF-α and OxPAPC modulate common regulatory regions on the genome.

A Venn diagram of ATAC-seq peaks of all conditions.
B Venn diagram of ChIP-seq peaks of all conditions.
C Venn diagram of the overlapped peaks of ATAC-seq and ChIP-seq.
D Hierarchical clustering profile reveals detailed relationships between all samples under IL-1β, TNF-α, OSS, OxPAPC, basal control and LSS conditions. The heat map of all samples using Euclidean distance as a measured parameter.
E Venn diagram of 356 DEGs identified under the annotated 6,630 peaks.
F Enriched motifs in the centre of enhancer-like regions are shown. The transcription factor (TF) family, motif sequence and enrichment log *P*-values. Enrichment was calculated from a 200 bp sequence, centred on chromatin accessibility.

Source data are available online for this figure.

© 2023 The Authors

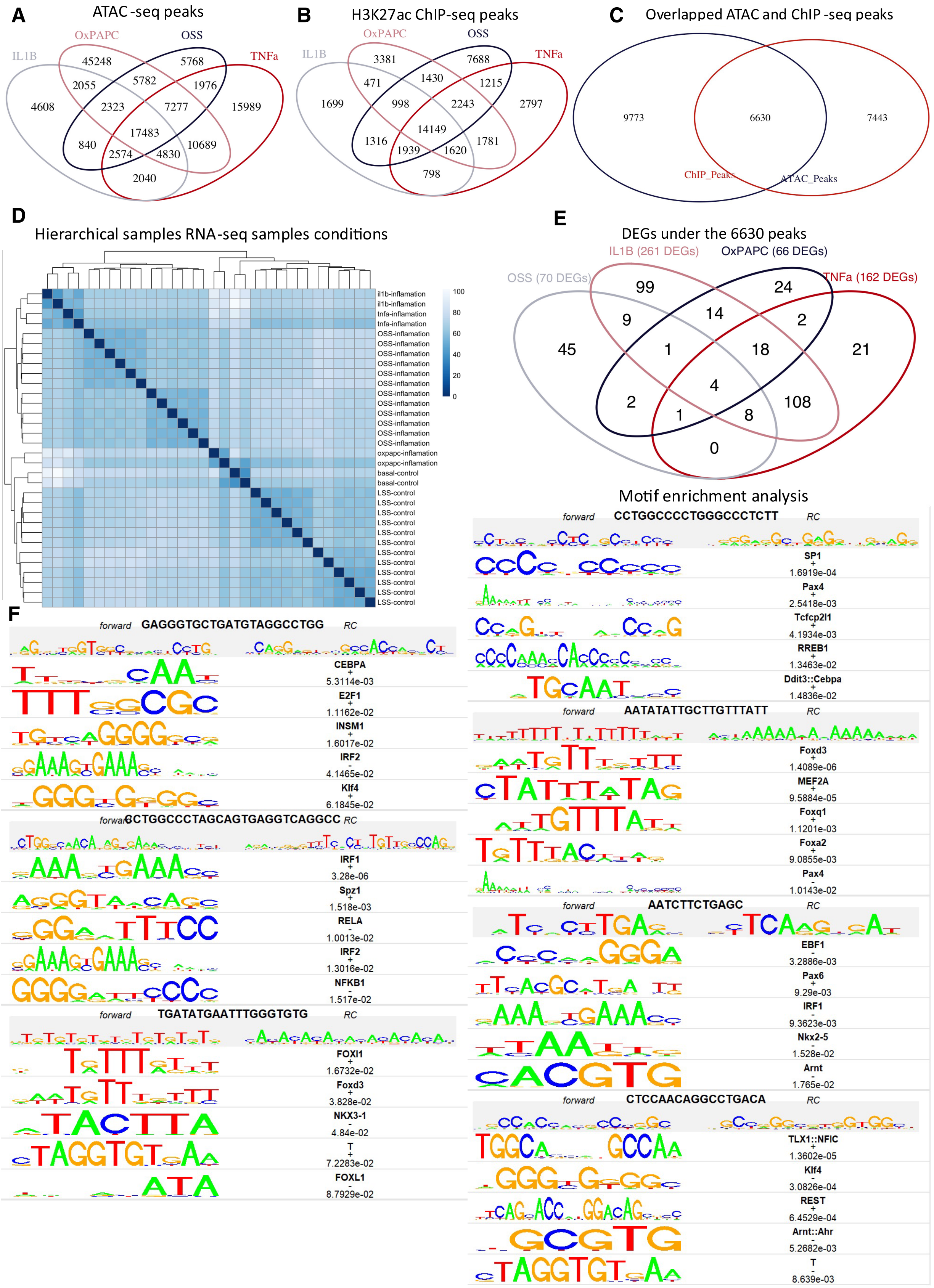
A
ATAC -seq peaks
OxPAPC
OSS
IL1B
TNFa
45248
5768
2055
5782
1976
4608
2323
7277
15989
17483
840
2574
4830
10689
2040
B
H3K27ac ChIP-seq peaks
OxPAPC
OSS
IL1B
TNFa
3381
7688
1430
471
1215
1699
998
2243
2797
14149
1316
1781
1939
1620
798
C
Overlapped ATAC and ChIP -seq peaks
9773
6630
7443
ChIP_Peaks
ATAC_Peaks
D
Hierarchical samples RNA-seq samples conditions
il1b-inflamation
tnfa-inflamation
OSS-inflamation
oxpapc-inflamation
basal-control
LSS-control
100
80
60
40
20
0
E
DEGs under the 6630 peaks
IL1B (261 DEGs)
OxPAPC (66 DEGs)
OSS (70 DEGs)
TNFa (162 DEGs)
99
24
9
14
2
45
1
18
21
4
2
108
1
8
0
Motif enrichment analysis
forward CCTGGCCCCTGGGCCCTCTT RC
SP1 + 1.6919e-04
Pax4 + 2.5418e-03
Tcfcp2l1 + 4.1934e-03
RREB1 + 1.3463e-02
Ddit3::Cebpa + 1.4836e-02
forward AATATATTGCTTGTTTATT RC
Foxd3 + 1.4089e-06
MEF2A + 9.5884e-05
Foxq1 + 1.1201e-03
Foxa2 + 9.0855e-03
Pax4 - 1.0143e-02
forward AATCTTCTGAGC RC
EBF1 - 3.2886e-03
Pax6 - 9.29e-03
IRF1 - 9.3623e-03
Nkx2-5 - 1.528e-02
Arnt - 1.765e-02
forward CTCCAACAGGCCTGACA RC
TLX1::NFIC + 1.3602e-05
Klf4 - 3.0826e-04
REST + 6.4529e-04
Arnt::Ahr - 5.2682e-03
T - 8.639e-03
F
forward GAGGGTGCTGATGTAGGCCTGG RC
CEBPA + 5.3114e-03
E2F1 + 1.1162e-02
INSM1 + 1.6017e-02
IRF2 - 4.1465e-02
Klf4 - 6.1845e-02
forward GCTGGCCCTAGCAGTGAGGTCAGGCC RC
IRF1 + 3.28e-06
Spz1 + 1.518e-03
RELA - 1.0013e-02
IRF2 - 1.3016e-02
NFKB1 - 1.517e-02
forward TGATATGAATTTGGGTGTG RC
FOXI1 + 1.6732e-02
Foxd3 + 3.828e-02
NKX3-1 - 4.84e-02
T + 7.2283e-02
FOXL1 - 8.7929e-02

Figure EV1.

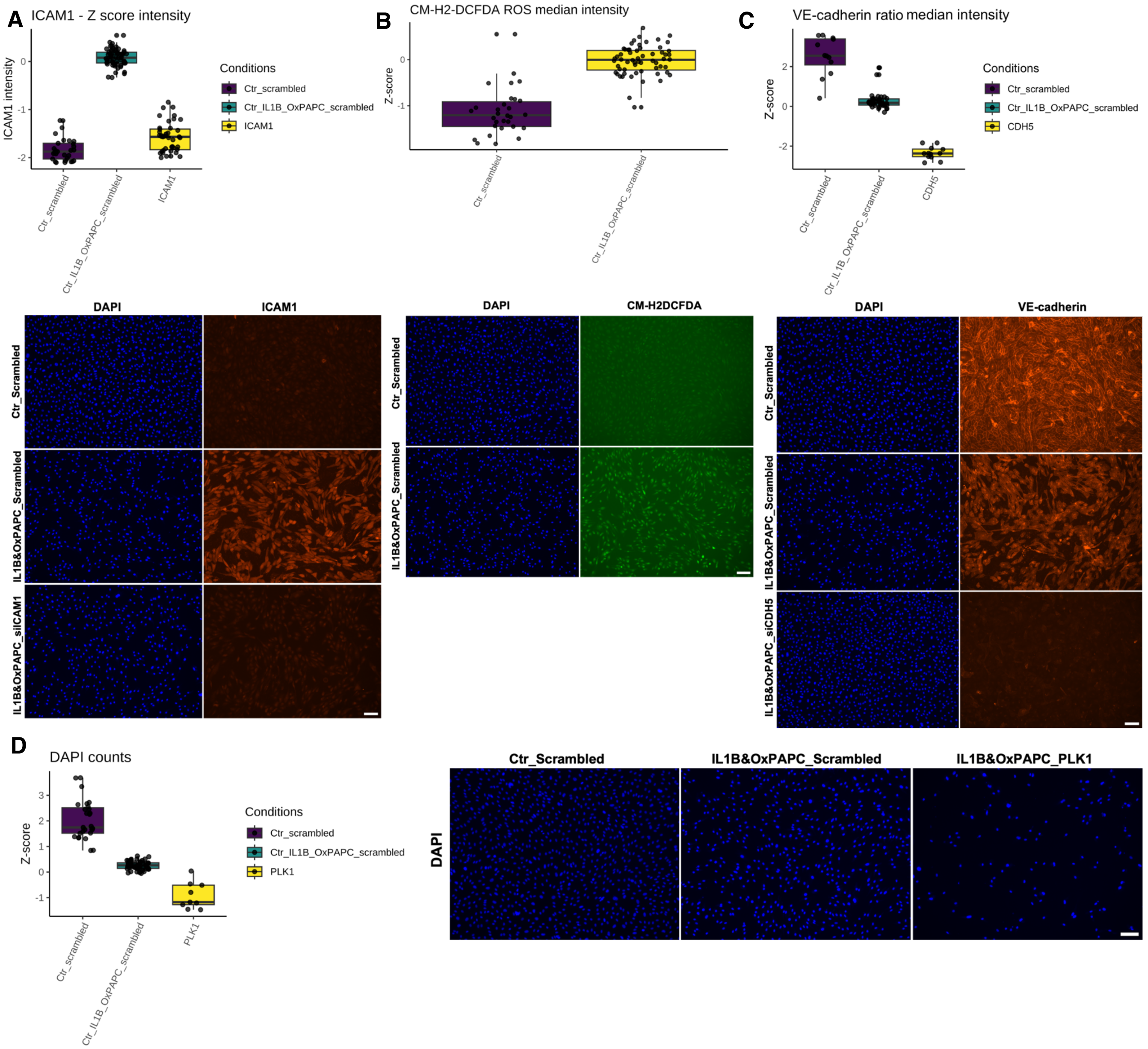

Figure EV2. IL-1β&OxPAPC and siRNA standardisation by immunofluorescence.

A–D Representative fluorescent micrographs (Scale bar = 100 μm) and quantification of (A) ICAM1 intensity, (B) CM-H2DCFDA (ROS indicator) intensity, (C) VE-cadherin intensity and (D) nuclei (DAPI) number in HAEC transfected with Scrambled siRNA or siRNA to ICAM1, CDH5 and PLK1, and treated or not with IL-1β and OxPAPC. The data are represented as a *Z*-score (Materials and Methods). The experiment was repeated 5 times independently (biological replicates), with 4–5 technical replicates from each condition. The representative images of DAPI staining of Ctrl Scrambled in (C) are the same as those used in (A).

Source data are available online for this figure.

© 2023 The Authors

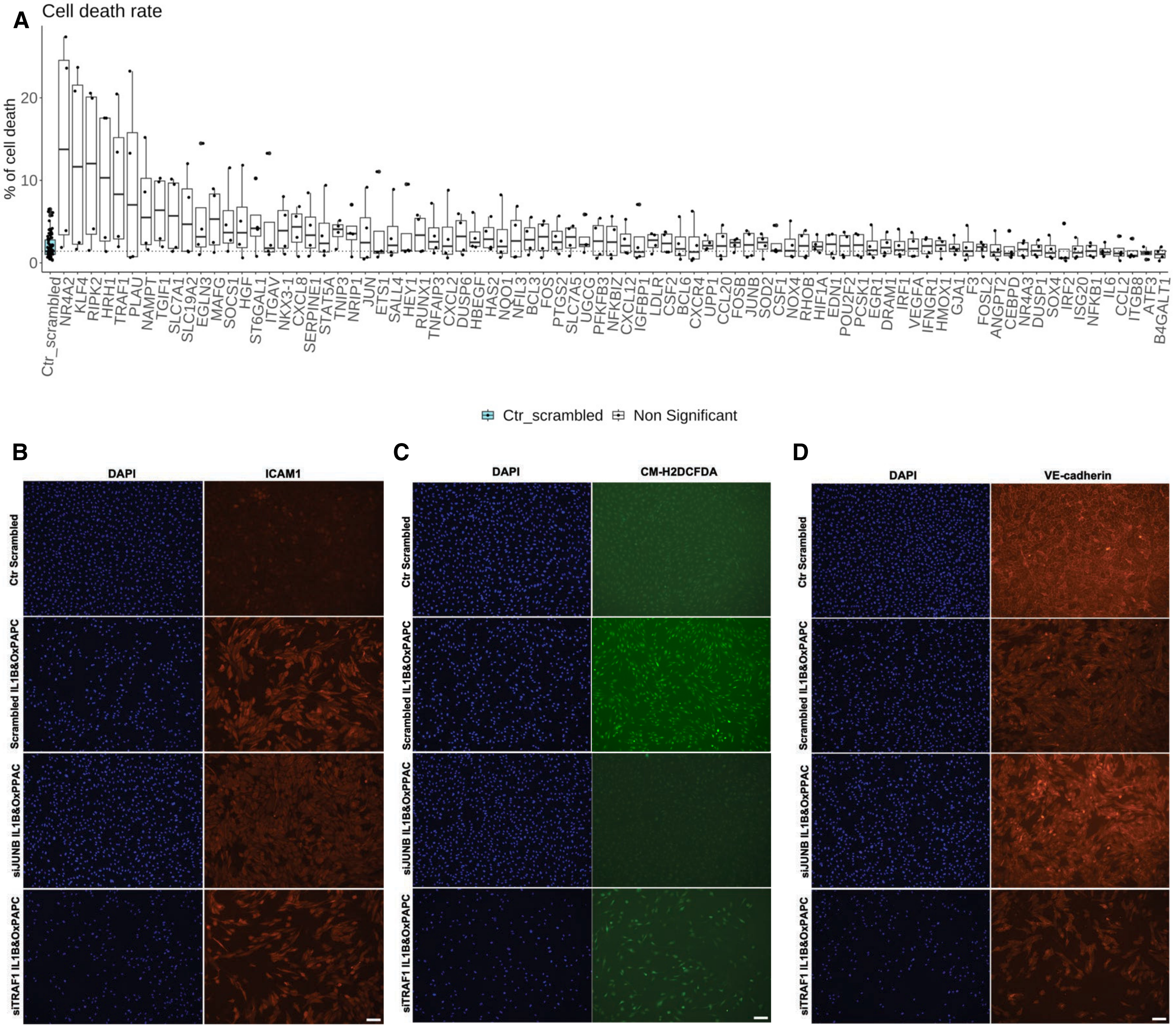

Figure EV3. siRNA transfection and immunofluorescence.

A Percentage of cell death calculated from a cell viability assay (calcein and ethidium staining) in HAEC transfected with Scrambled siRNA or siRNA to 81 genes, without treatment with IL-1β and OxPAPC. *P*-values were computed by multiple pairwise comparisons with the Wilcoxon test followed by BH correction. Data are presented as mean ± standard deviation. The boxplot depicts the median within the 25th and 75th percentiles, which the whisker extends no further than 1.5 × IQR (Interquartile Range). The experiment was repeated 4 times independently (biological replicates), with 8 technical replicates for all controls in the plate each time. All the comparisons were made between HAECs transfected with each of the 81 siRNA versus HAEC transfected with Scrambled siRNA.

B–D Representative fluorescent micrographs (Scale bar = 100 μm) of (A) ICAM1, (B) CM-H2DCFDA (ROS indicator) and (C) VE-cadherin in HAEC transfected with Scrambled siRNA or siRNA to JUNB and TRAF1, and treated or not with IL-1β and OxPAPC. The representative images of DAPI and CM-H2DCFDA staining of Scrambled IL1B&OxPAPC in (C) are the same as those used in Fig EV2B.

Source data are available online for this figure.

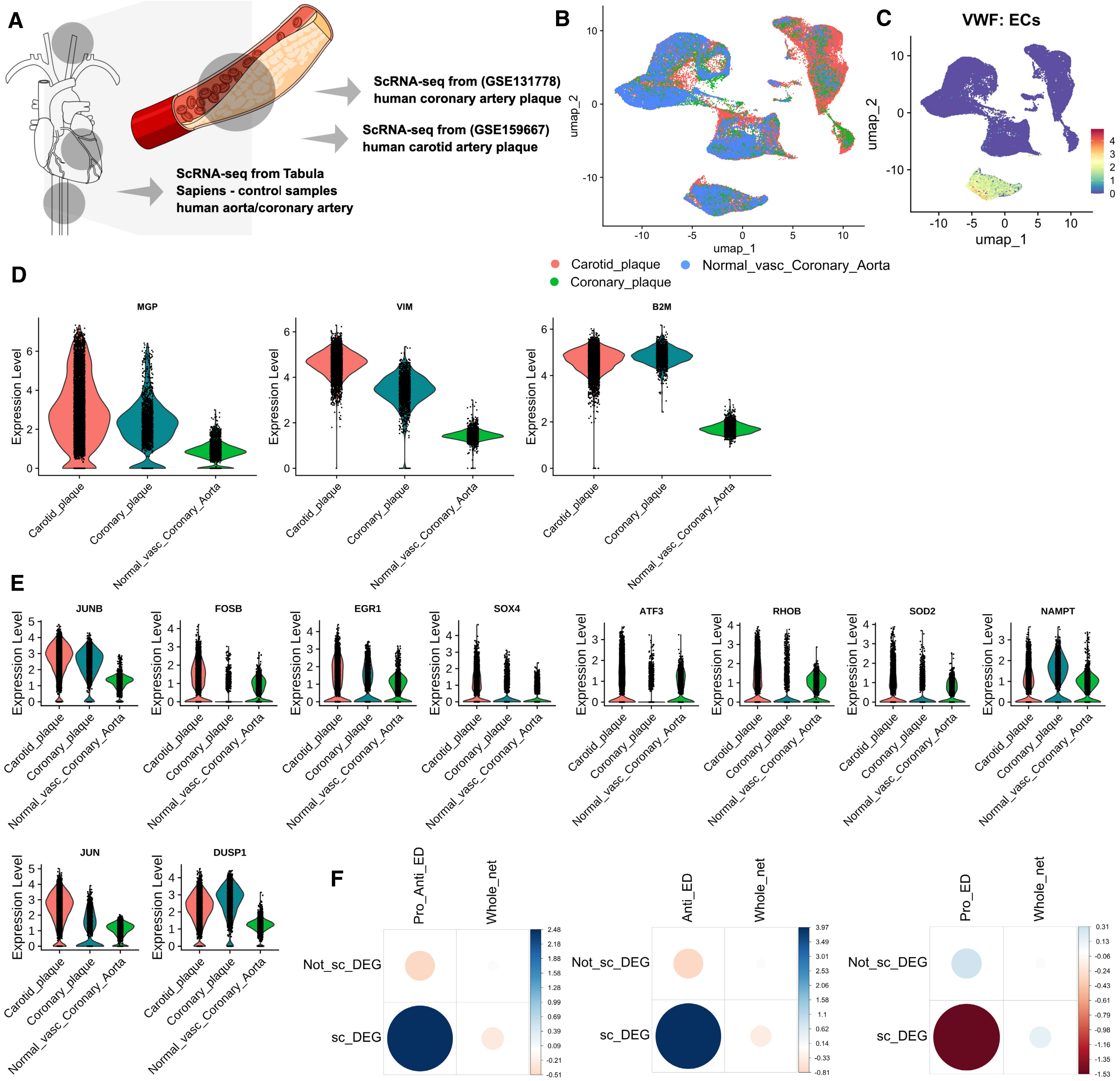

Figure EV4. Validation of the endothelial dysfunction (ED) network with single-cell RNA sequencing (scRNA-seq) datasets obtained from human atherosclerotic plaques.

- A Schematic figure showing the scRNA-seq public data used (Materials and Methods) to validate the ED disease network. The data were composed of two datasets containing atherosclerosis lesions and one control dataset from the Tabula sapiens. The cartoons were created using the Mind the Graph platform (www.mindthegraph.com).
- B Uniform manifold approximation and projection (UMAP) visualisation of the integrated scRNAseq datasets used to validate the ED disease network (n = 69,385 cells).
- C UMAP visualisation of a critical endothelial cell (EC) marker VWF.
- D Violin plot of the three top-upregulated genes in the ECs from atherosclerotic sites compared to the normal vasculature.
- E Violin plot of the TFs differentially expressed *in vitro* (HAECs under the stimuli) and *in vivo* (upregulated in ECs at the atherosclerotic plaque).
- F Hypergeometric test to test enrichment of DEGs from the single cell data in our pro-anti ED network P = 0.002; anti-ED network P = 0.00041; pro-ED network P = 0.171. The plots depict contingency tables for each hypergeometric test. In these tables, values with two decimal places indicate residuals. Positive residuals, suggesting that the observed values were more frequent than expected, are in blue, and negative residuals, suggesting the opposite, are in red.

Source data are available online for this figure.

    © 2023 The Authors

Figure EV5. Network propagation from knockdown genes.

A ED protein–protein interaction network (PIN) disease network generated by combining the 26 pro-ED and 31 anti-ED genes and the random-walk-with-restart (RWR) algorithm. The resulting network comprises 216 nodes and 317 edges. Colour reflects the average log_2 fold change in the gene expression of these genes across the datasets integrated in the study. The size of the node represents the degree, i.e., number of interacting partners. The colour of the outline of the nodes reflects whether it was in the anti- or pro-ED gene list where relevant.

B Distribution of semantic similarity values for all nodes in the pro-ED network and in the anti-ED network.

C Distribution of number of BP terms for the nodes in the anti- and pro-ED networks.

D GO enrichment analysis for Biological Processes for the pro-ED network and its 26 pro-ED seed nodes genes (adjusted P-value $\leq$ 0.05)

E GO enrichment analysis for Biological Processes for the anti-ED network and its 31 anti-ED seed nodes genes (adjusted P-value $\leq$ 0.05).

Source data are available online for this figure.

© 2023 The Authors

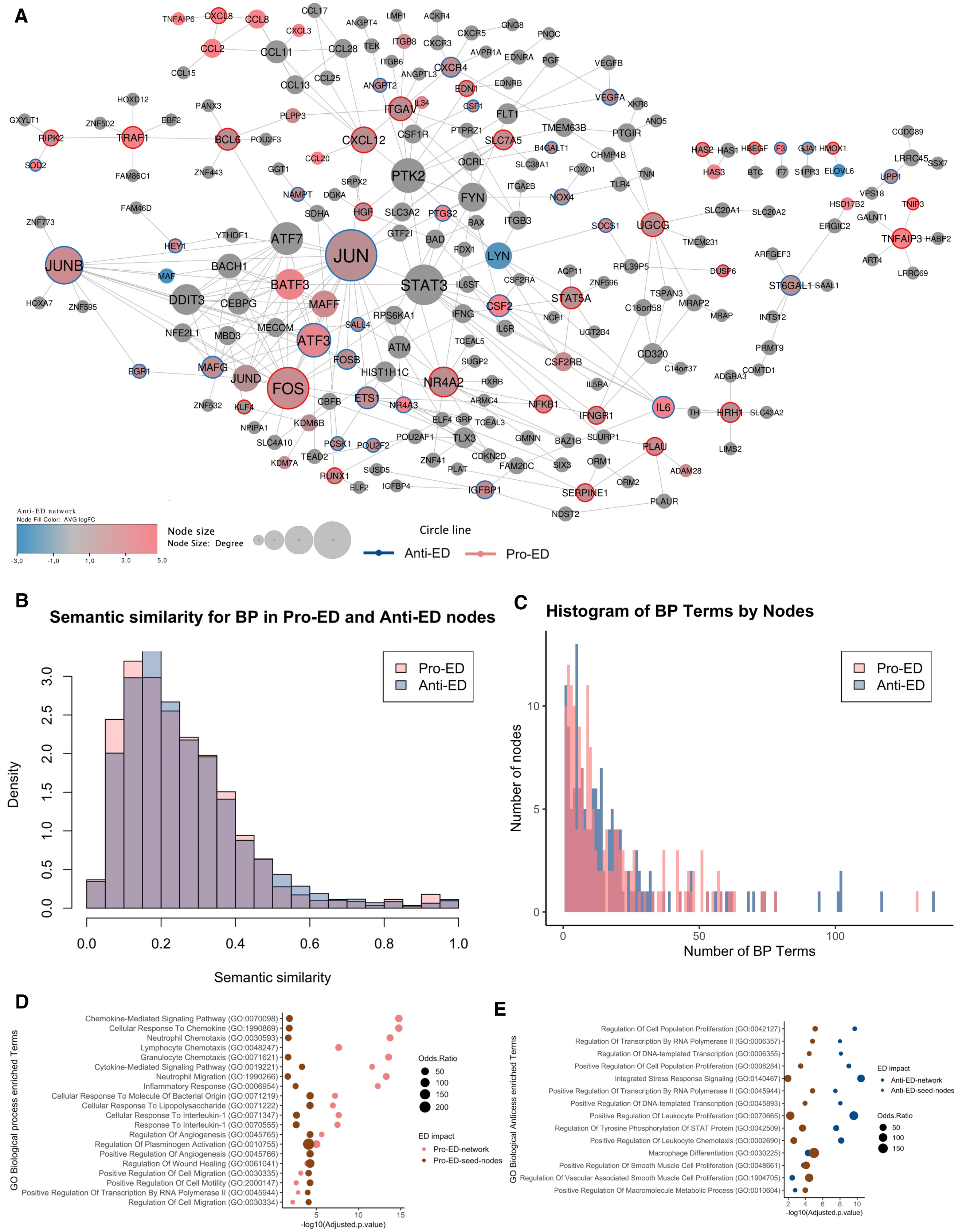

Figure EV5.

 © 2023 The Authors

Figure EV6. Drug treatment high content screen data.

A, B (A) Representative fluorescent micrographs (Scale bar = 50 μm) and (B) quantification of percentage of cell death calculated from a cell viability assay (calcein and ethidium staining) in HAEC treated with the 17 drugs at 10^{-8} and 10^{-6} M, without IL-1β and OxPAPC. Starving treatment for 24 h was used as a cell death positive control. Data are presented as mean ± standard deviation. The experiment was repeated 3 times independently (biological replicates), with 1 technical replicate from each condition. *P*-values were computed by multiple pairwise comparisons with the Wilcoxon test followed by BH correction. The conditions were compared to starving.

C–F Quantification of (C) ICAM1 intensity, (D) CM-H2DCFDA (ROS indicator) intensity, (E) VE-cadherin intensity and, (F) nuclei (DAPI) number in HAEC treated with IL-1β and OxPAPC alone (DMSO solvent), or in combination with inhibitor drugs at 10^{-6} M or 10^{-8} M. The data are represented as a *Z*-score (Materials and Methods). All experiments were repeated 2 times independently (biological replicates), with 4–5 technical replicates from each condition. Only significant results (P-value < 0.05) are shown. The boxplot depicts the median within the 25th and 75th percentiles, which the whisker extends no further than 1.5 × IQR (Interquartile Range).

G Aggregated rank analysis combining the siRNA and drugs screening exacerbating (red) and reducing (blue) ED phenotypes upon treatment. The first ranking, from left to right, rates genes by pro-ED ranking scores (Figs 2F and 4B). These are pro-ED genes. The second ranking is for anti-ED genes, with anti-ED ranking score (Materials and Methods). Both are displayed on the same plot, represented by the 2 dots per target.

Source data are available online for this figure.

© 2023 The Authors

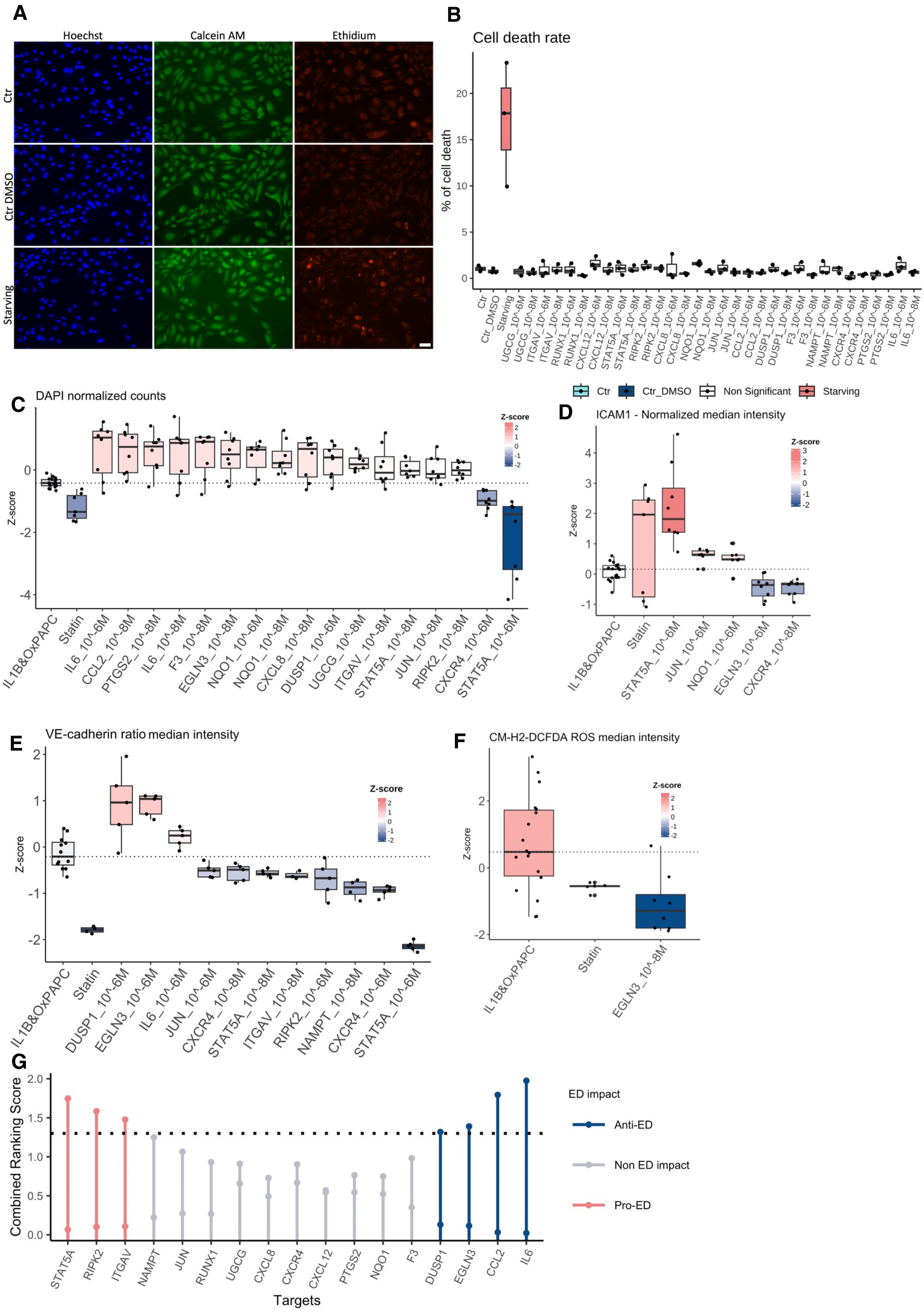

Figure EV6.

© 2023 The Authors

Integrated Systems Biology Approach Identifies Gene Targets for Endothelial Dysfunction

Iguaracy Pinheiro-de-Sousa[1,2+], Miriam Helena Fonseca-Alaniz[1+], Girolamo Giudice[2], Iuri Cordeiro Valadão[1], Silvestre Massimo Modestia[1], Sarah Viana Mattioli[1,3], Ricardo Rosa Junior[1], Lykourgos-Panagiotis Zalmas[4,5], Yun Fang[6], Evangelia Petsalaki[2*], José Eduardo Krieger[1*]

[1]Laboratory of Genetics and Molecular Cardiology, Heart Institute (InCor)/University of São Paulo Medical School, São Paulo, SP, Brazil.
[2]European Molecular Biology Laboratory, European Bioinformatics Institute, Hinxton, UK
[3]Department of Biophysics and Pharmacology, Institute of Biosciences of Botucatu, Universidade Estadual Paulista, Botucatu, São Paulo, Brazil
[4]Wellcome Trust Sanger Institute, Wellcome Trust Genome Campus, Cambridge, CB10 1SA, UK
[5]Open Targets, Wellcome Genome Campus, Hinxton, Cambridge, CB10 1SA, UK.
[6]Department of Medicine, University of Chicago, Chicago, IL, United States

+ these authors contributed equally to the study

* correspondence should be addressed to Evangelia Petsalaki: petsalaki@ebi.ac.uk and Jose E. Krieger: j.krieger@hc.fm.usp.br

Appendix Files

Table of Contents

Appendix Figure S1. Molidustat and EGLN1-3 siRNA effects. (Page 2)

Appendix Figure S2. Extended images of all immunoblotting membranes used in this study. (Page 4)

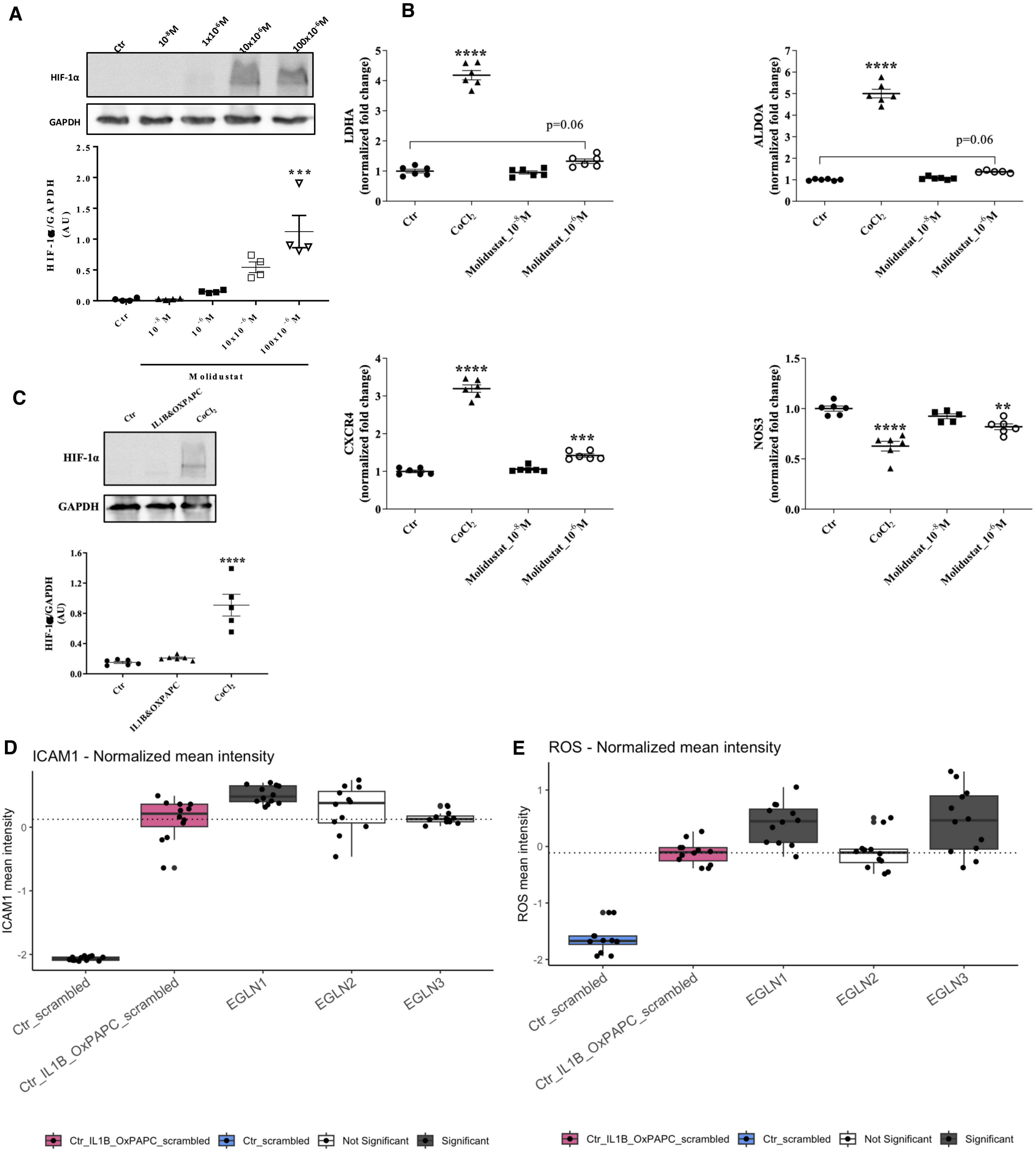
A
Ctr
10-8M
1x10-6M
10x10-6M
100x10-6M
HIF-1α
GAPDH
HIF-1α/GAPDH (AU)
***
Molidustat
B
LDHA (normalized fold change)
****
p=0.06
Ctr
CoCl2
Molidustat_10-8M
Molidustat_10-6M
ALDOA (normalized fold change)
CXCR4 (normalized fold change)
NOS3 (normalized fold change)
**
C
IL1B&OXPAPC
D
ICAM1 - Normalized mean intensity
ICAM1 mean intensity
Ctr_scrambled
Ctr_IL1B_OxPAPC_scrambled
EGLN1
EGLN2
EGLN3
Not Significant
Significant
E
ROS - Normalized mean intensity
ROS mean intensity

Appendix Figure S1. Molidustat and EGLN1-3 siRNA effects.

(A) *Representative image of immunoblotting membrane and quantification of HIF-1α protein expression in HAEC treated with Molidustat with a range of doses from 10^{-8}M to $100x10^{-6}$M for 48h. Data are presented as mean ± mean standard error. ***$p<0.001$ vs. Ctr. GAPDH was used as a housekeeping protein. The experiment was repeated 2 times independently (biological replicates), with 2 technical replicates from each condition.*

(B) *HAEC were treated with Molidustat at 10^{-8}M and 10^{-6}M, or Cobalt Chloride ($CoCl_2$, 300µM) for 48h and gene expression of Lactate Dehydrogenase A (LDHA), Aldolase Fructose-Bisphosphate A (ALDOA), C-X-C Motif Chemokine Receptor 4 (CXCR4) and Nitric Oxide Synthase 3 (NOS3) were evaluated by qPCR. Data are presented as mean ± mean standard error. ****$p<0.0001$, ***$p<0.001$, **$p<0.01$ vs. Ctr. The experiment was repeated 2 times independently (biological replicates), with 3 technical replicates from each condition.*

(C) *Representative image of immunoblotting membrane and quantification of HIF-1α protein expression in HAEC treated with IL-1β and OxPAPC or Cobalt Chloride ($CoCl_2$, 300µM) for 48h. Data are presented as mean ± mean standard error. ****$p<0.0001$ vs. Ctr. GAPDH was used as a housekeeping protein. The experiment was repeated 2 times independently (biological replicates), with 3 technical replicates from each condition.*

(D-E) *Quantification of (D) ICAM intensity and (E) CM-H2DCFDA (ROS indicator) intensity in HAEC transfected with Scrambled siRNA or siRNA to EGLN1, EGLN2 and EGLN3, and treated or not with IL-1β and OxPAPC. The data are represented as a Z-score (Material and Methods). The boxplot depicts the median within the 25th and 75th percentiles, which the whisker extends no further than 1.5 * IQR (Interquartile Range). p-values were computed by multiple pairwise comparisons with the Wilcoxon test followed by BH correction. The comparisons were made between HAECs transfected with EGLN1-3 siRNA versus HAEC transfected with Scrambled siRNA and treated with IL-1β and OxPAPC. The experiment was repeated 4 times independently (biological replicates), with 4 technical replicates from each condition.*

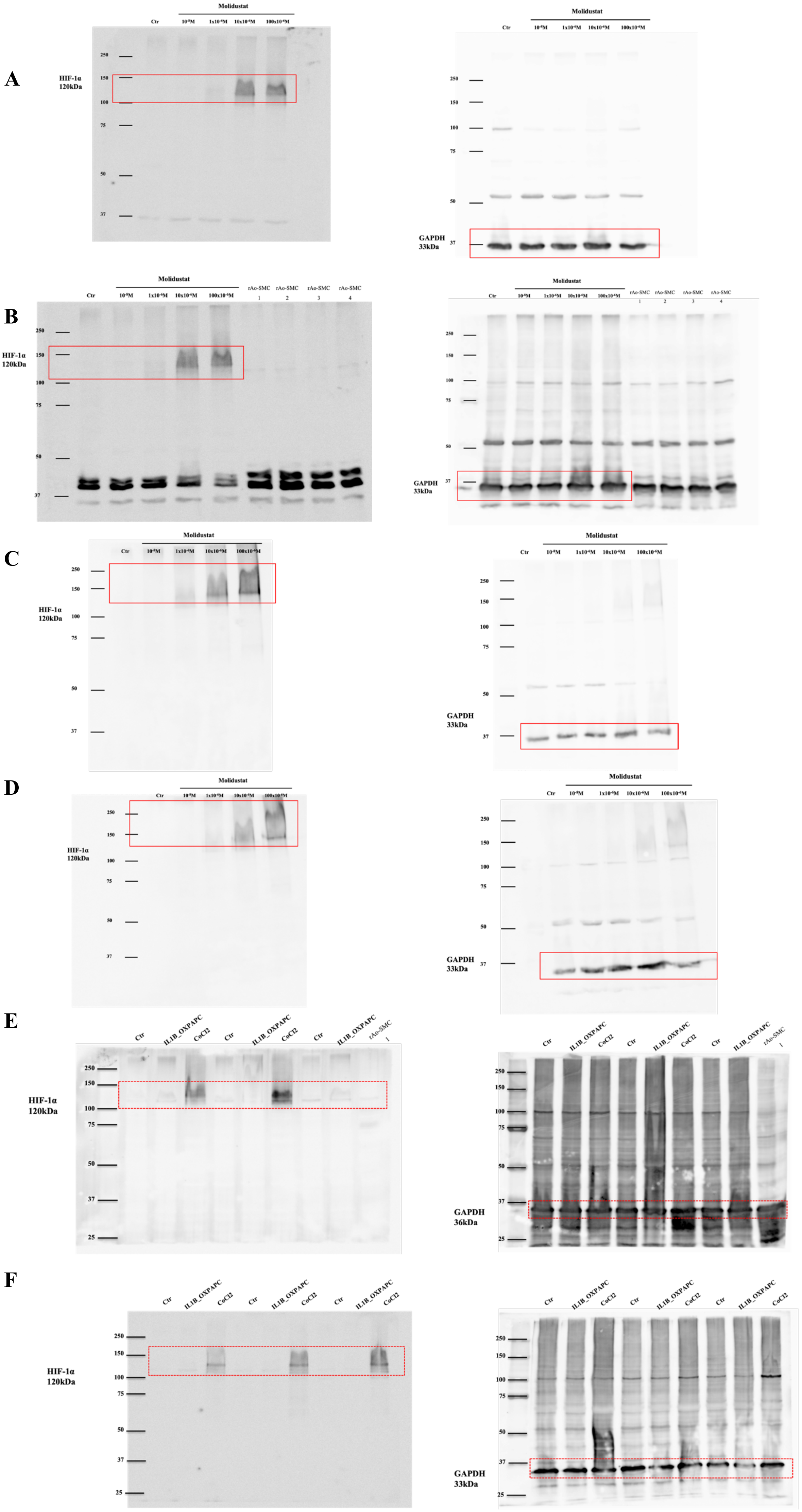
A
B
C
D
E
F
Molidustat
Ctr
HIF-1α 120kDa
GAPDH 33kDa
GAPDH 36kDa
rAo-SMC
IL1B_OXPAPC
CoCl2
250
150
100
75
50
37
25

Appendix Figure S2. Extended images of all immunoblotting membranes used in this study.

(A-D) *Images relative to Appendix Figure S1A.*

(E-F) *Images relative to Appendix Figure S1C. rAo-SMC= rat aorta smooth muscle cell*

.
