# Supplementary material for: Integrated systems biology approach identifies gene targets for endothelial dysfunction
Source: Mol Syst Biol. 2023 Nov 30;19(12):e11462. doi: 10.15252/msb.202211462 (PMC10698507; doi:10.15252/msb.202211462)
Supplement: Supplementary file 12 — Source Data for Figure 1 [file MSB-19-e11462-s008.zip › Source_data_figure_1/README.rtf]

Files to reproduce figure 1This repository contains 3 R objects to plot Fig 1A, B, C and E. And one table with all DEGs identified under the 6630 peaks from ATAC-seq and ChIP-seq
