# Supplementary material for: Integrated systems biology approach identifies gene targets for endothelial dysfunction
Source: Mol Syst Biol. 2023 Nov 30;19(12):e11462. doi: 10.15252/msb.202211462 (PMC10698507; doi:10.15252/msb.202211462)
Supplement: Supplementary file 13 — Source Data for Figure 2 [file MSB-19-e11462-s014.zip › Source_data_figure_2/README.rtf]

Files to reproduce figure 2This repository contains 6 tables. 4 for all markers with the z-score o reproduce the heatmap and boxplots Figure 2A-E. And two tables to reproduce the ranking plot figure 2F.
