# Supplementary material for: Integrated systems biology approach identifies gene targets for endothelial dysfunction
Source: Mol Syst Biol. 2023 Nov 30;19(12):e11462. doi: 10.15252/msb.202211462 (PMC10698507; doi:10.15252/msb.202211462)
Supplement: Supplementary file 14 — Source Data for Figure 3 [file MSB-19-e11462-s011.zip › Source_data_figure_3/README.rtf]

Files to reproduce figure 4This repository contains 2 tables. The tables contain the nodes from pro- and anti-ED generate the networks (Fig 4A and B), and to used the nodes for enrichment analysis for BP, Diseases and Drugs enrichment (Figure 4C and D).
