# Supplementary material for: Integrated systems biology approach identifies gene targets for endothelial dysfunction
Source: Mol Syst Biol. 2023 Nov 30;19(12):e11462. doi: 10.15252/msb.202211462 (PMC10698507; doi:10.15252/msb.202211462)
Supplement: Supplementary file 15 — Source Data for Figure 4 [file MSB-19-e11462-s010.zip › Source_data_figure_4/README.rtf]

Files to reproduce figure 3This repository contains 6 tables. 4 for all markers with the z-score o reproduce the heatmap (Figure 3A) and boxplots (Figure EV4C-F). And two tables to reproduce the ranking plot figure 3B.
