# Supplementary material for: Integrated systems biology approach identifies gene targets for endothelial dysfunction
Source: Mol Syst Biol. 2023 Nov 30;19(12):e11462. doi: 10.15252/msb.202211462 (PMC10698507; doi:10.15252/msb.202211462)
Supplement: Supplementary file 16 [file MSB-19-e11462-s003.pdf]

molecular systems biology

Integrated Systems Biology Approach Identifies Gene Targets for Endothelial Dysfunction

Iguaracy Pinheiro-de-Sousa, Miriam Fonseca-Alaniz, Girolamo Giudice, Iuri Valadão, Silvestre Modestia, Sarah Mattioli, Ricardo Junior, Lykourgos-Panagiotis Zalmas, Yun Fang, Evangelia Petsalaki, and Jose Krieger
DOI: 10.15252/msb.202211462

Corresponding author(s): Evangelia Petsalaki (petsalaki@ebi.ac.uk)

Review Timeline:

Submission Date:	18th Nov 22
Editorial Decision:	11th Jan 23
Revision Received:	5th Aug 23
Editorial Decision:	22nd Sep 23
Revision Received:	20th Oct 23
Accepted:	23rd Oct 23

Editor: Maria Polychronidou

Transaction Report:

(Note: With the exception of the correction of typographical or spelling errors that could be a source of ambiguity, letters and reports are not edited. Depending on transfer agreements, referee reports obtained elsewhere may or may not be included in this compilation. Referee reports are anonymous unless the Referee chooses to sign their reports.)

11th Jan 2023

RE: MSB-2022-11462, In silico network-based screening reveals candidates for endothelial dysfunction therapy

Dear Evangelia,

Thank you again for submitting your work to Molecular Systems Biology. My apologies for the delay in sending you a decision on your manuscript, which was due to the end of the year holidays. We have now heard back from the three reviewers who agreed to evaluate your study. As you will see below, the reviewers raise a series of concerns, which preclude the publication of the study in its current form. However, as the reviewers did have positive words for the goals of the study and the potential value of the integrative approach, we have decided to give you a chance to perform a major revision and address the issues raised by the reviewers.

Without repeating all the comments listed below, some of the more fundamental issues are the following:

- Further analyses and controls are needed to support the computational part of the study more rigorously. Moreover, several clarifications need to be provided regarding how the computational analyses were performed.

- Reviewers #2 and #3 point out that the validation of the selected target (EGLN) does not seem sufficiently conclusive. During our cross-commenting process, in which the referees are given the chance to make additional comments based on the other reports, reviewer #1 agreed that this is a critical point and that additional experiments with complementary genetic perturbations or a more specific inhibitor are needed.

- The reviewers also felt that the validation of a single target seems somewhat limited. They mention that additional validations of more targets would significantly increase the impact of the study and would increase the confidence in the performance of the computational approach.

All issues raised by the reviewers need to be satisfactorily addressed as they are related to the conclusiveness of the main findings. The reviewers make constructive suggestions on how to improve the study. As you may already know, our editorial policy allows in principle a single round of major revision. It is therefore essential to provide responses to the reviewers' comments that are as complete as possible. Given that the required revisions are quite substantive, we would understand if you would choose to not perform all these additional experiments and would decide to submit the study elsewhere. If you decide to revise and re-submit to MSB and would like to discuss your revision plan please feel free to get in touch.

On a more editorial level, we would ask you to address the following points:

- Please provide a .doc version of the manuscript text (including legends for the main figures) and individual production quality figure files for the main Figures (one file per figure).

- We have replaced Supplementary Information by the Expanded View (EV format). In this case, all additional figures and Tables can be included in a PDF called Appendix. Appendix figures and Tables should be labeled and called out as: "Appendix Figure S1, Appendix Figure S2... Appendix Table S1..." etc. Each legend should be below the corresponding Figure/Table in the Appendix. Please include a Table of Contents in the beginning of the Appendix. For detailed instructions regarding expanded view please refer to our Author Guidelines: .

- Tables S1-S4 need to be provided as Datasets EV1-EV4. Please provide one file per EV Dataset. Each .xls file should contain a brief description of the Dataset in a separate tab.

- Supplementary Table 5 (and other short/simple tables, should you wish to include any) can remain in the Appendix, but it needs to be labeled Appendix Table S1.

- Please provide a "standfirst text" summarizing the study in one or two sentences (approximately 250 characters), three to four "bullet points" highlighting the main findings and a "synopsis image" (550px width and max 400px height, jpeg format) to highlight the paper on our homepage.

- Please include 5 keywords.

- All Materials and Methods need to be described in the main text. We would encourage you to use 'Structured Methods', our new Materials and Methods format. According to this format, the Material and Methods section should include a Reagents and Tools Table (listing key reagents, experimental models, software and relevant equipment and including their sources and

relevant identifiers) followed by a Methods and Protocols section in which we encourage the authors to describe their methods using a step-by-step protocol format with bullet points, to facilitate the adoption of the methodologies across labs. More information on how to adhere to this format as well as downloadable templates (.doc or .xls) for the Reagents and Tools Table can be found in our author guidelines: . An example of a Method paper with Structured Methods can be found here:

- Please include a "Disclosure & Competing Interests Statement".

- Please include a Data availability section describing how the data, code etc. have been made available. This section needs to be formatted according to the example below:
The datasets and computer code produced in this study are available in the following databases:
- Chip-Seq data: Gene Expression Omnibus GSE46748 (https://www.ncbi.nlm.nih.gov/geo/query/acc.cgi?acc=GSE46748)
- Modeling computer scripts: GitHub (https://github.com/SysBioChalmers/GECKO/releases/tag/v1.0)
- [data type]: [full name of the resource] [accession number/identifier] ([doi or URL or identifiers.org/DATABASE:ACCESSION])

- For data quantification: please specify the name of the statistical test used to generate error bars and P values, the number (n) of independent experiments (specify technical or biological replicates) underlying each data point and the test used to calculate p-values in each figure legend. The figure legends should contain a basic description of n, P and the test applied. Graphs must include a description of the bars and the error bars (s.d., s.e.m.).

- Molecular Systems Biology supports formal data citations in the Reference list, to cite previously published datasets. In addition to citing the original papers that reported the data, we encourage you to also cite the relevant datasets directly in the Reference list. In the text, references to datasets are included as "Data ref: Smith et al, 2001" or "Data ref: NCBI Sequence Read Archive PRJNA342805, 2017". In the Reference list, data citations are very similar to normal literature references but must be labeled with "[DATASET]" at the end of the reference. For detailed instructions please refer to our Author Guidelines .

- When you resubmit your manuscript, please download our CHECKLIST (https://bit.ly/EMBOPressAuthorChecklist) and include the completed form in your submission.
Please note that the Author Checklist will be published alongside the paper as part of the transparent process (https://www.embopress.org/page/journal/17444292/authorguide#transparentprocess).

If you feel you can satisfactorily deal with these points and those listed by the referees, you may wish to submit a revised version of your manuscript. Please attach a covering letter giving details of the way in which you have handled each of the points raised by the referees. A revised manuscript will be once again subject to review and you probably understand that we can give you no guarantee at this stage that the eventual outcome will be favorable.

Best wishes and Happy New Year,

Maria

Maria Polychronidou, PhD
Senior Editor
Molecular Systems Biology

We realize that it is difficult to revise to a specific deadline. In the interest of protecting the conceptual advance provided by the work, we recommend a revision within 3 months (11th Apr 2023). Please discuss the revision progress ahead of this time with the editor if you require more time to complete the revisions. Use the link below to submit your revision:

https://msb.msubmit.net/cgi-bin/main.plex

IMPORTANT: When you send your revision, we will require the following items:
1. the manuscript text in LaTeX, RTF or MS Word format
2. a letter with a detailed description of the changes made in response to the referees. Please specify clearly the exact places in the text (pages and paragraphs) where each change has been made in response to each specific comment given
3. three to four 'bullet points' highlighting the main findings of your study
4. a short 'blurb' text summarizing in two sentences the study (max. 250 characters)
5. a 'thumbnail image' (550px width and max 400px height, Illustrator, PowerPoint or jpeg format), which can be used as 'visual title' for the synopsis section of your paper.
6. Please include an author contributions statement after the Acknowledgements section (see https://www.embopress.org/page/journal/17444292/authorguide)
7. Please complete the CHECKLIST available at (https://bit.ly/EMBOPressAuthorChecklist).
Please note that the Author Checklist will be published alongside the paper as part of the transparent process (https://www.embopress.org/page/journal/17444292/authorguide#transparentprocess).

8. When assembling figures, please refer to our figure preparation guideline in order to ensure proper formatting and readability in print as well as on screen:
https://bit.ly/EMBOPressFigurePreparationGuideline
See also figure legend guidelines: https://www.embopress.org/page/journal/17444292/authorguide#figureformat
9. Please note that corresponding authors are required to supply an ORCID ID for their name upon submission of a revised manuscript (EMBO Press signed a joint statement to encourage ORCID adoption).
(https://www.embopress.org/page/journal/17444292/authorguide#editorialprocess)
Currently, our records indicate that the ORCID for your account is 0000-0002-8294-2995.

Please click the link below to modify this ORCID:
Link Not Available

10. At EMBO Press we ask authors to provide source data for the main manuscript figures. Our source data coordinator will contact you to discuss which figure panels we would need source data for and will also provide you with helpful tips on how to upload and organize the files.

The system will prompt you to fill in your funding and payment information. This will allow Wiley to send you a quote for the article processing charge (APC) in case of acceptance. This quote takes into account any reduction or fee waivers that you may be eligible for. Authors do not need to pay any fees before their manuscript is accepted and transferred to the publisher.

EMBO Press participates in many Publish and Read agreements that allow authors to publish Open Access with reduced/no publication charges. Check your eligibility: https://authorservices.wiley.com/author-resources/Journal-Authors/open-access/affiliation-policies-payments/index.html

As a matter of course, please make sure that you have correctly followed the instructions for authors as given on the submission website.

*** PLEASE NOTE *** As part of the EMBO Press transparent editorial process initiative (see our Editorial at https://dx.doi.org/10.1038/msb.2010.72), Molecular Systems Biology publishes online a Review Process File with each accepted manuscripts. This file will be published in conjunction with your paper and will include the anonymous referee reports, your point-by-point response and all pertinent correspondence relating to the manuscript. If you do NOT want this File to be published, please inform the editorial office at msb@embo.org within 14 days upon receipt of the present letter.

Reviewer #1:

Endothelial dysfunction is an important contributor to most cardiovascular diseases and needs to be better understood at the molecular level. In this manuscript, Pinheiro-de-Sousa et al. use multiple omics datasets to identify and validate new targets that modulate endothelial cell phenotype and may be useful in treating endothelial dysfunction. This study characterizes the response of human aorta endothelial cells exposed to a variety of inflammatory, metabolic, and mechanical stresses from publicly available ATAC-seq, CHiP-seq, and RNA-seq data. The data are integrated by network analysis and used to identify targets that are validated in new drug screening experiments for reactive oxygen species and inflammatory markers.

To validate their predictions, the authors performed a significant high-content screen of 17 drugs for 3 readouts under multiple contexts. The authors identified antioxidant and antiinflammatory roles of an EGLN3 inhibitor, which were further substantiated by measurements of mitochondrial ROS and E-selectin expression. They also measured downstream HIF1a expression and targets (Figure S4). Overall the combination of computational and experimental methods is a strength, with the strongest aspects being the experiments. The computational analysis and interpretation need to be more rigorous and clear.

Major comments
In Figure 2, the authors are searching for common signatures among multiple CV risk factors. As they did for ATACseq and CHiPseq in the supplement, they should quantify the degree of overlap in DEGs (looks low) and perhaps statistical significance. An alternative to complete overlap would be partial overlap with 3 of 5 datasets etc.

The most original part of the in silico analysis is the KO screen, which measures change in network topology. However the authors do not demonstrate how abstract topology measures such as RWR score and Jensen Shannon score relate to experimentally measurable functional outcomes from a biological network.

In Figure 3E, it is not clear why the authors compared the top vs bottom half of the KO genes? All of these KO genes were presumably predicted from the previous analysis to be in the ED network, so one should expect functional relevance for all of them. What are the p values for these comparisons? However the specific CVD related pathways are quite interesting.

The biological inferences from Figure 3F could not be interpreted. About half of the genes are targeted by drugs, and the Disgenet results were not interpreted quantitatively. This is a key transition to motivate the experiments.

Minor comments
The multiple references to Figure 2A are confusing. But it appears that Figure 2A shows just the common regions. Figure 2 D-F legend could also better clarify this is showing analysis of common regions/genes.

"Because we were interested in the common genes and pathways to these risk factors, we used the genes annotated to the 6,630 peaks that were differentially expressed in at least one condition". It is not clear why "one condition" would be used for commonality.

Why do the 100 common genes in Figure 2F not match the 86 genes from at least one condition in Figure 3? Why are there 86 seed genes but 81 KO genes? Overall there is a fair amount of confusion about which genes or peaks are being used it what part of the analysis, and how that gets passed to the next stage.

The position of the scRNAseq analysis in Figure 3 is confusing. It seems to not depend on Figure 3A.

The text around Figure 3 is out of order, making it difficult to follow.

Figure S2E and D were overlapping, so I could not see E.

In Figure S3, how were the drugs ordered? There seems to be a clear trend in panel C.

A number of references in the methods are listed as PMIDs.

Reviewer #2:

Summary
The scope of this study was to introduce an in silico pipeline to identify (and validate) novel targets for endothelial dysfunction using a wide selection of public data sets, databases and bioinformatic tools.
In detail, the authors use a range of stresses/treatments that are seen as surrogates for the risk factor of cardiovascular disease and define the common regulated set by selecting genes that are differentially expressed under any of those stresses, where the regulatory region was commonly found open (ATAC) and active (H3K27ac) and which have been demonstrated to be regulated by transcription factors whose motifs are enriched in the abovementionen ATAC+H3K27ac regions.
The found genes were used as seeds to probe the human protein interaction network (PIN) utilizing the Google pageRank algorithm against random background networks with preserved degrees to define the subnetwork relevant for the ED disease.
On the ED network the authors then simulated the knock out of each of the core genes and used the change in distance and topological parameters to assess the importance of each of the core genes. Based on those measures, the availability of drugs and association with CVDs they further selected 17 targets that were tested on human aortic endothelial cells for toxicity and their effect on ROS and inflammation (ICAM), finding one candidate drug that reduced ROS and ICAM and seven that reduced either process.

General remarks
The idea to combine the available data to search for new candidates is novel (particularly the pageRank approach) and important for the field and in parts very elegantly solved, in others not so.
Crucially the identification of the candidate genes and some filtering steps are not comprehensible. Also the relevance of the only followed up target seems shaky.
If properly presented this work could certainly be of interest to other researchers that study a complex disease and lack candidates but have sufficient high-throughput data at hand (e.g. cancer).

Major points

1. On several occasions the authors state that even based on their own research "combining diverse CVD risk factors results in complex gene network regulation that is not trivial to predict based on the summation of individual effects", but yet they concentrate here on genes that are commonly regulated by individual surrogates of CV risk factors. Wouldn't the quoted part imply that by concentrating on the intersection one would miss out targets relevant for the more complex case of interest? Can this information be even found in the used data sets?

2. The rationale to filter the common open and active promoter regions with differentially expressed genes of at least one condition I can follow but the next step of filtering those genes for genes known to interact with TF-motifs found in the centered regions seems to me an unneccessary bias as here instead of filtering out false positives one also filters out true positives that have simply been understudied.

TRRUST, the TF-target database used here is based on scanning medline abstracts for TF-gene interactions, which is highly influenced by research bias, i.e. which TF and target was in research focus and which ones not.

Furthermore why did the authors not restrict also the DEGs to be present in at least 2 conditions (or based on their PCA are present in both groups separated by PC1 or by PC2) to preserve the commonality (and reduce their core set to go straight to the PIN without TF-target filtering)?

3. It is unclear how the 17 genes that were targeted with drugs were selected. From the network analysis in Figure 3F only 9 of the 17 that are singled out as text (the choice of those is not strictly evident either as there are points around EGLN3 that are not named) are included. Also in the methods this part is incomplete and thresholds are not given. This is very crucial to fix!

4. The authors identified molidustat (introduced as HIF-1a stabilising drug) as a potential clinically relevant drug but their arguments and data around the involvement of EGLN3 is not convincing. As follow up instead of an functional assay such as a knockdown of EGLN3 only HIF-1a targets are investigated, which itself is the described target of the drug and can stabilization can be also achieved by the action on other PHDs. The authors claim in the discussion : "Here, we demonstrated the accumulation of HIF-1α protein when increasing the dose of Molidustat (Figure 4F). However, the protective effect of this drug was present at lower doses (10-8M and 10-6M)."
1) there is a noticeable induction of HIF-1a for 10-6M and there are only slight and partly insignificant changes of the ROS and ICAM for modulistat in the absence of IL1ß+OxPAC (Fig S3).
2) It is likely that a different context (i.e. IL1ß+OxPAC) will change the HIF-1a status, and thus whether through EGLN3 or another PHD will be more responsive to lower doses of molidustat.
3) in the methods section the authors write that in addition to IL1ß+OxPAC for the last hour they added H2O2 and PMA to stimulate ROS production, and by this will most likely induce HIF-1a too, hence the stronger effect even for lower concentrations of the drug.
So to maintain their claim of the role of EGLN3 they need to provide further data to substantiate this.

5. Seven more targets were found to be affecting a single process (ROS, inflammation). It would increase the value of this study if they also tested the reasonable combinations (12) to target both processes.

Minor points:

1. The verification of the ED network on artherosclerotic vasculature plaque data lacks specificity. What hypergeometric p-value would non-related diseases (e.g. cancer) that origin in the same cell type exhibit? And what subgroup was used for differential analysis? Only the VWF-high cells in Figure 3C, if yes what cutoff?
By what method were the three data sets integrated (no methods part for the scRNAseq analysis)?

2. Which number of selected DEGs for the network analysis is correct (and what is their identity)?
82 as stated in table S1 (DEGs, TF_DEGs) and the main text of fig 2
86 as stated in Fig 3A, and main text for fig 3
81 that were used for the KO simulations

3. Fig S2 is corrupted, subfigure C and E are overplotted by D

4. methods: paragraph "KO network and target gene prioritisation" is incomplete. There is an ennumeration starting with (ii) whith the beginning of that sentence missing.

5. Fig 3A in caption F comes before E (should be the other way round)

6. How were the genes selected for PCA Fig2C and hierarchical clustering in Fig S1D, I could not find anything in the methods section

7. Fig. 3D which one is in vitro and which one in vivo? Do you need identity as x axis labels?

8. Fig 4B/C (and Fig S3 B/C/D) it should be made clear in the figure what was tested, by e.g. putting the reference measurement first (or indicating the reference in the figure)
It would be also helpful to sort the drugs by their increasing or decreasing effect. It can also only be guessed that the points indicate mean values for each of the 96 wells, if that is true, please write this in the caption properly. Also the numbers for the drugs do not add any value, consider ommiting them for better readibility.
9. Fig 4 F why show standard deviation for 2 points and those two points are shown as well, then the sd-bars are unnecessary

10. Fig 4 caption it is written "Data are presented as mean +- standard deviation" (before point F) but until point F only boxplots are shown...

11 Common Typo: Wilcon test -> Wilcoxon test

Reviewer #3:

In this study, Pinheiro-de-Sousa et al used computational approaches to address endothelial dysfunction (ED) as a hallmark of CVD. Their aim was to bridge the gap in knowledge between the molecular basis of ED and finding direct therapeutic targets for ED, which combined would have the potential to ameliorate CVD.

The study is highly interesting, approach is innovative and potentially of high interest to the field, considering the key importance of ED for CVD and the exponential growth of available bioinformatic data for exploration. The study is also written and presented in an expert way, and methodologically seems generally sound. The main strength of this study is in novel computational approaches taken, which in a powerful way illustrate the potential of in silico knockouts to discover disease-specific candidate targets for drug development or repositioning. The experimental work has some weaknesses and limitations that put the computational part at a test.

Here are some major conceptual issues that should be addressed or reconciled, as specified below:

- Differential expression analysis considered the comparisons of OSS vs LSS, whereas IL-1β, TNF-α and OxPAPC treatments were compared to their static control owing to data availability. For further analyses, the genes common to the respective cardiovascular risk factor models were taken further. The simplification around taking the common genes takes some more detailed explanation, because this conceptually leads to the assumption that pathways of ED are always the same, regardless of the trigger. Or how do the authors justify this? Of course, it is more convenient for the sake of scaling down the computational work, but it does not fit with the biology which likely entails different capacity of endothelial cell response based on different predominant stimuli. And one should also not assume that vascular disease biologically combines all these stimuli at the same time-space scale.

- The work on re-analysis of ECs from human plaque and healthy artery scRNAseq data is also interesting, but conceptually very simplified. The assumption is that the origin of ECs in plaques and normal vessels is the same, which is not the case. ECs in plaques come from different 'compartments' (i.e. lumen vs neovessels) and even plaques from different patients are different, so how was this variability in EC origin and location accounted for?

- The authors don't actually show that Molidustat or other inhibitors used in this study really inhibit the gene of interest, in this case EGLN3/PDH3. It is likely that these inhibitors have other effects and particularly Molidustat possibly inhibits also PDH1 and PDH2. Gene and/or protein expression of target genes should also be evaluated for more specific understanding and interpretation of the inhibition effects.

- The above comment is particularly important in the light of abundant mouse knockout research around PDH isoforms in atherosclerosis, which has shown that PDH1 and 2 are involved in athero, but not PDH3 (Demandt JAF et al 2021; van Kuijk et al 2021). These studies have shown that whole-body PHD3 KO instigated an unfavorable lipid profile and increased hematocrit, in contrast to other PHD isoforms, yet without altering atherosclerotic plaque development. Myeloid-specific PHD3 knockout on high-fat diet displayed enhanced atherosclerotic plaque growth and macrophage apoptosis via HIF1A pathway, but no changes reported in ECs. These studies deserve to be discussed in the context of data presented here and certainly need to be reconciled.

Ref: MSB-2022-11462

Integrated Systems Biology Approach Identifies Gene Targets for Endothelial Dysfunction

We thank the reviewers for their comments and diligent suggestions. While performing the revisions, we started to have doubts about the ranking provided by the *in silico* perturbation analysis in our paper. We therefore decided to perform a full siRNA screen of all 81 genes that we identified as relevant for endothelial dysfunction.
Unfortunately, the *in silico* perturbation ranking was not correlated with the results from the siRNA screen. While this doesn't necessarily mean that it was wrong, it also doesn't provide us the confidence we need to publish it as a method in its present form. We therefore decided to remove it from the paper.

Nonetheless, the full siRNA screen of 81 ED targets provided novel and interesting insights. Firstly we discovered 31 potential targets for ED, in contrast to our previous version of the paper that was only able to provide support for 8. Secondly we found two groups of ED genes. The one group of genes are not in fact targets for endothelial dysfunction, but are rather essential for cell survival and reducing oxidative stress and inflammation in the disease condition. This is particularly interesting because several of these genes are in fact currently targeted for cardiovascular disorders, and they might be exacerbating endothelial dysfunction in these patients. The other group of genes indeed ameliorates ED phenotypes and could represent appropriate targets. Indeed, some of these are targets for cardiovascular disease but many of them are used in cancer therapy, suggesting potential for drug repurposing.

These two groups of genes were used as input to network analysis revealing network signatures and emergent properties of ED network rewiring for survival of the cells in the disease condition and of deregulation of endothelial cells that can be targeted.

As a result of our new findings, the paper has been substantially revised and large parts of it re-written. Many of the comments below, are therefore no longer relevant.

We nonetheless reply to each concern below in a point-by-point way, keeping in mind the new version of our manuscript.

Reviewer #1:

Endothelial dysfunction is an important contributor to most cardiovascular diseases and needs to be better understood at the molecular level. In this manuscript, Pinheiro-de-Sousa et al. use multiple omics datasets to identify and validate new targets that modulate endothelial cell phenotype and may be useful in treating endothelial dysfunction. This study characterizes the response of human aorta endothelial cells exposed to a variety of inflammatory, metabolic, and mechanical stresses from publicly available ATAC-seq, CHiP-seq, and RNA-seq data. The data are integrated by network analysis and used to identify targets that are validated in new drug screening experiments for reactive oxygen species and inflammatory markers.

To validate their predictions, the authors performed a significant high-content screen of 17 drugs for 3 readouts under multiple contexts. The authors identified antioxidant and antiinflammatory roles of an EGLN3 inhibitor, which were further substantiated by measurements of mitochondrial ROS and E-selectin expression. They also measured downstream HIF1a expression and targets (Figure S4). Overall the combination of

computational and experimental methods is a strength, with the strongest aspects being the experiments. The computational analysis and interpretation need to be more rigorous and clear.

Major comments

1.1 *In Figure 2, the authors are searching for common signatures among multiple CV risk factors. As they did for ATACseq and CHiPseq in the supplement, they should quantify the degree of overlap in DEGs (looks low) and perhaps statistical significance. An alternative to complete overlap would be partial overlap with 3 of 5 datasets etc.*

R1.1. We thank the reviewer for their comment and acknowledge that we did not quantify the degree of overlap in DEGs among the various CV risk factors, but we have now added this information (see Fig EV1E - copied below for convenience). As mentioned, we could have used statistical measures to assess the significance of overlap. However, our approach was based on prioritising the DEGs regulated by the TFs enriched in the common peaks from the ChIP and ATAC experiments, rather than the degree of overlap itself. The union of DEGs regulated by the respective TFs activity was used to build our ED disease network. It is worth noting that in a previous study (Pinheiro-de-Sousa et al., 2022), we performed a similar experiment using different stimuli individually and in combination, including OSS, IL1B, OxPAPC, and hypoxia. We showed that the sum of individual stimuli recapitulates only partially the conditions when they are all combined, indicating the appearance of emergent properties associated with the combined stimulation of the cells. In this current study, we based the analysis on the epigenetic layer, where environmental stimuli are integrated and used the common epigenetic peaks and TFs activity to prioritise the differentially expressed genes and build the networks. We have added a paragraph in the introduction to better link the present study to our previous work and explain using the epigenetic layer as the basis here. We have also clarified and better explained the approach in the first section of the results and discussed its limitations in the Discussion section.

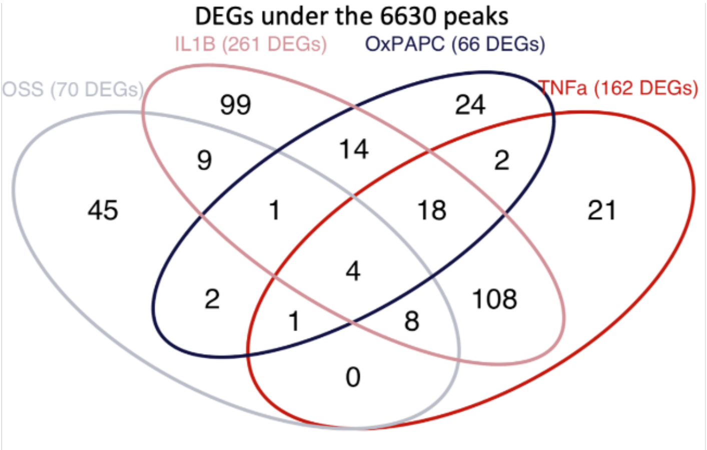

1.2. *The most original part of the in silico analysis is the KO screen, which measures change in network topology. However the authors do not demonstrate how abstract topology measures such as RWR score and Jensen Shannon score relate to experimentally measurable functional outcomes from a biological network.*

R1.2 We apologise for the lack of clarity. The *in silico* perturbation experiment has now been removed from the manuscript but to provide an answer to the comment: The

hypothesis on which we based the *in silico* perturbation experiment was that nodes that are central to the generation of the endothelial dysfunction network, are also more likely to be important for the phenotype. Based on our approach, the outcome of this *in silico* experiment depends on the topology and properties of the network and it is difficult to link directly to their effect on the phenotype, as mentioned in our discussion. Our ranking of the genes according to this prioritisation/disruption score enriched the top hits in relevant functions (previous Figure 3E; pasted below as the figure is no longer available in the new manuscript), even with the list itself as a background, we were therefore excited about the approach. However, partially prompted by your comment, we decided to validate our ranking experimentally, by performing an siRNA screen of all 81 ED genes and see whether the results correlate with our ranking. Unfortunately, we were not able to recapitulate experimentally the ranking of the *in silico* perturbation. We have therefore decided that it is not robust enough to include in this publication and have removed it. In the present version, we have added alternative network analysis that reveals two networks for endothelial dysfunction: one that identifies a signature that is essential for the cells to survive in the disease conditions and reduce the oxidative stress and inflammation ("adaptive rewiring"), and one that represents the deregulated part of the network that can be used for targeting. We describe this analysis in detail in our revised manuscript and our new Figures 4 and EV5.

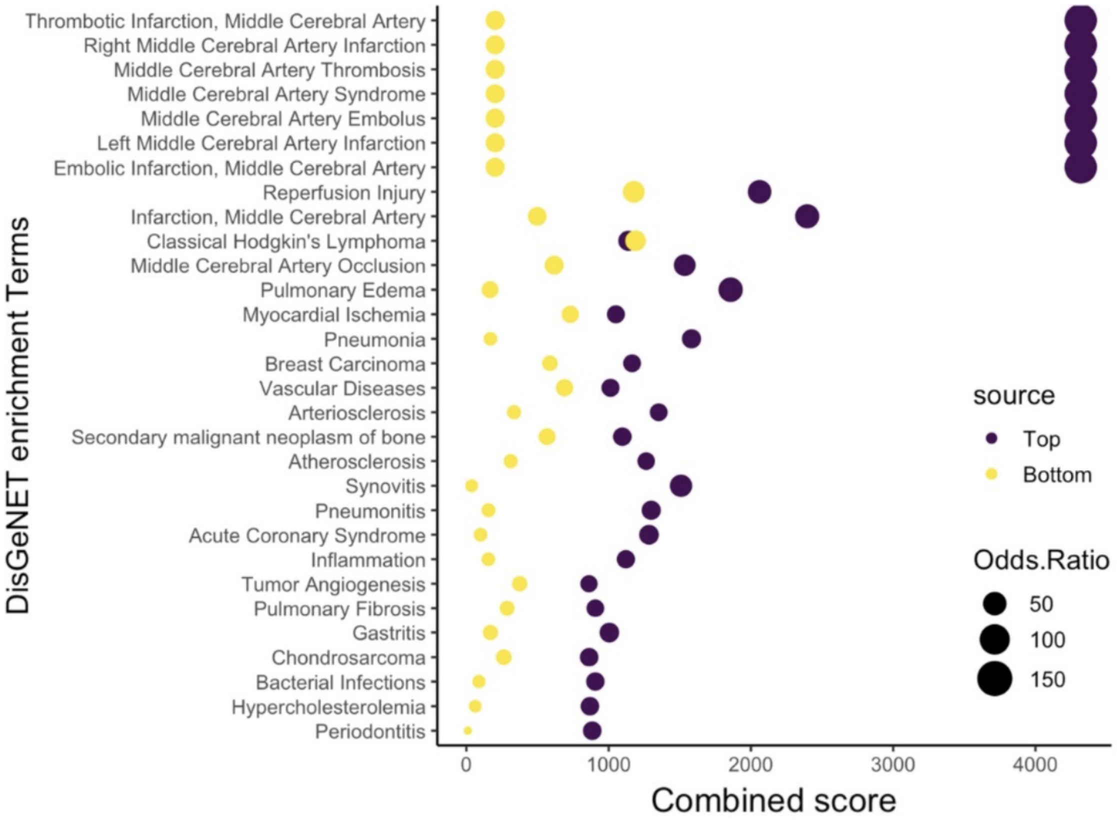

***1.3** In Figure 3E, it is not clear why the authors compared the top vs bottom half of the KO genes? All of these KO genes were presumably predicted from the previous analysis to be in the ED network, so one should expect functional relevance for all of them. What are the p values for these comparisons? However the specific CVD related pathways are quite interesting.*

R1.3 We apologise for the lack of clarity. All 81 genes used in the *in silico* KO experiment were predicted to be associated with ED, as they had been found as modulated in our integrated omics analysis and are the basis for the global ED network. Nonetheless, some of these genes were expected to be more strongly associated with ED, or to be more important for the generation and topology of the ED network. The *in silico* KO approach allowed us to rank them and the enrichment analysis of the top vs bottom half of the ranking is used to demonstrate that the ranking indeed prioritised the most relevant genes, even among the already relevant list. Essentially it was a sanity check that our *in silico* prioritisation of targets is actually working and to provide confidence to the top ranked candidates that we then selected for experimental validation. Since we were not able to recapitulate the ranking based on our new siRNA screen we have now removed this entire section.

1.4 *The biological inferences from Figure 3F could not be interpreted. About half of the genes are targeted by drugs, and the Disgenet results were not interpreted quantitatively. This is a key transition to motivate the experiments.*

R1.4. We have removed this section as our *in silico* perturbation-based ranking was replaced by the genetic perturbation in all genes followed by the network development

Minor comments

1.5 *The multiple references to Figure 2A are confusing. But it appears that Figure 2A shows just the common regions. Figure 2 D-F legend could also better clarify this is showing analysis of common regions/genes.*

R1.5 We have reduced the references to Figure 2A (currently Figure 1) to only those referring to the common regions between the ATAC and ChIP-seq data and have updated the Figure legend for clarity.

1.6 *"Because we were interested in the common genes and pathways to these risk factors, we used the genes annotated to the 6,630 peaks that were differentially expressed in at least one condition". It is not clear why "one condition" would be used for commonality.*

R1.6 We apologise for the confusion. What we meant here is that we only considered the DEGs that were also annotated in the peaks commonly identified by the ATAC and ChIP-Seq experiments. We rewrote this sentence to clarify this point.

1.7 *Why do the 100 common genes in Figure 2F not match the 86 genes from at least one condition in Figure 3? Why are there 86 seed genes but 81 KO genes? Overall there is a fair amount of confusion about which genes or peaks are being used it what part of the analysis, and how that gets passed to the next stage.*

R1.7 We thank the reviewer for the comment and apologise for the lack of clarity. The 100 common genes are the sum of the TFs enriched in the common 6,630 peaks plus the union of DEGs under those peaks, filtered by the TFs that have been linked before with those genes (using TTRUST database). However, to generate the ED network, we wanted to use significantly modulated genes, as this would help filter spurious hits and also practically the value of the log2FC of each gene is needed for the network

propagation. (Methods - Empirical network propagation). Among the 100 genes that were ED-relevant 86 were DEGs. The remaining TFs that were enriched in the peaks were not DEG in the conditions investigated and therefore were not used for the networks generation. Of the remaining 86 genes, 5 were downregulated in the disease conditions. As we wanted to silence genes that were overexpressed in the pathological conditions, we only performed the rest of the experiments and analysis with the remaining 81 upregulated genes. We have now clarified these points at the end of the first results section and discuss the limitation of this approach in the discussion.

1.8 *The position of the scRNAseq analysis in Figure 3 is confusing. It seems to not depend on Figure 3A.*

R1.8 The single-cell analysis served to provide orthogonal support for the ED network. The reviewer is correct that it is not directly relevant to the *in silico* perturbation analysis that was represented in Figure 3. We have therefore created a separate figure with Figure 3-D and moved it to the supplementary materials for improved clarity (Figure EV5). The *in silico* perturbation section has also been entirely replaced by our new network analysis (Figures 4 and EV6).

1.9 *The text around Figure 3 is out of order, making it difficult to follow.*

R1.9 The comment is no longer relevant since the figures have been revised.

1.10 *Figure S2E and D were overlapping, so I could not see E.*

R1.10 We apologise for this, all figures have now been revised.

1.11 *In Figure S3, how were the drugs ordered? There seems to be a clear trend in panel C.*

R1.11 They were ordered according to the buying list we received from the companies, no special order was taken into consideration. However, the experiment was performed randomly using different plates and on different days. We now only display the drugs that had a significant effect in the main figure and the full data can be found in Dataset EV2 ordered according to their combined ranking (Fig 3B). The raw imaging data from the plates is also available in Biostudies.

1.12 *A number of references in the methods are listed as PMIDs.*

R1.12 We have now updated these.

Reviewer #2:

The scope of this study was to introduce an in silico pipeline to identify (and validate) novel targets for endothelial dysfunction using a wide selection of public data sets, databases and bioinformatic tools.
In detail, the authors use a range of stresses/treatments that are seen as surrogates for the risk factor of cardiovascular disease and define the common regulated set by selecting genes that are differentially expressed under any of those stresses, where the regulatory region was commonly found open (ATAC) and active (H3K27ac) and which

have been demonstrated to be regulated by transcription factors whose motifs are enriched in the abovementionen ATAC+H3K27ac regions.
The found genes were used as seeds to probe the human protein interaction network (PIN) utilizing the Google pageRank algorithm against random background networks with preserved degrees to define the subnetwork relevant for the ED disease. On the ED network the authors then simulated the knock out of each of the core genes and used the change in distance and topological parameters to assess the importance of each of the core genes. Based on those measures, the availability of drugs and association with CVDs they further selected 17 targets that were tested on human aortic endothelial cells for toxicity and their effect on ROS and inflammation (ICAM), finding one candidate drug that reduced ROS and ICAM and seven that reduced either process.

General remarks
The idea to combine the available data to search for new candidates is novel (particularly the pageRank approach) and important for the field and in parts very elegantly solved, in others not so.
Crucially the identification of the candidate genes and some filtering steps are not comprehensible. Also the relevance of the only followed up target seems shaky.
If properly presented this work could certainly be of interest to other researchers that study a complex disease and lack candidates but have sufficient high-throughput data at hand (e.g. cancer).

Major points

***2.1** On several occasions the authors state that even based on their own research "combining diverse CVD risk factors results in complex gene network regulation that is not trivial to predict based on the summation of individual effects", but yet they concentrate here on genes that are commonly regulated by individual surrogates of CV risk factors. Wouldn't the quoted part imply that by concentrating on the intersection one would miss out targets relevant for the more complex case of interest? Can this information be even found in the used data sets?*

R2.1 We thank the reviewer for raising this issue. As the reviewer mentions, we had previously shown (Pinheiro-de-Sousa et al., 2022) that there are multiple relevant genes and processes and the addition of multiple stimuli also results in further emergent events that can not be predicted by the mere concatenation of the findings from each stimulus. However, in the same study, we had shown that there is approximately 56% overlap of genes identified by the different stimuli/surrogates of CVD risk factors, showing that there are also commonalities. We therefore hypothesised that, since all these diverse stimuli result in the same phenotype i.e. ED, the changes that they cause in the epigenome, i.e. the cell state, that are associated with ED should at least partially overlap. This hypothesis is key, as it allows us to improve the signal-to-noise ratio from the omics datasets used by focusing on the common chromatin regions affected by all the diverse conditions. The resulting network that is based on such an analysis is certainly not comprehensive. However it is significantly enriched in relevant genes for ED as shown from our functional analyses, and independent validation with the single cell data supporting our hypothesis. This way our approach provides a solid starting point to look for targets for ED. We have added these points, including the limitations of our approach to the discussion.

***2.2** The rationale to filter the common open and active promoter regions with differentially expressed genes of at least one condition I can follow but the next step of filtering those*

genes for genes known to interact with TF-motifs found in the centered regions seems to me an unnecessary bias as here instead of filtering out false positives one also filters out true positives that have simply been understudied. TRRUST, the TF-target database used here is based on scanning medline abstracts for TF-gene interactions, which is highly influenced by research bias, i.e. which TF and target was in research focus and which ones not. Furthermore why did the authors not restrict also the DEGs to be present in at least 2 conditions (or based on their PCA are present in both groups separated by PC1 or by PC2) to preserve the commonality (and reduce their core set to go straight to the PIN without TF-target filtering)?

R2.2 We thank the reviewer for these points. The premise of our study is that endothelial dysfunction is a cell state and as such should be reflected in the epigenetic layer through at least a subset of commonly affected chromatin regions by all conditions. By focusing on those, we aimed to improve our chances of identifying a core ED network, which we can use to prioritise targets for ED. These chromatin regions provide information regarding the genes that are potentially affected and the transcription factors that putatively regulate them. To further centre the network generation around these relevant TFs we decided to include only the DEGs known to be regulated by them. However, the reviewer is right that this way we are potentially underestimating the role of the understudied genes and TFs that are not annotated to specific TFs. Unfortunately, we can not now perform an siRNA screen for the 169 genes that are differentially expressed in at least two conditions, as we do not have the financial capacity to do so and also it takes several months to ship reagents to Brazil, that would further delay our already very delayed submission of our revised manuscript. Instead, we have highlighted in our new network analysis, the genes that are differentially expressed in at least two conditions, that were recovered by network propagation from the seed genes (the pro and anti-ED sets - please see revised manuscript; Figure 4A and B). We have also added a paragraph in the Discussion section to explain the reasoning behind focusing on the epigenetic layer and to explain the limitation of this approach.

2.3 *It is unclear how the 17 genes that were targeted with drugs were selected. From the network analysis in Figure 3F only 9 of the 17 that are singled out as text (the choice of those is not strictly evident either as there are points around EGLN3 that are not named) are included. Also in the methods this part is incomplete and thresholds are not given. This is very crucial to fix!*

R2.3 We apologise for the lack of clarity and details in the method description. At the time we had performed a ranking of the potential targets based on the aggregated score of Jensen Shannon Distance (JSD), Random Walk score (RWR) and Betweenness centrality (BW), which we had omitted to include in the Methods section by accident. However, as described in the methods the drugs were selected based on their commercial availability, $Kd/Ki/IC_{50}$ and assay quality. We have clarified this in the manuscript. As the *in silico* perturbation ranking did not agree with that from the siRNA screen we have removed the relevant figures and will not include the aggregated *in silico* perturbation score.

2.4 *The authors identified molidustat (introduced as HIF-1a stabilising drug) as a potential clinically relevant drug but their arguments and data around the involvement of EGLN3 is not convincing. As follow up instead of an functional assay such as a knockdown of EGLN3 only HIF-1a targets are investigated, which itself is the described target of the drug and can stabilization can be also achieved by the action on other PHDs.*

The authors claim in the discussion: "Here, we demonstrated the accumulation of HIF-1α protein when increasing the dose of Molidustat (Figure 4F). However, the protective effect of this drug was present at lower doses (10-8M and 10-6M)."

1) there is a noticeable induction of HIF-1a for 10-6M and there are only slight and partly insignificant changes of the ROS and ICAM for modulistat in the absence of IL1ß+OxPAC (Fig S3).

2) It is likely that a different context (i.e. IL1ß+OxPAC) will change the HIF-1a status, and thus whether through EGLN3 or another PHD will be more responsive to lower doses of molidustat.

3) in the methods section the authors write that in addition to IL1ß+OxPAC for the last hour they added H2O2 and PMA to stimulate ROS production, and by this will most likely induce HIF-1a too, hence the stronger effect even for lower concentrations of the drug. So to maintain their claim of the role of EGLN3 they need to provide further data to substantiate this.

R2.4 We apologise for the confusion. The reason for measuring HIF1a stabilisation and its targets was to confirm that indeed our drug is targeting EGLN3. The main increase of HIF-1a was at a concentration of $10x10^{-6}$M (old Fig S3 current Appendix Fig S1) whereas the imaging experiments where we observed the protective effect of molidustat showed a significant effect at both 10^{-8}M and 10^{-6}M, hence our statement above still holds.

However, the reviewer is correct that the drug targets the entire PHD family, and therefore we have now performed siRNA experiments targeting the different members of the family to demonstrate that the ROS protective effect is EGLN3-specific. The reviewer is correct that Molidustat is not exerting its effect through EGLN3, but surprisingly neither from EGLN1 or 2. We have mentioned this as a curiosity in the manuscript. Looking into Chemical Checker to identify potential similar drugs to identify other pathways through which Molidustat might function, we found NAD+ biosynthesis as enriched, however the link is too tentative to include as a fact in the manuscript. As it is beyond the scope of the paper for follow up on the mechanism of action of Molidustat we instead focus on other hits from the siRNA screen which are clearer.
Finally, regarding the third point of the reviewers, it was a result of a lack of clarity in the methods section: we did not add H_2O_2 or PMA to the wells treated with drugs but rather this was an additional control for ROS in a separate well. In the treated experiment we only had IL1ß+OxPAPC plus the drug. These experiments have now been removed from the main manuscript to the Appendix as we have shifted the focus from Molidustat to other targets.

2.5 *Seven more targets were found to be affecting a single process (ROS, inflammation). It would increase the value of this study if they also tested the reasonable combinations (12) to target both processes.*

R2.5 We thank the reviewer for the comment. We have now tested the combination of drugs that only worked in one of the readouts we measured. Specifically we tested the combinations: UGCG+RIPK2, UGCG+CXCR4, UGCG+DUSP1, UGCG+CXCL12; PTGS2+RIPK2, PTGS2+CXCR4, PTGS2+DUSP1, CCL2+CXCL12; CCL2+CXCR4, CCL2+DUSP1, CCL2+CXCL12; Unfortunately, the combination of the drugs did not provide an advantage in terms of improving both phenotypes tested. Instead, it seems

that the drugs cancelled the protective effect of the other one in the pair and one pair (PTGS2+RIPK2) even made the phenotype worse. We are therefore not going to include these results in the manuscript, but it will be interesting in future studies to explore why this is the case.

Minor points:

***2.6** The verification of the ED network on atherosclerotic vasculature plaque data lacks specificity. What hypergeometric p-value would non-related diseases (e.g. cancer) that origin in the same cell type exhibit? And what subgroup was used for differential analysis? Only the VWF-high cells in Figure 3C, if yes what cutoff? By what method were the three data sets integrated (no methods part for the scRNAseq analysis)?*

R2.6 We apologise for the omission. We have now added a "scRNAseq analysis" section in the Methods where we clarify all these points and provide all the details needed to reproduce the analysis.

***2.7** Which number of selected DEGs for the network analysis is correct (and what is their identity)? 82 as stated in table S1 (DEGs, TF_DEGs) and the main text of fig 2; 86 as stated in Fig 3A, and main text for fig 3; 81 that were used for the KO simulations*

R2.7 We apologise for the confusion. 86 was the total number of DEGs found to be associated with TFs overlapping with the relevant chromatin peaks. Of these 5 were downregulated, and we therefore proceeded with the 81 upregulated genes for the network analysis, as we wanted to identify genes that could be targeted for tackling ED. We have added a clarification in the end of the first section of the Results. Dataset EV1 contains the initially identified 100 DEGs and TF-GEDs.

***2.8** Fig S2 is corrupted, subfigure C and E are overplotted by D*

R2.8 We apologise for this, the figures have now been modified.

***2.9** methods: paragraph "KO network and target gene prioritisation" is incomplete. There is an ennumeration starting with (ii) whith the beginning of that sentence missing.*

R2.9 This section has now been deleted from the manuscript due to inability to validate the rankings of the in silico perturbation approach.

***2.10** Fig 3A in caption F comes before E (should be the other way round)*

R2.10 We have completely modified the figure.

***2.11** How were the genes selected for PCA Fig2C and hierarchical clustering in Fig S1D, I could not find anything in the methods section*

R2.11 We apologise for the omission. We have now added this information in the Methods section "**RNA-seq analysis and TF-DEGs associations"**

***2.12** Fig. 3D which one is in vitro and which one in vivo? Do you need identity as x axis labels?*

R2.12 We have removed the label 'Identity' from the x axis, and added in vivo/in vitro annotation. Please note that following a comment from a different reviewer, this figure has been separated to move the single cell analysis in the supplement (Appendix Fig S2)

2.13 *Fig 4B/C (and Fig S3 B/C/D) it should be made clear in the figure what was tested, by e.g. putting the reference measurement first (or indicating the reference in the figure)*

R2.13 We have amended the figures accordingly. We have put the reference measurement first in the Figure 2 A-E, Figure EV3 D and Figure EV4 C-F. Besides that, we have indicated how the comparisons among the conditions were made in the legend text. In the Figure EV4 B, we have indicated how the comparison was made in the legend.

2.14 *It would be also helpful to sort the drugs by their increasing or decreasing effect. It can also only be guessed that the points indicate mean values for each of the 96 wells, if that is true, please write this in the caption properly. Also the numbers for the drugs do not add any value, consider omitting them for better readability.*

R2.14 Thank you for the comment. Since we have now performed the systematic siRNA screen, we use the drug screen only as an independent way to provide further evidence for some of the targets identified in our siRNA screen. We therefore only show the ones that had a significant effect on the phenotype and the full dataset is moved to Dataset EV2. We have updated the legend to capture that the points indicate median values for each well.

2.15 *Fig 4 F why show standard deviation for 2 points and those two points are shown as well, then the sd-bars are unnecessary*

R2.15 That is a fair point, we have now increased the number of experiments to 4 (Appendix Figure S1A).

2.16 *Fig 4 caption it is written "Data are presented as mean +- standard deviation" (before point F) but until point F only boxplots are shown...*

R2.16 We have removed this from the figure legend.

2.17 *Common Typo: Wilcon test -> Wilcoxon test*

R2.17 We have updated all instances of Wilcon test to Wilcoxon test

Reviewer #3:

In this study, Pinheiro-de-Sousa et al used computational approaches to address endothelial dysfunction (ED) as a hallmark of CVD. Their aim was to bridge the gap in knowledge between the molecular basis of ED and finding direct therapeutic targets for ED, which combined would have the potential to ameliorate CVD.

The study is highly interesting, approach is innovative and potentially of high interest to the field, considering the key importance of ED for CVD and the exponential growth of available bioinformatic data for exploration. The study is also written and presented in an expert way, and methodologically seems generally sound. The main strength of this

study is in novel computational approaches taken, which in a powerful way illustrate the potential of in silico knockouts to discover disease-specific candidate targets for drug development or repositioning. The experimental work has some weaknesses and limitations that put the computational part at a test.

Here are some major conceptual issues that should be addressed or reconciled, as specified below:

***3.1** Differential expression analysis considered the comparisons of OSS vs LSS, whereas IL-1β, TNF-α and OxPAPC treatments were compared to their static control owing to data availability. For further analyses, the genes common to the respective cardiovascular risk factor models were taken further. The simplification around taking the common genes takes some more detailed explanation, because this conceptually leads to the assumption that pathways of ED are always the same, regardless of the trigger. Or how do the authors justify this? Of course, it is more convenient for the sake of scaling down the computational work, but it does not fit with the biology which likely entails different capacity of endothelial cell response based on different predominant stimuli. And one should also not assume that vascular disease biologically combines all these stimuli at the same time-space scale.*

R3.1 We thank the reviewer for raising this important comment. With this study we did not mean to suggest that all pathways relevant to ED are always the same. Indeed we had previously shown (Pinheiro-de-Sousa et al., 2022) that there are multiple relevant genes and processes and the addition of multiple stimuli also results in further emergent events that can not be predicted by the mere concatenation of the findings from each stimulus. However, in the same study we had shown that there is approximately 56% overlap of genes identified by the different stimuli/surrogates of CVD risk factors, showing that there are also commonalities. We therefore hypothesised that, since all these diverse stimuli result in the same phenotype i.e. ED, the changes that they cause in the epigenome that are associated with ED should at least partially overlap. This hypothesis is key, as it allows us to improve the signal-to-noise ratio from the omics datasets used by focusing on the common chromatin regions affected by all the diverse conditions. The resulting network that is based on such an analysis is certainly not comprehensive and doesn't assume that all stimuli are present at the same time, since it focuses on the common effects. However it is significantly enriched in relevant genes for ED as shown from our functional analyses, orthogonal validation with the single cell data and our siRNA experiments, supporting our hypothesis. This way our approach provides a solid starting point to look for targets for ED and indeed 83% of our prioritised genes had an effect on at least one ED phenotype when silenced. We have added these points to the discussion along with the limitations of this approach.

***3.2** The work on re-analysis of ECs from human plaque and healthy artery scRNAseq data is also interesting, but conceptually very simplified. The assumption is that the origin of ECs in plaques and normal vessels is the same, which is not the case. ECs in plaques come from different 'compartments' (i.e. lumen vs neovessels) and even plaques from different patients are different, so how was this variability in EC origin and location accounted for?*

R3.2 This is a great point. We know that atherosclerotic plaques can be very heterogeneous, so for the single-cell analysis we had to account for the location as batches. Currently, for building human cell atlases of different organs, and tissues, we

have to integrate different datasets generated from different donors, tissues, or single cells technologies, and several tools have been developed to account for all these batch effects (Malte D. Luechen et al., 2022). Here, we applied a method for integrating the datasets called canonical correlation analysis (CCA) (Hardonn D.R & Shawe-Taylor J, 2011), where we manage to cluster all the ECs independently of the vascular region because we removed the effect of ECs location. This approach allowed us to make the comparison of ECs present in the plaque vs healthy ECs, and we identified known genes present in ECs of atherosclerotic plaques such as MGP, VIM and B2M (Appendix Fig S3), which we considered as a sanity check of our analysis. We have updated the Methods to include more details for this analysis.

3.3 *The authors don't actually show that Molidustat or other inhibitors used in this study really inhibit the gene of interest, in this case EGLN3/PDH3. It is likely that these inhibitors have other effects and particularly Molidustat possibly inhibits also PDH1 and PDH2. Gene and/or protein expression of target genes should also be evaluated for more specific understanding and interpretation of the inhibition effects.*

R3.3 Thank you very much for this important comment. Based on the results of siRNA knockdown of EGLN1,2 and 3, it seems like the reviewer is correct that the effects are not a result of targeting EGLN3, but surprisingly they are also not a result of targeting EGLN1 or 2 (Appendix Fig S1 D and E, pasted below for convenience). We can not preclude that they are a result of targeting a combination of these proteins but it is not easy to deconvolve this with our current data. Since the *in silico* perturbation in the end was not validated by our full *in silico* perturbation screen (see revised manuscript) and in these results knockdown of EGLN3 did not seem to significantly affect neither the pro-ED nor the anti-ED networks, we decided that it is beyond the scope of the paper to try to decipher the mechanism of action of Molidustat in these phenotypes, though we still report it as an observation.

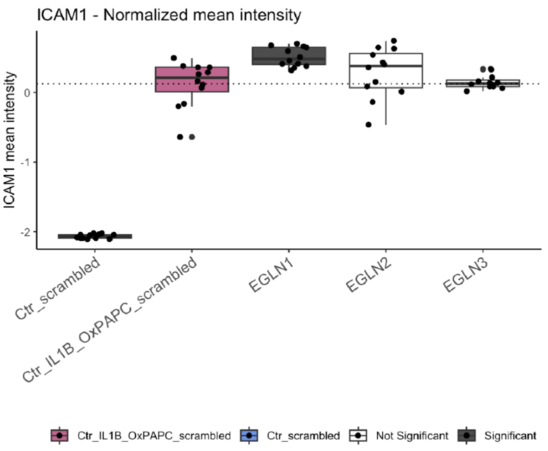

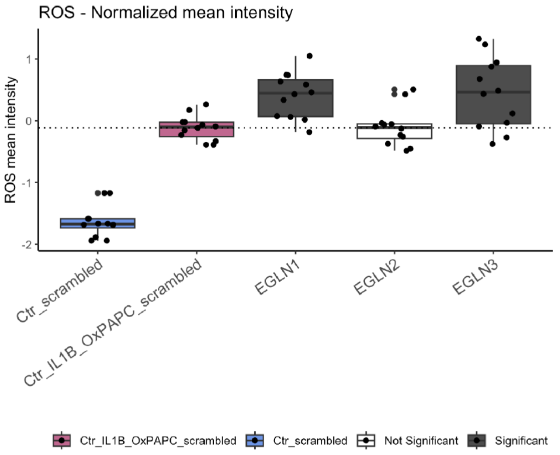

3.4 *The above comment is particularly important in the light of abundant mouse knockout research around PDH isoforms in atherosclerosis, which has shown that PDH1 and 2 are involved in athero, but not PDH3 (Demandt JAF et al 2021; van Kuijk et al 2021). These studies have shown that whole-body PHD3 KO instigated an unfavorable lipid profile and increased hematocrit, in contrast to other PHD isoforms, yet without altering atherosclerotic plaque development. Myeloid-specific PHD3 knockout on high-fat diet displayed enhanced atherosclerotic plaque growth and macrophage apoptosis via HIF1A*

pathway, but no changes reported in ECs. These studies deserve to be discussed in the context of data presented here and certainly need to be reconciled.

R3.4 We thank the reviewer for this information. Since EGLN3 is indeed not the reason for the observed effects of Molidustat (see R3.3) and since it is also not one of the newly identified relevant genes, we have removed extensive discussions related to EGLN3 from the manuscript and only mention the effect of molidustat as an observation. .

22nd Sep 2023

Manuscript Number: MSB-2022-11462R
Title: Integrated Systems Biology Approach Identifies Gene Targets for Endothelial Dysfunction

Dear Evangelia,

Thank you for sending us your revised manuscript. We have now heard back from the three reviewers who were asked to evaluate your revised study. As you will see below, the reviewers think that the study has improved as a result of the performed revisions. However, reviewer #1 and reviewer #3 mention that the removal of the in silico network analysis is somewhat disappointing and detracts from the methodological advance presented by the study. During our cross-commenting process, in which the reviewers get the chance to make additional comments in light of the other reports, we specificially consulted with them on the suitability of the study for publication in MSB given the reduced novelty of the computational workflow. On balance, the reviewers mentioned that while the reduced methodological novelty represents a limitation, their integration of multiple data sets to find causal regulators of endothelial cell dysfunction is of value for the field. As such, we have decided to invite you to submit a revised study, to address the reviewers' concerns indicated below.

We would also ask you to address some remaining editorial issues listed below.

- Our data editors have noticed some unclear or missing information in the figure legends, please see the attached .doc file. Please make all requested text changes using the attached file and *keeping the "track changes" mode* so that we can easily access the edits made.

- Please include page numbers in the Appendix Table of Contents.

-The Source data files need to be reorganized to one file (or ZIP folder) per main figure. Source Data for all EV and/or Appendix figures should be provided in a single ZIP folder.

- Our data integrity analyst noted a potential instances of figure panel reuse i.e. Figure EV2 A and C (ctr-Scrambled DAPI) and Figure EV2B and EV3C (scrambled IL1B&OxPAPC - DAPI and CM-H2DCFDA). We would ask you to indicate the image reuse in the respective figure legends for transparency.

- Table EV1-2 are rather long, we would ask you to provide them as Datasets EV5-EV6. Please make sure that the callouts in the text are corrected accordingly.

Please resubmit your revised manuscript online, with a covering letter listing amendments and responses to each point raised by the referees. Please resubmit the paper **within one month** and ideally as soon as possible. If we do not receive the revised manuscript within this time period, the file might be closed and any subsequent resubmission would be treated as a new manuscript. Please use the Manuscript Number (above) in all correspondence.

Click on the link below to submit your revised paper.

https://msb.msubmit.net/cgi-bin/main.plex

As a matter of course, please make sure that you have correctly followed the instructions for authors as given on the submission website.

Thank you for submitting this paper to Molecular Systems Biology.

Kind regards,

Maria

Maria Polychronidou, PhD
Senior Editor
Molecular Systems Biology

If you do choose to resubmit, please click on the link below to submit the revision online before 22nd Oct 2023.

https://msb.msubmit.net/cgi-bin/main.plex

IMPORTANT:
Please note that corresponding authors are required to supply an ORCID ID for their name upon submission of a revised manuscript (EMBO Press signed a joint statement to encourage ORCID adoption).
(https://www.embopress.org/page/journal/17444292/authorguide#editorialprocess)
Currently, our records indicate that the ORCID for your account is 0000-0002-8294-2995.

Please click the link below to modify this ORCID:
Link Not Available

The system will prompt you to fill in your funding and payment information. This will allow Wiley to send you a quote for the article processing charge (APC) in case of acceptance. This quote takes into account any reduction or fee waivers that you may be eligible for. Authors do not need to pay any fees before their manuscript is accepted and transferred to the publisher.

As a matter of course, please make sure that you have correctly followed the instructions for authors as given on the submission website.

*** PLEASE NOTE *** As part of the EMBO Press transparent editorial process initiative (see our Editorial at https://dx.doi.org/10.1038/msb.2010.72 , Molecular Systems Biology will publish online a Review Process File to accompany accepted manuscripts. When preparing your letter of response, please be aware that in the event of acceptance, your cover letter/point-by-point document will be included as part of this File, which will be available to the scientific community. More information about this initiative is available in our Instructions to Authors. If you have any questions about this initiative, please contact the editorial office (msb@embo.org).

Reviewer #1:

In this revision, the authors removed the in silico network analysis that was previously a primary focus of the innovation of this manuscript due to concerns that it did not make accurate predictions. In its place, they add an siRNA screen of 81 endothelial dysfunction (ED) associated genes and a different network analysis for Figure 4. The main significance and relevance to Molecular Systems Biology appears to have been lost with this revision. The results of network analysis are compared to selected genes from public scRNA-seq data but no longer validated by new experiments.

MSB Revision
1.2. The authors removed the most original part of the in silico analysis, the virtual knockout screen, due to technical concerns or its ability to accurately predict gene functions.

The authors replace the in silico analysis with an siRNA screen of 81 ED genes, finding it did not correlate well with the in silico analysis. This siRNA screen appears quite successful, finding phenotypes for quite a lot of the siRNAs. However these data are still in "screen mode" as they have not been validated for knockdown levels or secondary assays.

A new network analysis is added, in which the pro- or anti-ED genes were expanded to form protein-protein interaction networks. This network analysis does not have the novelty or anticipated significance of what was hoped from the previous network analysis.

1.3 no longer relevant as in silico analysis removed
1.4 no longer relevant as in silico analysis removed
It may help to quote modified text, as in several places it is not clear what changes were made in response to a particular comment.

In the new Figure 4, a network propagation is performed from the knockdown genes. Protein interaction network generated from 26 pro-ED and 31 anti-ED genes. It is not clear what this network predicts, or that it is sufficiently validated. The novelty of this network analysis is not demonstrated, comparable to how the in silico analysis was a central feature of the previous version of this manuscript. The network analysis is partially validated with literature scRNA-seq data using selected genes that are part of network. More direct comparisons are needed in the figures.

Reviewer #2:

The authors have addressed my previous comments exhaustively.
Overall the manuscript did improve for some parts and provides a useful resource for CVD research.

The story that unfolds in fron of me is the following:
1. combine epigenetic, gene expression, TF-database information to arrive at a candidate list for ED-related factors
2. knockdown of those factors and monitoring of ED-relevant readouts-> ranking into pro-and anti-ED seeds.
3. expansion of seeds to arrive at pro- and anti-ED network.
4. characterisation by databases and vascular plaque experiments.
5. test of available drugs that target ED-related factors, overlap with siRNA data indicates most promising candidate drugs.
Some comments for the new manuscript (which have surfaced to the provided extra information and the restructuring of the whole manuscript):

Major:

(i) As outlined above the flow of the paper would benefit from moving the drug testing to the end.
Additionally it would benefit from making this figure more informative: use drug names instead of the "fitting" target (except confidential drugs). Do all drugs only target the indicated genes or do they have multiple targets among the candidate genes (or in the Pro-and anti-ED PINs)? A summary figure combining insights of the siRNA screen and drug test would be of benefit.

(ii) Figure EV1 D:
Even though the materials and methods section states now the RNAseq data has been batch-corrected, there seems to be a remaining batch effect for OSS as well as LSS/FSS samples. Where does this stem from?
Crucially this could influence the differential gene expression results if not accounted for in the DESeq2 design formular (i.e. provided as an extra factor).

Minor:

(1) Figure EV1 D: Text always refers to LSS but the samples are indicated as FSS here, what was the correct treatment?

(2) Figure 2 and 3:
- General: Color scale of Z-score is not symmetric around 0 (i.e. 0 is not white). Please correct in figures A-E.
- In Ranking Score plots what do the two dots per gene in the figure represent, a confidence interval? I could not find an explanation for this anywhere.

(3) Regarding the ChEMBL and OPenTarget drugs selection:
I am missing a list that summarizes the used drugs along with target, predicted Kd/Ki or IC50 and other filtering criteria. Manually checking, I could not recapitulate that IL6 is a binding target for prednisolone.

(4) Page 7: VE cadherin staining genes states inconsistent numbers (21 in total, 20 decreasing and 3 increasing)

(5) Figure EV5 F:
What do the authors show in the three confusion tables if not the frequencies required for the hypergeometric test. What is the interpretation of positive and negative residuals with two digits behind the comma? What is the circle size indicating?

(6) Typos:
- page 25 CellProfiler (r missing)
- Legend Figure 2 and 3 "and nd nuclei" (perhaps you meant "nb" instead of nd?)
- Legend EV1: "motif sequence at and enrichment log p" (remove "at"?)
- Figure EV2 C: "ration" instead of ratio

Reviewer #3:

In this study, Pinheiro-de-Sousa et al aim to provide a foundation for constructing ED disease networks and identifying key node genes that have the potential to be therapeutic targets for cardiovascular diseases.
Their approach is still dominantly computational, but in the revised version of the manuscript they have incorporated more experimental data, both from gene silencing and pharmacological in vitro experiments. Owing to the removal of computational knockout analyses from the manuscript, which was an exciting and novel systems biology approach, this revised version is slightly less innovative in the technical sense, but not any less interesting and important. The identification and systematic silencing of 81 core ED genes, where 68 genes were shown to have a significant impact on at least one of the ED readouts, is a confident and very important result for the field that will likely constitute a basis for further functional and mechanistic studies of these genes by other groups. Moreover, none of the identified genes appeared to be essential in the absence of the pathological stimuli, suggesting their specific relevance to cell survival and function in the context of ED. Also, the Authors demonstrated that the commonly used practice of identifying deregulated genes in single omics datasets, may result in erroneous targeting of non-causal processes, potentially exacerbating the disease phenotype.
In addition to the fact that the manuscript is very well written, presented and coherent, as well as being technically sound, the

Authors have answered all my previous comments and requests and made suitable revisions. Taking everything together, I believe that their findings highlight the potential of integrating omics datasets from diverse conditions, to distinguish causal from adaptive processes and prioritise targets for therapeutic modulation of these phenotypes.

Ref: MSB-2022-11462R

Integrated Systems Biology Approach Identifies Gene Targets for Endothelial Dysfunction

We thank the reviewers for their thoughtful comments. Below we present a point-by-point response to their concerns.

Reviewer #1:

In this revision, the authors removed the in silico network analysis that was previously a primary focus of the innovation of this manuscript due to concerns that it did not make accurate predictions. In its place, they add an siRNA screen of 81 endothelial dysfunction (ED) associated genes and a different network analysis for Figure 4. The main significance and relevance to Molecular Systems Biology appears to have been lost with this revision. The results of network analysis are compared to selected genes from public scRNA-seq data but no longer validated by new experiments.

1.1. The authors removed the most original part of the in silico analysis, the virtual knockout screen, due to technical concerns or its ability to accurately predict gene functions.

R1.1 We thank the reviewer for the comment. It's important to clarify that our primary objective has consistently been the identification of new endothelial dysfunction (ED) candidate targets, with the *in silico* analysis serving as a means to that end. Our new data brings us closer to this goal, identifying more potential targets. In addition, we identify an adaptation network and a dysfunction network providing more insight into the mechanisms underpinning the disease. As such, we have provided more insight into the mechanisms of ED, and importantly demonstrated the need to consider that gene deregulation in omics data doesn't necessarily make it part of the disease phenotype, but rather could represent the cell's adaptation to maintain functionality and survive. Keeping in mind that there is an equal number of drugs targeting both the anti- and pro-ED networks, this an important finding to inform the design of therapies for ED and beyond.

1.2 The authors replace the in silico analysis with an siRNA screen of 81 ED genes, finding it did not correlate well with the in silico analysis. This siRNA screen appears quite successful, finding phenotypes for quite a lot of the siRNAs. However these data are still in "screen mode" as they have not been validated for knockdown levels or secondary assays.

R.1.2 Our current siRNA screen has several features that make the validation of knockdown levels unnecessary. Firstly, we used a combination of four siRNAs for each gene, to guarantee higher efficiency in silencing each target. Specifically, to avoid the need for secondary knockdown validation experiments, we used Dharmacon's SMARTpool ON-TARGET plus reagents and protocol, which guarantees the silencing of 75% of the target gene's expression [https://horizondiscovery.com/en/gene-modulation/knockdown/sirna/products/on-targetplus-sirna-reagents#guarantee; reference on pooled siRNAs performance Parsons et al, PLoS ONE, 2009].

Nonetheless, we validated silencing at the protein-level for select genes, such as ICAM1 and VE-Cadherin (Figure EV2 A & C), showing significant decreases in protein

expression. Additionally, we used siPLK1 to stimulate cell death, adding another layer of confirmation for siRNA functionality. The functional ED readouts that we used, and the general agreement of our results with the orthogonal drug screen that we had performed previously also provide evidence that the knockdowns were reasonably effective.

1.3 A new network analysis is added, in which the pro- or anti-ED genes were expanded to form protein-protein interaction networks. This network analysis does not have the novelty or anticipated significance of what was hoped from the previous network analysis.

R.1.3 We are sorry to read that the reviewer doesn't find our new network analysis innovative. The network analysis performed in our revised manuscript allows us to integrate the targets into functional context and only through this analysis were we able to make the observation that the adaptation network is more precise and involves distinct functional modules, whereas the deregulation network involves more pleiotropic and interconnected genes (Fig 3 & FigEV4B). This is a new insight into network rewiring in disease conditions, as it has not been previously described, to our knowledge.

Moreover, the network representation allows us to identify processes rather than individual genes that are involved in network adaptation and deregulation, and perform the drug analysis to highlight the fact that several drugs for cardiovascular disease could be aggravating ED as they target the adaptation network. Overall, the network analysis provided several interesting findings. While the network propagation method per se is not new, we show that our approach of combining the integration of multi-omics data, with genetic screens including functional readouts and network analysis allows enhanced interpretation of disease mechanisms and to distinguish causal from adaptive processes. (please see also R1.5 and comments in the text referring to response R1.5)

1.4 It may help to quote modified text, as in several places it is not clear what changes were made in response to a particular comment.

R1.4 We have now added comments to indicate where each concern in this revision is addressed.

1.5 In the new Figure 4, a network propagation is performed from the knockdown genes. Protein interaction network generated from 26 pro-ED and 31 anti-ED genes. It is not clear what this network predicts, or that it is sufficiently validated. The novelty of this network analysis is not demonstrated, comparable to how the in silico analysis was a central feature of the previous version of this manuscript. The network analysis is partially validated with literature scRNA-seq data using selected genes that are part of network.

R.1.5 In the current version of this manuscript, the role of the network analysis is quite different from the previous one. Instead of predicting the network topology changes when a gene is removed from the network (*in silico* KO), we used the network propagation to gain further insights into adaptive vs causal disease network rewiring. This is explained in the first sentence of the Network Propagation section "To gain better insights into the processes that are associated with cell survival during ED and the disease phenotype, we employed a network analysis strategy." This approach has unveiled interesting findings we would not have uncovered by examining these genes individually or through traditional enrichment analysis of the 26 pro-ED and 31 anti-ED genes. To demonstrate the added insight into processes that we can get through the network analysis, we have now added an enrichment analysis using only the seed genes compared to the full network in Fig EV4 D & E; Please see also answer R1.3 for more insights that we got from the network analysis.

Our analysis of *in vivo* single-cell data serves to evaluate the relevance of these networks in patients. Despite the complexity of genetic and environmental interactions in human samples, our networks displayed significant enrichment in transcriptomics within ECs from human atherosclerotic plaques, particularly in the case of the anti-ED network. This result is exciting for two main reasons: Firstly, this enrichment not only validates the relevance of our ED disease gene networks within the *in vivo* setting, especially within the context of atherosclerotic vasculature, but it also establishes a causative link. This evidence of causation in human conditions can serve as a bridge for translational applications by identifying players in both, *in vitro* and *in vivo* conditions. Secondly, it reaffirms the robustness of our comprehensive system biology approach, in which the network analysis was key to digest the integration of epigenomic, transcriptomic and siRNA screening results into biology clues. We have revised sections in both the results and discussion to highlight the important role of our network approach in revealing the insights we have presented, and have added comment tags in the discussion where the insights described are stemming from the network analysis.

1.6 More direct comparisons are needed in the figures.

R1.6. In our manuscript, we are comparing the pro- with the anti-ED network, and in this revision, we are adding a comparison with the seed nodes (Fig EV4 D & E). We have also added a figure that integrates the results of the drug screen with the siRNA screen (Figure 4D & Fig EV6G). It is unclear to us what other comparisons would be of interest.

Reviewer #2:

The authors have addressed my previous comments exhaustively. Overall the manuscript did improve for some parts and provides a useful resource for CVD research. The story that unfolds in from of me is the following:

1. combine epigenetic, gene expression, TF-database information to arrive at a candidate list for ED-related factors

2. knockdown of those factors and monitoring of ED-relevant readouts-> ranking into pro-and anti-ED seeds.

3. expansion of seeds to arrive at pro- and anti-ED network.

4. characterisation by databases and vascular plaque experiments.

5. test of available drugs that target ED-related factors, overlap with siRNA data indicates most promising candidate drugs.

Some comments for the new manuscript (which have surfaced to the provided extra information and the restructuring of the whole manuscript):

Major:

2.1 (i) As outlined above the flow of the paper would benefit from moving the drug testing to the end. Additionally it would benefit from making this figure more informative: use drug names instead of the "fitting" target (except confidential drugs). Do all drugs only target the indicated genes or do they have multiple targets among the candidate genes (or in the Pro-and anti-ED PINs)? A summary figure combining insights of the siRNA screen and drug test would be of benefit.

R.2.1 We have now moved the drug screening section to the last chapter of the results section as suggested. Additionally, we merged all the information in Table EV1, which lists all the targets from each drug, the filtering criteria, and added a summary figure (Figure 4D & Fig EV6G) presenting the combined results of the siRNA and drug screens for the overlapping targets. For simplicity of interpretation we included the drug-to-target map as Figure 4C instead of replacing the target names for drug names.

2.2 (ii) Figure EV1 D: Even though the materials and methods section states now the RNAseq data has been batch-corrected, there seems to be a remaining batch effect for OSS as well as LSS/FSS samples. Where does this stem from? Crucially this could influence the differential gene expression results if not accounted for in the DESeq2 design formular (i.e. provided as an extra factor).

R.2.2 Indeed in Figure EV1 D it is possible to see two subclusters for OSS and LSS. Specifically for this hierarchical cluster, where we merged the experiment of two labs, we considered as a batch the experiment of each group. However, when performing the differential expression analysis, we accounted for the batches within each dataset. Specifically, in the one in figure EV1 D, we discovered that the batch was related to the lane where the sequencing was made. Here is how we accounted for the batch during the analysis:

```
dds <- DESeqDataSetFromMatrix(countData = raw_cts,
                    colData = coldata_shear,
                    design= ~ batch + Condition)
```

Minor:

2.3 (1) Figure EV1 D: Text always refers to LSS but the samples are indicated as FSS here, what was the correct treatment?

R.2.3 Both terms, laminar shear stress (LSS) and fluid shear stress (FSS), are correctly and commonly used within the endothelial cell biology community. To maintain consistency, we have updated all the texts to use "LSS" throughout the document.

2.4 (2) Figure 2 and 3: - General: Color scale of Z-score is not symmetric around 0 (i.e. 0 is not white). Please correct in figures A-E. - In Ranking Score plots what do the two dots per gene in the figure represent, a confidence interval? I could not find an explanation for this anywhere.

R.2.4 All the Z-score scale plots were updated for the figures mentioned. Also, the legend contains the explanation for the Ranking Score plots. In summary, each gene is represented by two dots, as the score ranking was performed twice. The first ranking (left to right, TRAF1 to JUNB) ranks genes by Z-score values: high for ICAM1 and ROS, low for nuclei counts (DAPI) and VE-cadherin. These are pro-ED genes that, when silenced, increase ICAM1 and ROS while decreasing VE-cadherin and DAPI. The second ranking (right to left) is for anti-ED genes. Genes are ranked inversely: low for ICAM1 and ROS, high for DAPI and VE-cadherin. The visualisation order follows the first ranking, which is why blue dots may not appear as sequential. We added this information in the figure legend.

2.5 (3) Regarding the ChEMBL and OPenTarget drugs selection: I am missing a list that summarizes the used drugs along with target, predicted Kd/Ki or IC50 and other filtering

criteria. Manually checking, I could not recapitulate that IL6 is a binding target for prednisolone.

R.2.5 Table EV1 now contains all the requested information. Also, the molecules were initially identified in the ChEMBL database. For instance, the ID we used for IL6 CHEMBL1795129, which based on this molecule we arrive at the ZINC formula (ZINC000003833821) of prednisolone. Methods was updated.

2,6 (4) Page 7: VE cadherin staining genes states inconsistent numbers (21 in total, 20 decreasing and 3 increasing)

R.2.6 We thank the reviewer for their observation. Indeed, the plot was outdated. We have updated Figure 2E and the text.

2.7 (5) Figure EV5 F: What do the authors show in the three confusion tables if not the frequencies required for the hypergeometric test. What is the interpretation of positive and negative residuals with two digits behind the comma? What is the circle size indicating?

R.2.7 We apologise for the lack of clarity. The plots depict contingency tables for each hypergeometric test. In these tables, values with two decimal places indicate residuals. A positive residual suggests that the observed values were more frequent than expected, while a negative residual indicates the opposite: that the observed values were less frequent than expected. To clarify further, the first two plots indicate that the nodes from both the pro-anti-ED network and the anti-ED network were significantly more frequently observed in the differentially expressed genes (DEGs) from ECs in atherosclerosis plaques than expected. However, this was not the case for the nodes in the pro-ED network. We have updated the figure legend to reflect this.

2.8 (6) Typos:

- page 25 CellProfiler (r missing)

- Legend Figure 2 and 3 "and nd nuclei" (perhaps you meant "nb" instead

of nd?)

- Legend EV1: "motif sequence at and enrichment log p" (remove "at"?)

- Figure EV2 C: "ration" instead of ratio

R.2.8 We have now corrected all the typos identified and read carefully through the text to identify additional ones.

Reviewer #3:

In this study, Pinheiro-de-Sousa et al aim to provide a foundation for constructing ED disease networks and identifying key node genes that have the potential to be therapeutic targets for cardiovascular diseases.

Their approach is still dominantly computational, but in the revised version of the manuscript they have incorporated more experimental data, both from gene silencing

and pharmacological in vitro experiments. Owing to the removal of computational knockout analyses from the manuscript, which was an exciting and novel systems biology approach, this revised version is slightly less innovative in the technical sense, but not any less interesting and important. The identification and systematic silencing of 81 core ED genes, where 68 genes were shown to have a significant impact on at least one of the ED readouts, is a confident and very important result for the field that will likely constitute a basis for further functional and mechanistic studies of these genes by other groups. Moreover, none of the identified genes appeared to be essential in the absence of the pathological stimuli, suggesting their specific relevance to cell survival and function in the context of ED. Also, the Authors demonstrated that the commonly used practice of identifying deregulated genes in single omics datasets, may result in erroneous targeting of non-causal processes, potentially exacerbating the disease phenotype.

In addition to the fact that the manuscript is very well written, presented and coherent, as well as being technically sound, the Authors have answered all my previous comments and requests and made suitable revisions. Taking everything together, I believe that their findings highlight the potential of integrating omics datasets from diverse conditions, to distinguish causal from adaptive processes and prioritise targets for therapeutic modulation of these phenotypes.

We thank the reviewer for their time and positive comments.

23rd Oct 2023

Manuscript number: MSB-2022-11462RR
Title: Integrated Systems Biology Approach Identifies Gene Targets for Endothelial Dysfunction

Dear Evangelia,

Thank you again for sending us your revised manuscript. We are now satisfied with the modifications made and I am pleased to inform you that your paper has been accepted for publication.

*** PLEASE NOTE *** As part of the EMBO Publications transparent editorial process initiative (see our Editorial at https://dx.doi.org/10.103/msb.2010.72), Molecular Systems Biology publishes online a Review Process File with each accepted manuscripts. This file will be published in conjunction with your paper and will include the anonymous referee reports, your point- by-point response and all pertinent correspondence relating to the manuscript. If you do NOT want this File to be published, please inform the editorial office at msb@embo.org within 14 days upon receipt of the present letter.

LICENSE AND PAYMENT:
All articles published in Molecular Systems Biology are fully open access: immediately and freely available to read, download and share.

Molecular Systems Biology charges an article processing charge (APC) to cover the publication costs. You, as the corresponding author for this manuscript, should have already received a quote with the article processing fee separately. Please let us know in case this quote has not been received.

Once your article is at Wiley for editorial production, you will receive an email from Wiley's Author Services system, which will ask you to log in and will present you with the publication license form for completion. Within the same system the publication fee can be paid by credit card, an invoice or pro forma can be requested.

Payment of the publication charge and the signed Open Access Agreement form must be received before the article can be published online.

Upon acceptance it is mandatory for you to return the completed payment form. Failure to send back the form may result in a delay in the publication of your paper.

Molecular Systems Biology articles are published under the Creative Commons licence CC BY, which facilitates the sharing of scientific information by reducing legal barriers, while mandating attribution of the source in accordance to standard scholarly practice.

Proofs will be forwarded to you within the next 2-3 weeks.

Thank you very much for submitting your work to Molecular Systems Biology.

Kind regards,

Maria

Maria Polychronidou, PhD
Senior Editor
Molecular Systems Biology

EMBO Press Author Checklist

Corresponding Author Name: Evangelia Petsalaki, Jose E. Krieger
Journal Submitted to: Molecular System Biology
Manuscript Number: MSB-2022-11462

USEFUL LINKS FOR COMPLETING THIS FORM
The EMBO Journal - Author Guidelines
EMBO Reports - Author Guidelines
Molecular Systems Biology - Author Guidelines
EMBO Molecular Medicine - Author Guidelines

Reporting Checklist for Life Science Articles (updated January
This checklist is adapted from Materials Design Analysis Reporting (MDAR) Checklist for Authors. MDAR establishes a minimum set of requirements in transparent reporting in the life sciences (see Statement of Task: 10.31222/osf.io/9sm4x). Please follow the journal's guidelines in preparing your
Please note that a copy of this checklist will be published alongside your article.

Abridged guidelines for figures
1. Data
The data shown in figures should satisfy the following conditions:
- the data were obtained and processed according to the field's best practice and are presented to reflect the results of the experiments in an accurate and unbiased manner.
- ideally, figure panels should include only measurements that are directly comparable to each other and obtained with the same assay.
- plots include clearly labeled error bars for independent experiments and sample sizes. Unless justified, error bars should not be shown for technical
- if n<5, the individual data points from each experiment should be plotted. Any statistical test employed should be justified.
- Source Data should be included to report the data underlying figures according to the guidelines set out in the authorship guidelines on Data

2. Captions
Each figure caption should contain the following information, for each panel where they are relevant:
- a specification of the experimental system investigated (eg cell line, species name).
- the assay(s) and method(s) used to carry out the reported observations and measurements.
- an explicit mention of the biological and chemical entity(ies) that are being measured.
- an explicit mention of the biological and chemical entity(ies) that are altered/varied/perturbed in a controlled manner.
- the exact sample size (n) for each experimental group/condition, given as a number, not a range;
- a description of the sample collection allowing the reader to understand whether the samples represent technical or biological replicates (including how many animals, litters, cultures, etc.).
- a statement of how many times the experiment shown was independently replicated in the laboratory.
- definitions of statistical methods and measures:

  - common tests, such as t-test (please specify whether paired vs. unpaired), simple χ2 tests, Wilcoxon and Mann-Whitney tests, can be unambiguously identified by name only, but more complex techniques should be described in the methods section;

  - are tests one-sided or two-sided?
  - are there adjustments for multiple comparisons?
  - exact statistical test results, e.g., P values = x but not P values < x;
  - definition of 'center values' as median or average;
  - definition of error bars as s.d. or s.e.m.

Please complete ALL of the questions below.
Select "Not Applicable" only when the requested information is not relevant for your study.

Materials

Newly Created Materials	Information included in the manuscript?	In which section is the information available? (Reagents and Tools Table, Materials and Methods, Figures, Data Availability Section)
New materials and reagents need to be available; do any restrictions apply?	Not Applicable	

Antibodies	Information included in the manuscript?	In which section is the information available? (Reagents and Tools Table, Materials and Methods, Figures, Data Availability Section)
For **antibodies** provide the following information:<br>- Commercial antibodies: RRID (if possible) or supplier name, catalogue number and or/clone number<br>- Non-commercial: RRID or citation	Yes	Material and methods - anti-ICAM1 antibody, Cell Signaling, #62133 anti-E-selectin antibody, Abcam, #ab18991/ HIF-1a, Cell Signaling, #36169/GAPDH, Abcam, #ab22555

DNA and RNA sequences	Information included in the manuscript?	In which section is the information available? (Reagents and Tools Table, Materials and Methods, Figures, Data Availability Section)
**Short novel DNA or RNA including primers, probes**: provide the sequences.	Yes	Materia and methods pag. 26 for the genes ALDOA, LDHA, CXCR4 and NOS3

Cell materials	Information included in the manuscript?	In which section is the information available? (Reagents and Tools Table, Materials and Methods, Figures, Data Availability Section)
**Cell lines:** Provide species information, strain. Provide accession number in repository **OR** supplier name, catalog number, clone number, and/**OR** RRID.	Yes	Materia and methods pag. 21 for Human Aorta Artery Endothelyal Cells (HAECs) purchased from LONZA
**Primary cultures:** Provide species, strain, sex of origin, genetic modification status.	Not Applicable	
Report if the cell lines were recently **authenticated** (e.g., by STR profiling) and tested for mycoplasma contamination.	Not Applicable	

Experimental animals	Information included in the manuscript?	In which section is the information available? (Reagents and Tools Table, Materials and Methods, Figures, Data Availability Section)
**Laboratory animals or Model organisms:** Provide species, strain, sex, age, genetic modification status. Provide accession number in repository **OR** supplier name, catalog number, clone number, **OR** RRID.	Not Applicable	
**Animal observed in or captured from the field:** Provide species, sex, and age where possible.	Not Applicable	
Please detail **housing and husbandry conditions**.	Not Applicable	

Plants and microbes	Information included in the manuscript?	In which section is the information available? (Reagents and Tools Table, Materials and Methods, Figures, Data Availability Section)
**Plants:** provide species and strain, ecotype and cultivar where relevant, unique accession number if available, and source (including location for collected wild specimens).	Not Applicable	
**Microbes:** provide species and strain, unique accession number if available, and source.	Not Applicable	

Human research participants	Information included in the manuscript?	In which section is the information available? (Reagents and Tools Table, Materials and Methods, Figures, Data Availability Section)
If collected and within the bounds of privacy constraints report on age, sex and gender or ethnicity for all study participants.	Not Applicable	

Core facilities	Information included in the manuscript?	In which section is the information available? (Reagents and Tools Table, Materials and Methods, Figures, Data Availability Section)
If your work benefited from core facilities, was their service mentioned in the acknowledgments section?	Not Applicable	

Design

Study protocol	Information included in the manuscript?	In which section is the information available? (Reagents and Tools Table, Materials and Methods, Figures, Data Availability Section)
If study protocol has been **pre-registered, provide DOI in the manuscript**. For clinical trials, provide the trial registration number **OR** cite DOI.	Not Applicable	
Report the **clinical trial registration number** (at ClinicalTrials.gov or equivalent), where applicable.	Not Applicable	

Laboratory protocol	Information included in the manuscript?	In which section is the information available? (Reagents and Tools Table, Materials and Methods, Figures, Data Availability Section)
Provide DOI OR other citation details if **external detailed step-by-step protocols** are available.	Not Applicable	

Experimental study design and statistics	Information included in the manuscript?	In which section is the information available? (Reagents and Tools Table, Materials and Methods, Figures, Data Availability Section)
Include a statement about **sample size** estimate even if no statistical methods were used.	Yes	Materia and methods pag. 25 and 27
Were any steps taken to minimize the effects of subjective bias when allocating animals/samples to treatment (e.g. **randomization procedure**)? If yes, have they been described?	Yes	Materia and methods pag. 25
Include a statement about **blinding** even if no blinding was done.	Not Applicable	
Describe **inclusion/exclusion criteria** if samples or animals were excluded from the analysis. Were the criteria pre-established?<br><br>If sample or data points were omitted from analysis, report if this was due to attrition or intentional exclusion and provide justification.	Yes	Materia and methods pag. 27
For every figure, are **statistical tests** justified as appropriate? Do the data meet the assumptions of the tests (e.g., normal distribution)? Describe any methods used to assess it. Is there an estimate of variation within each group of data? Is the variance similar between the groups that are being statistically compared?	Yes	Material and Methods pag. 25 to 27

Sample definition and in-laboratory replication	Information included in the manuscript?	In which section is the information available? (Reagents and Tools Table, Materials and Methods, Figures, Data Availability Section)
In the figure legends: state number of times the experiment was **replicated** in laboratory.	Yes	Figure 2 and 3; Figure EV2 to EV4; Appendix Figure S1. Material and Methods pag. 25 to 27
In the figure legends: define whether data describe **technical or biological replicates**.	Yes	Figure 2 and 3; Figure EV2 to EV4; Appendix Figure S1. Material and Methods pag. 25 to 27

Ethics

Ethics	Information included in the manuscript?	In which section is the information available? (Reagents and Tools Table, Materials and Methods, Figures, Data Availability Section)
Studies involving **human participants**: State details of **authority granting ethics approval** (IRB or equivalent committee(s), provide reference number for approval.	Not Applicable	
Studies involving **human participants**: Include a statement confirming that **informed consent** was obtained from all subjects and that the experiments conformed to the principles set out in the WMA Declaration of Helsinki and the Department of Health and Human Services Belmont Report.	Not Applicable	
Studies involving **human participants:** For publication of **patient photos**, include a statement confirming that consent to publish was obtained.	Not Applicable	
Studies involving experimental **animals**: State details of **authority granting ethics approva**l (IRB or equivalent committee(s), provide reference number for approval. Include a statement of compliance with ethical regulations.	Not Applicable	
Studies involving **specimen and field samples:** State if relevant **permits** obtained, provide details of authority approving study; if none were required, explain why.	Not Applicable	

Dual Use Research of Concern (DURC)	Information included in the manuscript?	In which section is the information available? (Reagents and Tools Table, Materials and Methods, Figures, Data Availability Section)
Could your study fall under dual use research restrictions? Please check biosecurity documents and list of **select agents and toxins** (CDC): https://www.selectagents.gov/sat/list.htm	Not Applicable	
If you used a select agent, is the security level of the lab appropriate and reported in the manuscript?	Not Applicable	
If a study is subject to dual use research of concern regulations, is the name of the **authority granting approval and reference number** for the regulatory approval provided in the manuscript?	Not Applicable	

Reporting

The MDAR framework recommends adoption of discipline-specific guidelines, established and endorsed through community initiatives. Journals have their own policy about requiring specific guidelines and recommendations to complement MDAR.

Adherence to community standards	Information included in the manuscript?	In which section is the information available? (Reagents and Tools Table, Materials and Methods, Figures, Data Availability Section)
State if relevant guidelines or checklists (e.g., **ICMJE, MIBBI, ARRIVE, PRISMA**) have been followed or provided.	Not Applicable	
For **tumor marker prognostic studies,** we recommend that you follow the **REMARK** reporting guidelines (see link list at top right). See author guidelines, under 'Reporting Guidelines'. Please confirm you have followed these guidelines.	Not Applicable	
For **phase II and III randomized controlled trials**, please refer to the **CONSORT** flow diagram (see link list at top right) and submit the CONSORT checklist (see link list at top right) with your submission. See author guidelines, under 'Reporting Guidelines'. Please confirm you have submitted this list.	Not Applicable	

Data Availability

Data availability	Information included in the manuscript?	In which section is the information available? (Reagents and Tools Table, Materials and Methods, Figures, Data Availability Section)
Have **primary datasets** been deposited according to the journal's guidelines (see 'Data Deposition' section) and the respective accession numbers provided in the Data Availability Section?	Not Applicable	
Were **human clinical and genomic datasets** deposited in a public access-controlled repository in accordance to ethical obligations to the patients and to the applicable consent agreement?	Not Applicable	
Are **computational models** that are central and integral to a study available without restrictions in a machine-readable form? Were the relevant accession numbers or links provided?	Yes	Data and code availability section pag. 31
If publicly available data were reused, provide the respective **data citations in the reference list**.	Yes	Material and methods pag. 19
