# Supplementary material for: Integrated systems biology approach identifies gene targets for endothelial dysfunction
Source: Mol Syst Biol. 2023 Nov 30;19(12):e11462. doi: 10.15252/msb.202211462 (PMC10698507; doi:10.15252/msb.202211462)
Supplement: Supplementary file 10 — Source Data for Expanded View and Appendix [file MSB-19-e11462-s002.zip › Source Data for Expanded View.zip!/Source Data for Expanded View/Source_data_figure_EV2/README.rtf]

Files to reproduce figure EV2

This repository contains 4 tables for ploting the boxplot of ICAM1, ROS, VE-Cadherin and Nuleci counts.
