# Supplementary material for: Integrated systems biology approach identifies gene targets for endothelial dysfunction
Source: Mol Syst Biol. 2023 Nov 30;19(12):e11462. doi: 10.15252/msb.202211462 (PMC10698507; doi:10.15252/msb.202211462)
Supplement: Supplementary file 10 — Source Data for Expanded View and Appendix [file MSB-19-e11462-s002.zip › Source Data for Expanded View.zip!/Source Data for Expanded View/Source_data_figure_EV5/README.rtf]

Files to reproduce figure EV6

This repository contains 3 table. One table contain the combined nodes from pro- and anti-ED to generate the network. And the two other tables  for enrichment analysis for BP
